# Novel Palladium(II) β-Diketonate Complexes: Synthesis, Dual Antimicrobial/Anticancer Activity, and Molecular Insights into DNA/Protein Interactions

**DOI:** 10.3390/ijms27219437

**Published:** 2026-10-23

**Authors:** Marija Trifunović Stevanović, Nenad Joksimović, Jelena Petronijević, Snežana Stojanović, Slađana Đorđević, Jelena Antić Stanković, Biljana Bufan, Dragana D. Božić

**Affiliations:** 1 Department of Chemistry, Faculty of Science, University of Kragujevac, Radoja Domanovića 12, 34000 Kragujevac, Serbia; 2 Department of Microbiology and Immunology, Faculty of Pharmacy, University of Belgrade, Vojvode Stepe 450, 11221 Belgrade, Serbia

**Keywords:** palladium(II) complexes, β-diketo ester, antimicrobial activity, anticancer activity, DNA and protein binding study, molecular docking

## Abstract

This study reports the synthesis and biological evaluation of four novel palladium(II) complexes (**B1**–**B4**) with *O*,*O*-bidentate β-diketo ester ligands. Spectroscopic characterization confirmed their structures, while biological assays revealed substituent-dependent activity. Complexes **B3** and **B4** showed the most consistent antimicrobial activity, with minimum inhibitory concentrations (MICs) of 312–1200 μM across the tested microorganisms. **B3** exhibited the highest antiproliferative potency, with IC_50_ values of 2.3, 5.2, and 5.4 μM against K-562, HeLa, and HaCaT cells, respectively. However, its selectivity varied with the cell model, reaching a selectivity index (SI) of 5.17 against K-562 cells relative to MRC-5 fibroblasts and 2.35 relative to HaCaT cells. **B2** showed a more moderate activity profile, with some selectivity towards K-562 cells. Flow-cytometric analysis revealed increased sub-G0/G1 populations following treatment, consistent with the induction of apoptosis. Biophysical assays and molecular docking indicated structure-dependent interactions with DNA, while fluorescence studies revealed differences in the apparent binding affinities of the complexes toward human serum albumin (HSA). These findings highlight the influence of ligand structure on the biological activity and biomolecular interactions of palladium(II) complexes, identifying **B3** as the most promising candidate for further investigation based on its antiproliferative potency and selectivity.

## 1. Introduction

The field of bioinorganic and medicinal chemistry has experienced a significant evolution over the last few decades, driven by the focused design and evaluation of transition metal complexes as effective anticancer agents [1]. Since the seminal discovery and clinical introduction of cisplatin, platinum(II)-based coordination compounds have remained the cornerstone foundation of systemic chemotherapy for various solid tumors [2]. Despite their proven therapeutic efficacy, the clinical application of standard platinum drugs is severely limited by several drawbacks, such as dose-limiting systemic toxicities including nephrotoxicity and neurotoxicity, as well as the rapid development of both intrinsic and acquired cellular resistance [3,4]. To sidestep these issues, modern research has increasingly shifted towards other transition metal centers, among which palladium(II) has emerged as a highly appealing option. Possessing the same d^8^ electronic configuration and a strong tendency to form square-planar complexes, palladium(II) centers share the structural and electronic attributes of their platinum(II) counterparts [5,6,7]. However, a significant chemical challenge in palladium chemistry is the ligand exchange kinetics, which are generally 10^3^ to 10^5^ times faster than those of platinum, often causing rapid, non-selective hydrolysis and premature inactivation by extracellular biomolecules before reaching the desired intracellular targets [5]. Therefore, the rational design of palladium(II)-based metallodrugs fundamentally relies on the careful selection of strong, structurally stable chelating organic frameworks that can provide thermodynamic stabilization and controlled kinetic reactivity [7].

Among the extensive collection of organic chelators explored for this stabilizing function, β-diketo esters and similar β-diketone variants are exceptionally adaptable, biologically significant structures that offer distinct coordination properties [8]. These bidentate ligands readily lose a proton to form a highly delocalized monoanionic conjugated system, which strongly coordinates to the palladium(II) center via the enolic and carbonyl oxygen atoms, creating an incredibly stable, conjugated six-membered chelate ring [9,10]. The principal benefit of the β-diketo ester framework is its exceptional structural flexibility. Systematic variations to the outer ester chains and aliphatic or cyclic alkyl groups allow the precise modification of the electronic density, total molecular size, and city of the resulting compound [11,12,13]. These subtle structural and stereochemical modifications directly influence the overall planarity, dipole moments, and surface topographies of the coordination sphere, which are crucial factors determining how these coordination compounds interact with complex extracellular fluids, pass through hydrophobic cellular membranes, and accommodate within the specific structural cavities of biological receptors [14,15,16]. By combining the unique kinetic features of palladium(II) with the stereochemical flexibility of β-diketo ester structures, a rational strategy can be devised to enhance cell permeability, reduce non-specific off-target interactions, and overcome the traditional resistance pathways that limit conventional platinum therapies [11,13,16].

Interactions between novel organic compounds, metal complexes, and deoxyribonucleic acid (DNA) represent a fundamental area of research in modern bioinorganic and medicinal chemistry, particularly due to their crucial role in the development of targeted therapeutic agents and fluorescent biosensors [17,18]. Understanding the binding modes, affinities, and stabilization effects of small molecules on nucleic acid conformation is essential for elucidating mechanisms of cytotoxicity, genotoxicity, and pharmacological activity [19,20]. In recent years, computational methods—most notably molecular docking simulations utilizing crystallographic structures such as the canonical B-DNA dodecamer (PDB ID: 1BNA) alongside various other DNA conformations—have become indispensable tools for predicting binding sites, interaction energies, and preferred binding geometries (intercalation vs. groove binding) [21]. These in silico models provide vital atomic-level insights that complement experimental photophysical and biological evaluations, guiding the rational design of advanced bioactive ligands with enhanced affinity and selectivity [22].

Parallel to nucleic acid interactions, the investigation of binding affinities between bioactive metal complexes and transport proteins (serum albumins, especially human serum albumin (HSA)) constitutes a critical pillar in pharmacological evaluation and drug delivery design [23,24]. As the most abundant proteins in human plasma, serum albumins act as versatile carriers for a wide array of endogenous and exogenous agents, profoundly influencing their pharmacokinetics, systemic distribution, and overall bioavailability [23]. Characterizing the non-covalent interactions, binding constants, and site-specific localization of therapeutic candidates provides essential insights into transport mechanisms and reserve capacity within the circulatory system [23]. Furthermore, combining spectroscopic techniques, such as fluorescence quenching and competitive site marker assays, with complementary molecular docking simulations enables researchers to map preferred binding pockets, thermodynamic stability, and intermolecular forces with high precision [25]. Together, these comprehensive evaluations facilitate the optimization of drug–protein interactions, ensuring that novel metallopharmaceuticals achieve an optimal balance between stable systemic transport and effective release at target pathological sites [25].

The primary objective of this study is to overcome the inherent kinetic instability and rapid ligand-exchange rates of palladium(II) complexes **B1**–**4** by employing structurally tunable β-diketo ester chelating ligands, while resolving the mechanistic ambiguity surrounding their DNA-binding profiles. Specifically, this research aims to determine how outer-sphere steric modifications influence thermodynamic stabilization, differentiate between classical intercalation and non-covalent groove binding through a multi-technique approach, and utilize molecular docking simulations to provide an atomic-level understanding of macromolecular recognition within both DNA matrices and HSA, thereby establishing robust structure–activity relationships that govern their overall biological activity.

## 2. Results and Discussion

### 2.1. Synthesis of Novel Palladium(II) Complexes ***B1***–***4***

**Scheme 1:**
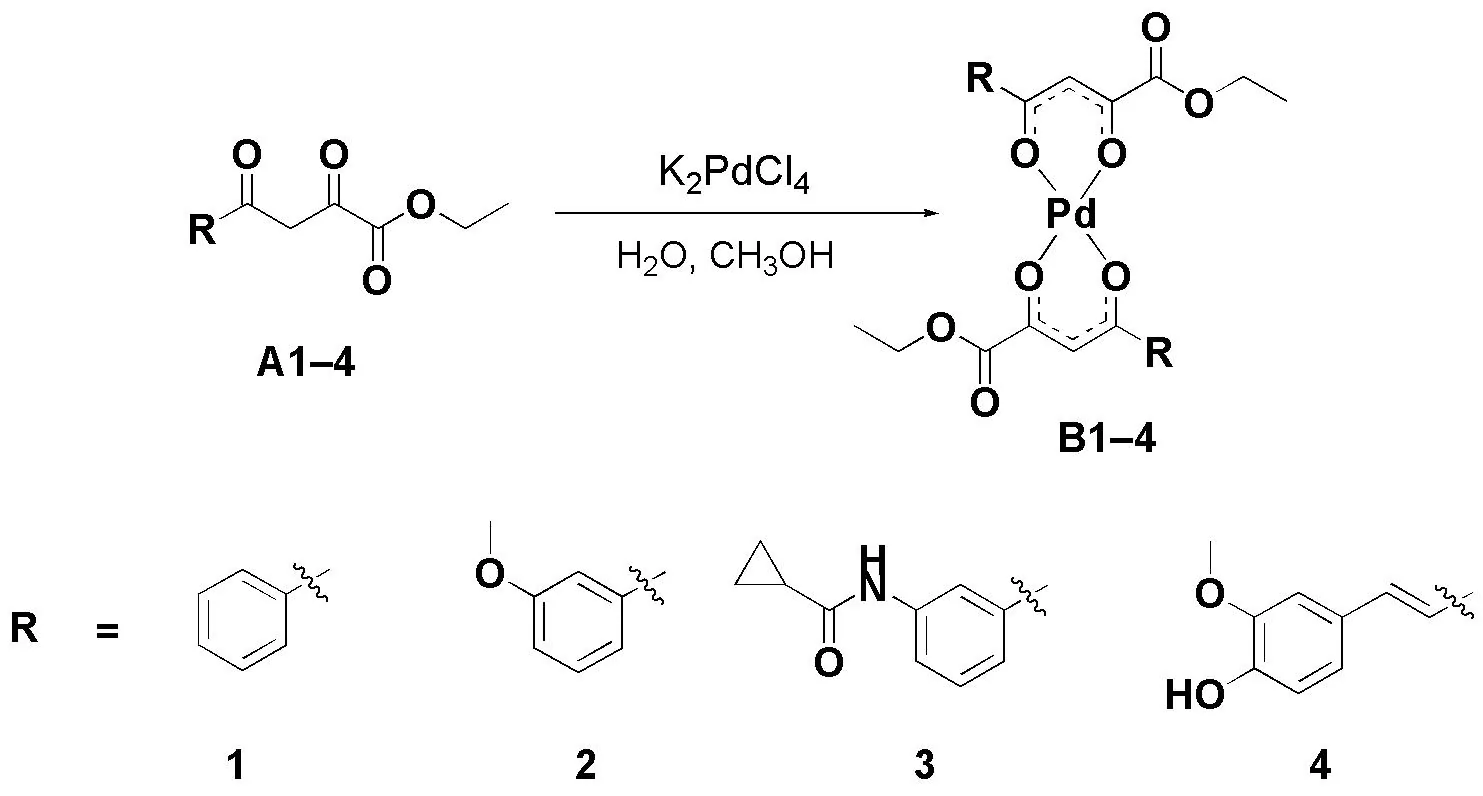
Synthetic pathway for the preparation of palladium(II) complexes **B1**–**4**.

The *O*,*O*-bidentate β-diketo ester ligands (**A1**–**4**) were previously synthesized via the Claisen condensation of diethyl oxalate with various methyl ketones under basic conditions [26,27,28]. Following the synthetic protocol established in our earlier study [29], the reaction of K_2_[PdCl_4_] with these ligands in a 1:1 (*v*/*v*) water/methanol mixture afforded four previously unreported palladium(II) complexes (**B1**–**4**), which are described herein for the first time (Scheme 1). In our previous work [29], the square-planar coordination geometry of analogous palladium(II) complexes with ligands of the same type was confirmed via X-ray crystallographic analysis. Accordingly, the newly synthesized complexes **B1**–**4** are expected to adopt a similar square-planar geometry. At room temperature, the onset of precipitation was observed within 30 s upon mixing the reagents. However, the reaction mixture was stirred for a total duration of 5 h to ensure complete conversion, affording the target complexes in high yields of up to 89%.

NMR spectra of the free ligands and their corresponding palladium(II) complexes were recorded in different deuterated solvents due to solubility differences. Although absolute chemical shifts are not directly comparable across solvents, significant spectral differences were observed between the ligands and complexes. Notably, the signal corresponding to the enolic –OH proton (δ ≈ 15 ppm) [26,27,29], present in the free ligands, disappears upon coordination, indicating metal binding (Figures S1–S8, ESI).

Infrared (IR) spectroscopy provided further insight into the coordination behavior of the β-diketo enol moiety. In the free ligands, strong bands around ~1590 cm^−1^ are attributed to conjugated *ν*(C=O)/*ν*(C=C) stretching vibrations arising from delocalized π-electrons across the β-diketo enol system. This intramolecular conjugation lowers the vibrational frequency relative to unconjugated C=O stretching (~1700 cm^−1^). Upon complexation with palladium(II), these bands shift to lower wavenumbers (~1560 cm^−1^), consistent with decreased carbonyl bond order due to electron donation to the metal center. Additionally, the strong enolic *ν*(C–O) bands, observed near ~1250 cm^−1^ in the free ligands, shift to higher wavenumbers (~1280 cm^−1^) in the complexes, indicating coordination through the enolic oxygen, likely following deprotonation [30,31,32].

Together, these spectroscopic changes confirm that palladium(II) coordinates through both the enolic and carbonyl oxygens, forming stable six-membered chelate rings. These observations are consistent with the proposed structures and are supported by elemental analysis data, confirming the stoichiometry of the synthesized complexes.

In addition, these complexes exhibited good stability in PBS buffer at a concentration of 1.35 × 10^−5^ M, with virtually no changes in peak intensities or positions over 24 h (Figure S9, ESI).

### 2.2. Determination of Antimicrobial potential

The antimicrobial activity of complexes **B1**–**4** was evaluated by determining their minimum inhibitory concentrations (MICs) against a panel of nine laboratory control strains, including Gram-positive and Gram-negative bacteria, one yeast, and one anaerobic bacterium. The MIC values are summarised in Table 1.

Complexes **B1**–**4** exhibited moderate antimicrobial activity across Gram-positive, Gram-negative, fungal, and anaerobic strains. The strongest activity was observed for complexes **B3** and **B4**, which consistently produced lower MIC values than complexes **B1** and **B2**. Against *Staphylococcus aureus*, compounds **B3** and **B4** inhibited growth at 312 µM, whereas complexes **B1** and **B2** required 625 µM. *Staphylococcus epidermidis* and *Enterococcus faecalis* showed uniform susceptibility to all complexes (312 µM).

Gram-negative bacteria were less susceptible overall. Complexes **B1** and **B2** exhibited MICs of 800–1200 µM, while complexes **B3** and **B4** showed improved activity, particularly against *Escherichia coli* and *Klebsiella pneumoniae* (400 µM). *Pseudomonas aeruginosa* remained the least sensitive organism, with MICs of 800–1200 μM for all complexes.

For the fungal and anaerobic strains, *Candida albicans* showed an MIC of 625 µM for complex **B1**, whereas complexes **B2**–**4** inhibited growth at 312 µM and *Cutibacterium acnes* showed a similar pattern, with complexes **B2** and **B4** demonstrating the strongest activity (312 µM).

Overall, **B3** and **B4** generally exhibited the most favorable antimicrobial profiles, particularly against several Gram-positive and Gram-negative bacteria. However, their activity varied among the tested microorganisms, and no single complex was consistently the most active against all strains.

The antimicrobial activity of palladium(II) complexes **B1**–**4** is probably related to their geometry and to interactions with structural and functional components of bacterial and fungal cells.

As previously described, all complexes share a square-planar Pd(II) center coordinated through the enolic and carbonyl oxygens of β-diketo ligands, forming a rigid six-membered chelate ring. This geometry increases the electrophilicity of the Pd(II) center, enabling interactions with biological nucleophiles (thiols, amines, phosphates) [33], enhances planarity, facilitating binding to DNA [34], and increases lipophilicity, improving penetration through the cell membrane [33]. The differences in antimicrobial potency among complexes **B1**–**4** correlate strongly with substituent effects. Complexes **B1** and **B2** contain substituents that maintain higher polarity and lower hydrophobicity, limiting membrane permeation and reducing activity, especially against Gram-negative bacteria with relatively impermeable outer membranes. Complex **B3** contains a cycloalkane moiety, increasing hydrophobic surface area and conformational rigidity. This enhances membrane interaction and may facilitate passage through lipid-rich barriers, explaining its superior activity against all tested strains of microorganisms, except *P. aeruginosa*. Furthermore, lipophilicity experiments confirmed the highest reactivity for ligand **A3**. These observations are consistent with established structure–activity relationships for β-diketone metal complexes, in which increased lipophilicity generally promotes interactions with microbial membranes and enhances antimicrobial activity through improved cellular uptake [35,36].

The antimicrobial activity of palladium(II) complexes can be attributed to several mechanisms, such as membrane disruption and increased permeability, interactions with thiol-containing enzymes, interactions with DNA and interference with replication, and protein binding and sequestration.

The increased lipophilicity of palladium(II) complexes is generally considered to facilitate interaction with and permeation across bacterial membranes, thereby enhancing antimicrobial activity [37]. This mechanism is consistent with the stronger activity of **B3** and with the higher susceptibility of lipid-rich organisms, such as *C. albicans* and *C. acnes*. Because Pd(II) is a soft Lewis acid with high affinity for sulfur donors, palladium(II) complexes can coordinate to cysteine residues in thiol-containing proteins, potentially impairing the activity of enzymes involved in bacterial redox homeostasis and metabolism [37], or inhibiting essential membrane transport proteins. The square-planar geometry and electrophilicity of Pd(II) facilitate these interactions. Although the complexes do not deeply intercalate, their ability to bind DNA can interfere with replication of microorganisms, disrupt the transcription of mRNA, and destabilize DNA–protein interactions [37,38]. Complex **B3**, with the highest *K*_b_, is likely to exert stronger DNA-related effects. The strong binding to human serum albumin—HSA (especially by **B1**) suggests that these complexes can interact with bacterial proteins in an analogous manner, potentially inhibiting DNA-binding proteins, ribosomal subunits, and metabolic enzymes essential for bacterial survival and viability, as well as for the synthesis of structural components.

The observed differences in antimicrobial activity against Gram-positive and Gram-negative bacteria, as well as the yeast *C. albicans*, also reflect differences in cell envelope architecture. Gram-positive bacteria possess a thick peptidoglycan cell wall but lack the outer membrane characteristic of Gram-negative bacteria, which generally makes their cell envelope more permeable to many antimicrobial compounds [39,40]. The lower antimicrobial activity of complexes **B1**–**4** against Gram-negative bacteria correlates with their cell structure, as these bacteria possess an outer membrane rich in lipopolysaccharides, a periplasmic space rich in enzymes, various types of porins that restrict the entry of hydrophobic molecules (particularly in *P. aeruginosa*), and multidrug efflux pumps that expel antimicrobial agents from cells before they reach their intracellular targets and exert antimicrobial effects [40,41]. The susceptibility of *C. albicans* to palladium(II) complexes is also influenced by the unique architecture of its cell envelope. Unlike bacteria, *C. albicans* possesses a multilayered cell wall composed primarily of β-glucans, chitin, and mannoproteins, which surrounds a plasma membrane rich in ergosterol. Although this cell wall provides structural protection, it is generally more permeable to small coordination compounds than the outer membrane of Gram-negative bacteria [42]. Thus, **B1** and **B2**, which are more polar, show weak activity, whereas **B3** and **B4**, which are more hydrophobic, penetrate more effectively. Complex **B3** also exerts the highest DNA-binding constant, which may correlate with its antimicrobial activity. The antimicrobial activity of **B1** could result from its strong HSA binding and highest ethidium bromide (EB) displacement, with partial intercalation capability. Although the present study demonstrates promising antimicrobial activity, additional investigations focusing on biofilm inhibition and other virulence-associated processes are required to further elucidate the antimicrobial mechanisms of the most active palladium(II) complexes.

### 2.3. In vitro cytotoxicity evaluation

**Figure 1:**
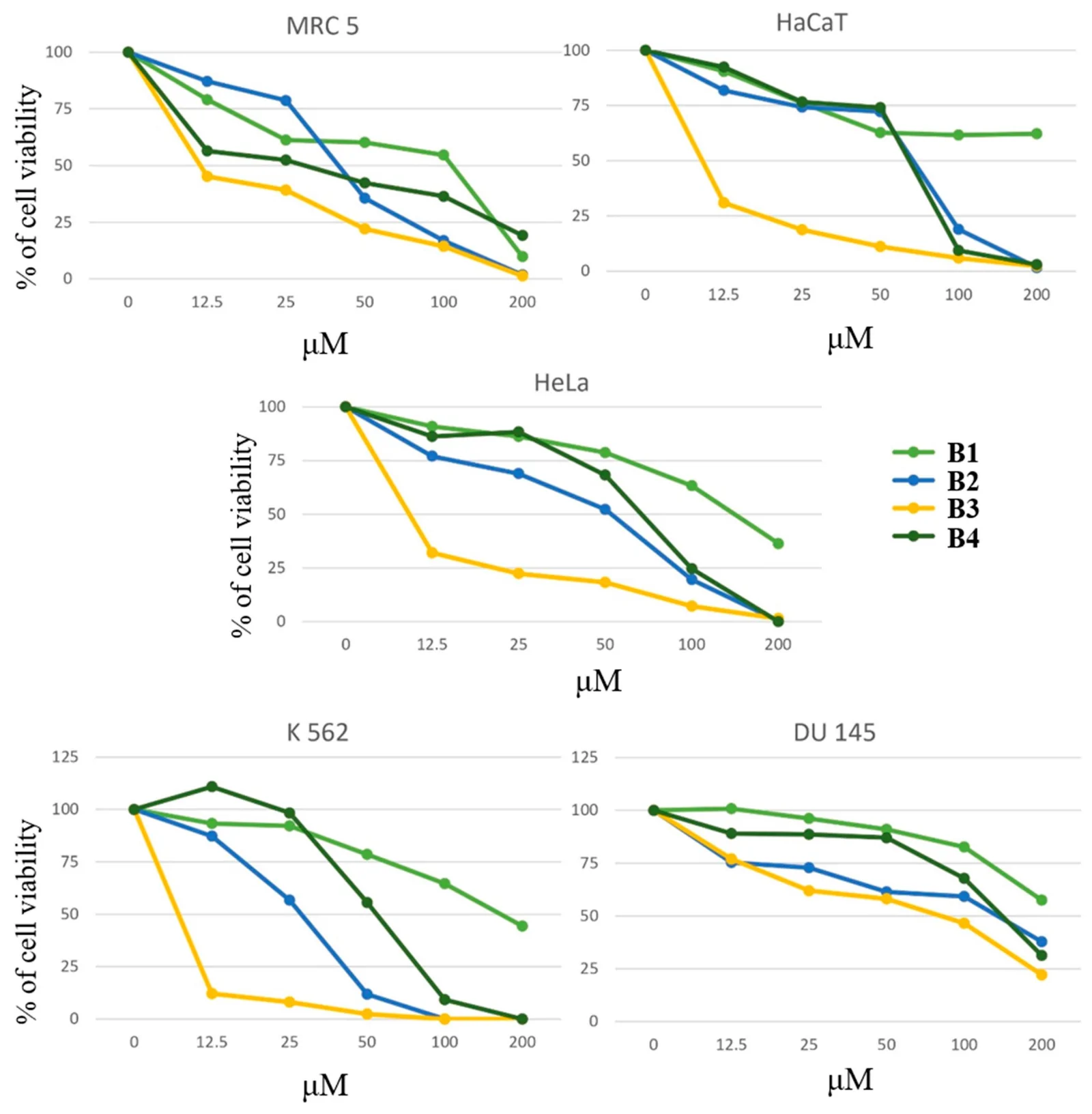
The effect of **B1**–**4** on normal and malignant cell lines. MRC-5 (normal human foetal lung fibroblasts), HaCaT (immortalised normal human keratinocytes), HeLa (human cervical carcinoma), K-562 (human chronic myelogenous leukaemia), and DU-145 (human prostate carcinoma).

**Figure 2:**
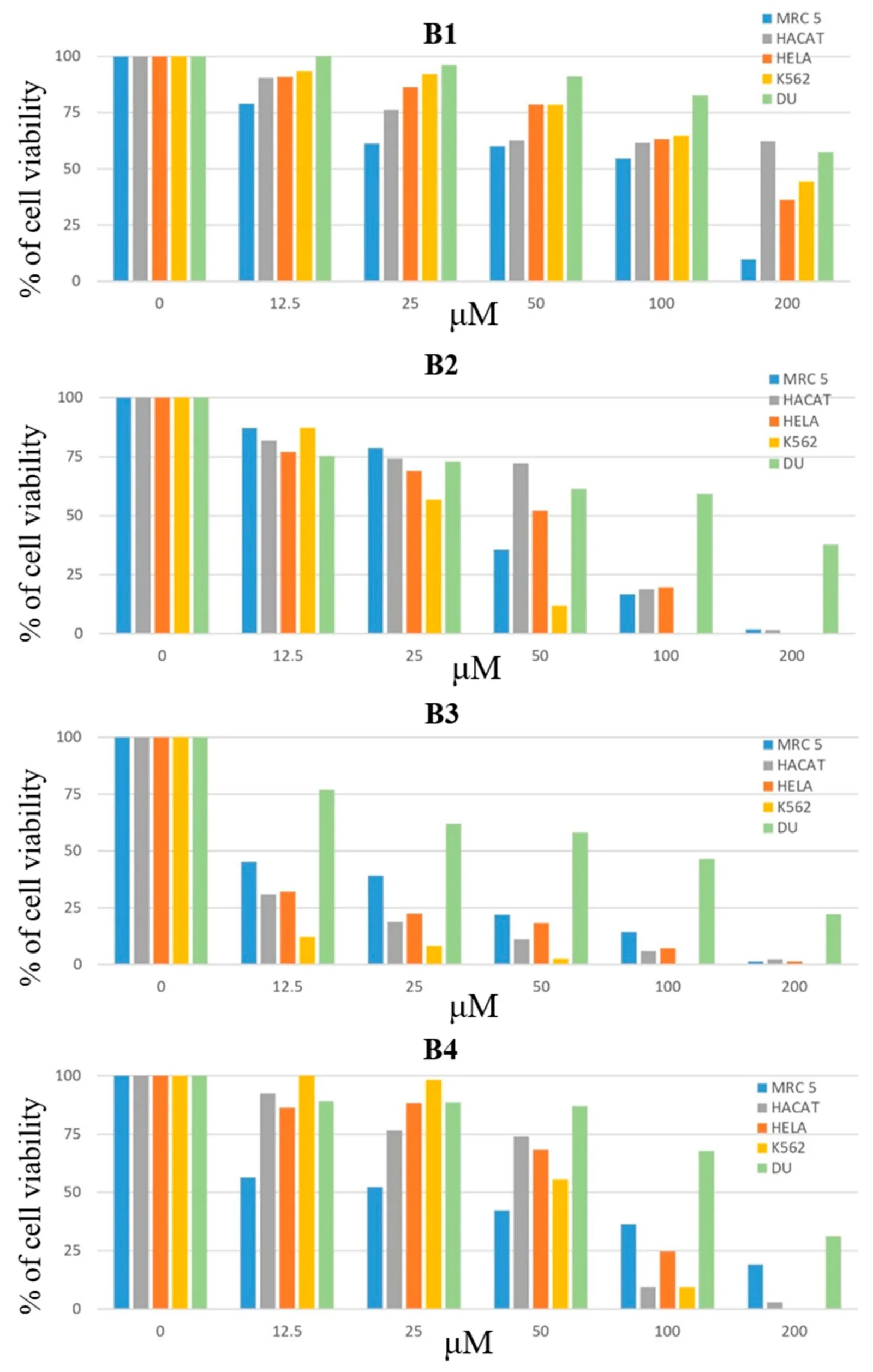
The effect of **B1**–**4** on different cell lines. MRC-5 (normal human foetal lung fibroblasts), HaCaT (immortalised normal human keratinocytes), HeLa (human cervical carcinoma), K-562 (human chronic myelogenous leukaemia), and DU-145 (human prostate carcinoma).

The cytotoxic effects of **B1**–**4** were evaluated against two normal cell lines (MRC-5 and HaCaT) and three malignant cell lines (HeLa, K-562 and DU-145) following 72 h exposure. The concentration–response data showed compound- and cell line-dependent effects. Nonlinear regression analysis was used to estimate concentrations causing 50% inhibition of cell viability (IC_50_ values) from the concentration–response curves. The IC_50_ values and 95% CIs are summarized in Table 2, while the full concentration–response profiles are shown in Figure 1 and Figure 2.

**B3** exhibited the lowest IC_50_ values among the tested complexes in several cell lines, with estimated IC_50_ values of approximately 5.2 μM in HeLa, 5.4 μM in HaCaT, and 2.3 μM in K-562. **B2** also showed pronounced cytotoxicity, particularly against K-562, MRC-5, and HeLa cells, with IC_50_ values of approximately 26.6 μM, 40.8 μM and 42.2 μM, respectively. **B4** showed intermediate activity in most cell lines, whereas **B1** generally exhibited weaker cytotoxic effects.

Against MRC-5 cells, the estimated IC_50_ values were 61.6 μM, 40.8 μM, 11.9 μM, and 25.6 μM for **B1**–**4**, respectively. In HaCaT cells, **B1** did not reach 50% inhibition within the tested concentration range and was therefore assigned an IC_50_ value > 200 μM, whereas **B2**–**4** showed IC_50_ values of approximately 57.6 μM, 5.4 μM, and 60.8 μM, respectively. A similar trend was observed in HeLa and K-562 cell lines for **B1**–**4**. In HeLa cells, the highest estimated IC_50_ value was observed for **B1** (139.2 μM) and the lowest for **B3** (5.2 μM), while in K-562 cells the values were 165.1 μM for **B1** and 2.3 μM for **B3**. For DU-145 cells, **B1** also did not reach 50% inhibition at 200 μM, while **B2**–**4** showed IC_50_ values of approximately 120.3 μM, 61.4 μM, and 138.4 μM, respectively.

Comparison of malignant cell lines (Figure 2) revealed distinct sensitivity patterns. K-562 cells were the most responsive to **B2** and **B3**, while HeLa cells showed high susceptibility to **B3** and moderate sensitivity to **B2** and **B4**. In contrast, DU-145 cells consistently displayed the highest resistance to all investigated complexes, with IC_50_ values ranging from 61.4 to >200 μM.

Normal cell lines did not exhibit lower sensitivity than the malignant cell lines. Both MRC-5 fibroblasts and HaCaT keratinocytes were highly susceptible to **B3** and moderately susceptible to **B2** and **B4**, indicating limited selectivity of these complexes for malignant cells. Within the present series, **B3** generally exhibited the highest cytotoxic potency, followed by **B2** and **B4**, whereas **B1** showed the lowest activity, while overall cellular susceptibility was approximately K-562 ≈ HeLa > MRC-5 ≈ HaCaT >> DU-145.

To contextualize the cytotoxicity of the **B1**–**4** complexes, the reference chemotherapeutics doxorubicin and cisplatin were considered. Numerous studies and review articles consistently report that both drugs exert measurable cytotoxic effects in HeLa, K562, DU 145, MRC-5 and HaCaT cells within low- to mid-micromolar concentration ranges, with malignant cell lines generally more sensitive than non-malignant fibroblasts and keratinocytes [43,44,45,46,47]. This indicates that doxorubicin and cisplatin display quantifiable cytotoxicity across the same experimental window (≤200 µM) used in our assays, allowing the cytotoxicity profiles of **B1**–**4** to be interpreted qualitatively in relation to established chemotherapeutic benchmarks.

The selectivity indices (SI) of the palladium(II) complexes were determined using both MRC-5 and HaCaT cells as normal cell models (Table 3). An SI > 1.0 indicates a drug whose efficacy against tumor cells is greater than its toxicity towards normal cells, i.e., the compound has considerable anticancer specificity, whereas an SI < 1.0 indicates non-selective action [48,49]. Although SI > 1 reflects favorable selectivity, SI values ≥ 2–3 are generally considered more indicative of promising anticancer selectivity during in vitro screening.

Overall, the investigated palladium(II) complexes exhibited cell-line-dependent selectivity. **B3** showed the most favorable selectivity towards K-562 cells, with SI values of 5.17 and 2.35 relative to MRC-5 and HaCaT cells, respectively. For HeLa cells, their selectivity was less pronounced, with SI values of 2.29 relative to MRC-5 and 1.04 relative to HaCaT cells, indicating little difference in sensitivity between HeLa and HaCaT cells. **B2** also showed moderate selectivity towards K-562 cells, with SI values of 1.53 and 2.17 relative to MRC-5 and HaCaT cells, respectively. In contrast, **B1**-**4** all showed limited selectivity towards DU-145 cells when MRC-5 cells were used as the reference (SI < 1). For **B1**, the SI relative to HaCaT cells could not be reliably determined for DU-145 because the IC_50_ values for both cell lines exceeded the highest tested concentration. Overall, these findings indicate that the antiproliferative potency of the complexes does not necessarily translate into broad selectivity towards malignant cells.

The cytotoxicity studies demonstrated marked differences in the antiproliferative activities of the investigated palladium(II) complexes. Among the four compounds, **B3** exhibited the highest cytotoxic potency against almost all tested cell lines, followed by **B2** and **B4**, whereas **B1** was consistently the least active. The observed cytotoxicity trends partially parallel those obtained in the antimicrobial assays, suggesting that structural features of the coordinated β-diketonate ligands may contribute to the biological activity of the complexes. Increased lipophilicity of metal complexes generally facilitates passive diffusion across the plasma membrane and enhances intracellular accumulation, thereby increasing the probability of interactions with biologically important targets such as DNA, proteins and mitochondria [37,50,51].

Unlike platinum(II) drugs, whose primary mechanism involves DNA crosslinking, palladium(II) complexes are considerably more labile because of their faster ligand-exchange kinetics. Consequently, many palladium(II) compounds exert their biological activity through multiple mechanisms, including interactions with DNA and proteins, coordination to thiol-containing proteins and depletion of intracellular thiols, inhibition of enzymes involved in cellular redox homeostasis, mitochondrial dysfunction, and the generation of reactive oxygen species (ROS), ultimately leading to apoptotic cell death [52,53,54]. Palladium(II), as a soft Lewis acid, exhibits a high affinity for sulphur-containing biomolecules such as glutathione and cysteine residues in proteins, leading to disruption of essential cellular processes [37,51]. Therefore, the pronounced cytotoxicity of **B3** most likely results from a combination of favorable physicochemical properties that promote cellular uptake together with efficient interactions with intracellular molecular targets.

Although **B3** exhibited the highest antiproliferative potency among the investigated complexes, its selectivity was cell-line dependent. The most favorable selectivity was observed against K-562 cells (SI = 5.17 and 2.35 relative to MRC-5 and HaCaT, respectively), whereas selectivity against HeLa cells was limited when HaCaT cells were used as the reference (SI = 1.04). Conversely, **B2** exhibited a moderate yet balanced selectivity profile, particularly against K-562 cells, yielding SI values of 1.53 (MRC-5) and 2.17 (HaCaT). These observations indicate that targeted modifications of the β-diketonate ligand scaffold systematically modulate both antiproliferative efficacy and cellular selectivity. Overall, the pronounced selectivity observed for **B3** highlights its potential as a promising lead candidate, warranting further structural optimization and mechanistic characterization.

The markedly higher sensitivity of K-562 cells compared with solid tumor cell lines is consistent with previous studies demonstrating that hematological malignancies are often more susceptible to metal-based anticancer agents than epithelial cancers, largely because of their rapid proliferation, greater propensity to undergo apoptosis, and the absence of the complex tumor microenvironment that contributes to drug resistance in solid tumors [55,56]. K-562 cells possess high proliferative activity and relatively limited extracellular matrix protection, allowing more efficient exposure to cytotoxic compounds. In addition, alterations in redox regulation and elevated oxidative stress make leukaemia cells particularly vulnerable to metal complexes capable of disturbing thiol homeostasis or inducing ROS formation [37,51,53].

In contrast, DU-145 cells consistently exhibited the greatest resistance to all investigated complexes. This prostate carcinoma cell line is well known for its intrinsic resistance to numerous chemotherapeutic agents, including platinum-based metallodrugs [57,58]. Several mechanisms may contribute to this phenotype, including increased expression of ATP-binding cassette (ABC) efflux transporters, elevated intracellular glutathione levels, enhanced antioxidant capacity, efficient DNA repair systems and defects in apoptosis signaling pathways [55,59]. Collectively, these mechanisms reduce intracellular drug accumulation and attenuate the cytotoxic effects of electrophilic metal complexes. The consistently high IC_50_ values obtained for DU-145 therefore suggest that these palladium(II) complexes are unable to overcome the intrinsic resistance mechanisms of this cell line.

**B1** exhibited the weakest cytotoxic activity, with IC_50_ values exceeding 100 μM in most cell lines and no measurable IC_50_ in HaCaT or DU-145 cells within the tested concentration range. These findings suggest that the ligand environment of **B1** provides insufficient stabilization and/or lipophilicity to promote efficient cellular uptake or productive interactions with intracellular targets, consistent with previous reports demonstrating that the biological activity of palladium(II) complexes is strongly influenced by ligand structure and physicochemical properties [37,51]. Together with the antimicrobial data, the present results further indicate that subtle structural modifications of the β-diketonate ligand substantially affect the biological properties of these palladium(II) complexes.

Overall, the present results indicate that cytotoxic potency follows the order **B3** > **B2** ≈ **B4** > **B1**. Importantly, the selectivity index (SI) analysis revealed that high cytotoxic potency in this series can be successfully aligned with target selectivity. Among the investigated complexes, **B3** exhibited the highest antiproliferative potency and the most favorable selectivity towards K-562 cells.

### 2.4. The Effect of Palladium(II) Complexes ***B1***–***4*** on Cell-Cycle

Evaluation of the antiproliferative/cytotoxic activity of palladium(II) complexes **B1**–**4** and their ability to induce perturbation of the cell-cycle was performed in HeLa cells treated with IC_50_ and IC_50_/2 concentrations of the compounds. The cells were further subjected to propidium iodide (PI) staining and analysis using flow cytometry. This method allows the measurement of DNA content and distinction between cell-cycle phases: sub-G0/G1 (apoptotic) cells, and G0/G1, S and G2/M phases of the cell-cycle.

**Figure 3:**
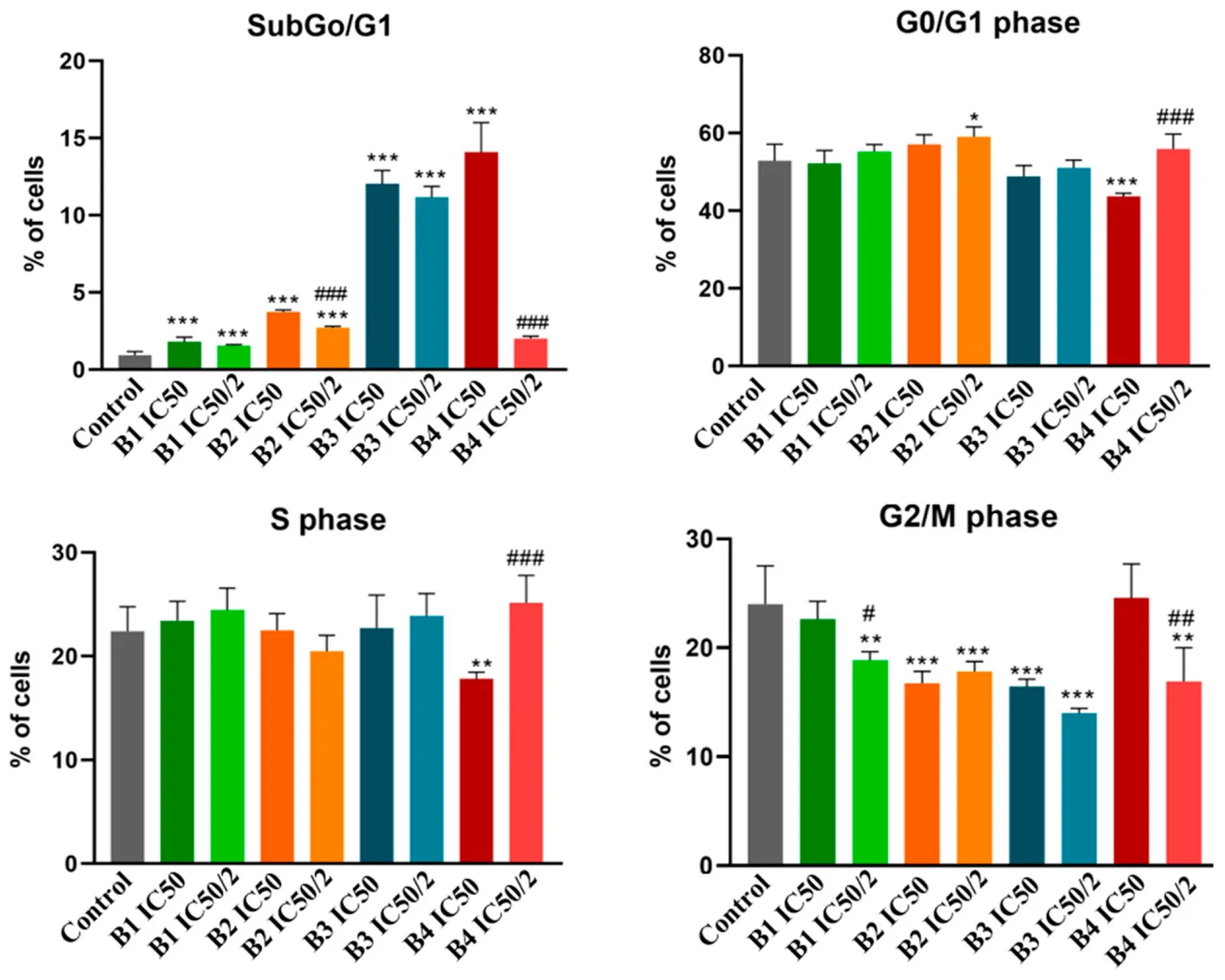
Effects of palladium(II) complexes on cell-cycle distribution of HeLa cells. Cells were treated with IC_50_ and IC_50_/2 concentrations of the tested **B1**–**4** complexes for 48 h, stained with propidium iodide, and analyzed by flow cytometry. Data are presented as mean ± standard deviation (SD) of independent experiments. * *p* < 0.05, ** *p* < 0.01, *** *p* < 0.001 vs. untreated control; # *p* < 0.05, ## *p* < 0.01, ### *p* < 0.001 vs. IC_50_-treated cells.

Based on the obtained results, **B1** and **B2** exhibited a similar pattern of action, although the effects of **B2** were more pronounced. The effects of these compounds were characterized by a modest increase in the percentage of the sub-G0/G1 population and a slight decrease in the percentage of cells in the G2/M phase compared to untreated cells. The distribution of cells in the G0/G1 and S phases was not significantly affected compared to the untreated control (Figure 3).

**B3** had qualitatively the same effect, but substantially stronger. It caused approximately a 12-fold increase in the sub-G0/G1 population, accompanied by a decrease in the percentage of cells in the G2/M phase and no change in the G0/G1 and S populations, compared to untreated cells (Figure 3).

Concerning **B4**, treatment with the IC_50_ concentration resulted in the most pronounced increase in the percentage of sub-G0/G1 population compared to untreated cells. This effect was accompanied by a significant decrease in the percentage of cells in the G0/G1 and S phases of the cell-cycle. Notably, **B4** exhibited a concentration-dependent effect. It is interesting that this compound exhibited a concentration-dependent effect. Specifically, treatment with the lower concentration of **B4** (IC_50_/2) resulted only in a decrease in the percentage of cells in the G2/M phase, with no significant difference in the proportion of the sub-G0/G1 population or other cell-cycle populations compared to the untreated control. The concentration-dependent changes in cell-cycle distribution observed for **B4** are consistent with the previously reported dose-dependent cytotoxic activity of structurally diverse palladium(II) complexes in cancer cell models, including HeLa cells [60,61].

Our results demonstrate that all tested palladium(II) complexes exhibited a common pattern of cell-cycle perturbation in HeLa cells, although differences in the magnitude and distribution of the observed changes were evident. They predominantly increased the sub-G0/G1 population, which is commonly associated with DNA fragmentation during apoptosis [62]. The most prominent increase was detected in **B3** and **B4** IC_50_-treated cultures, and this finding is in line with the results obtained from antimicrobial and cytotoxic studies, as described above. Similar apoptosis-associated effects have been reported by other authors who tested palladium(II) complexes bearing different ligands and exhibiting diverse structural features in various cancer cell lines [60,61,63,64,65,66]. The proposed mechanisms are diverse and may depend on the structure of the palladium(II) complex, as well as on the cellular model and experimental conditions. They include the induction of DNA double-strand breaks [67], generation of reactive oxygen species [67], mitochondrial damage and activation of the intrinsic apoptotic pathway [64,68,69].

Of particular relevance, a series of palladium(II) complexes containing a β-diketonate ligand in combination with 2,2′-bipyridine have also been reported to exhibit cytotoxic activity. One of these complexes was shown to induce ROS accumulation, mitochondrial membrane potential collapse, G2/M cell-cycle arrest and apoptosis in HeLa cells after 24 h of incubation [70]. This finding demonstrates that palladium(II) complexes containing β-diketonate ligands may induce apoptosis through mechanisms involving both oxidative stress and cell-cycle perturbation. However, the observed G2/M arrest differs from the results obtained in our study, further indicating that the cellular response to palladium(II) complexes may vary depending on their structural features and experimental conditions.

Another important finding is that none of the investigated complexes induced a pronounced accumulation of cells in a particular cell-cycle phase. This suggests that their cytotoxic effects were not primarily associated with a strong phase-specific cell-cycle arrest but may involve the induction of apoptosis. Consistent with this finding, Kacar et al. [63] reported that palladium(II) saccharinate complexes induced apoptosis in different cancer cell lines and that their cytotoxic effects were cell-cycle-independent [63]. On the other hand, investigation of the effects of structurally different palladium(II) complexes, e.g., palladium(II) Schiff-base [61], cyclopalladated palladium(II) complexes [64], palladium(II) complex bearing a polydentate N-donor ligand [66], palladium(II) selenosemicarbazone complex [60] showed their ability to induce cell-cycle arrest in some of the phases. From a therapeutic perspective, both phase-specific cell-cycle arrest and apoptosis without pronounced cell-cycle arrest may be relevant mechanisms of anticancer activity. While cell-cycle arrest can suppress tumor-cell proliferation, apoptosis may induce cancer cell death independently of the cell-cycle phase. One therapeutic strategy is to combine agents with different mechanisms of action to achieve synergistic effects [71].

Overall, the tested palladium(II)—complexes exhibited predominantly cell-cycle independent cytotoxic activity in HeLa cells, as evidenced by the increased proportion of cells in the sub-G0/G1 population without marked changes in the distribution of cells across the other cell-cycle phases. Complexes **B3** and **B4** showed the most pronounced effects.

### 2.5. Determination of Lipophilicity

Lipophilicity plays a pivotal role in the cellular internalization of drug candidates, thereby significantly influencing their design and optimization [72,73]. This physicochemical property is commonly quantified by the octanol–water partition coefficient (*log P*), which can be experimentally evaluated using the traditional flask-shaking technique [74]. The determined *log P* values for the investigated ligands **A1**–**4** are 5.06, 5.41, 5.45 and 3.31, respectively. All obtained values fall well within the established optimal *log P* range of −0.4 to 5.6 defined for novel pharmacophores in comprehensive medicinal chemistry [75,76]. Furthermore, the higher *log P* magnitudes observed for ligands **A2** and **A3** point to their preferential partitioning into the octanol phase, which implies enhances membrane affinity and potentially greater cellular uptake efficiency compared to the others.

### 2.6. DNA-Interactions

#### 2.6.1. UV-Vis Spectroscopic Studies

Investigating the interactions between small molecules and DNA plays a fundamental role in pharmacological research, particularly in the evaluation of novel metal-based anticancer agents. Given this significance, the interaction of the synthesized palladium(II) complexes with DNA warrants detailed investigation.

DNA–ligand interactions can be broadly categorized as covalent or non-covalent. Covalent binding involves the direct interaction of a molecule with the phosphate backbone or nitrogenous bases of DNA, often leading to irreversible modifications. In contrast, non-covalent interactions include intercalation between base pairs, electrostatic attraction to the negatively charged phosphate groups, and groove binding within the major or minor grooves of the DNA double helix [77]. These non-covalent modes rely on the inherent conformational flexibility and dynamic helical framework of B-DNA, which accommodates small molecules without disrupting the overall double-stranded structure [78].

Electronic absorption (UV-Vis) spectroscopy is a widely utilized technique for probing the DNA-binding properties of metal complexes [15]. Although UV-Vis spectroscopy alone does not definitively identify the mode of binding, it provides valuable preliminary evidence for DNA interaction. In particular, the occurrence of hypochromic or hyperchromic effects upon addition of DNA can suggest intercalative or electrostatic binding, respectively [13].

Intercalative binding of small molecules to DNA typically involves π–π* stacking interactions between the aromatic chromophores of the ligand and the base pairs of DNA. This interaction often leads to hypochromism and a bathochromic shift (red shift) in the absorption spectra. It is generally accepted that the extent of hypochromism observed in the UV absorption band correlates with the strength of intercalative interaction [79,80].

In the present study, solutions were prepared by maintaining a fixed concentration of the palladium(II) complexes (1.35 × 10^−5^ M) while incrementally increasing the concentration of calf thymus DNA—CT-DNA (ranging from 1.35 × 10^−5^ M to 1.35 × 10^−4^ M). The mixtures were incubated for 24 h at room temperature prior to recording the UV–Vis spectra. As illustrated in Figure S10 (ESI), the addition of CT-DNA resulted in a pronounced hyperchromic effect for all four complexes.

The intrinsic binding constants (*K*_b_) for complexes **B1**–**4** were calculated using the Wolfe–Shimmer equation and are presented in Table 4. The obtained *K*_b_ values are notably lower than those typically associated with classical intercalators, suggesting that the palladium(II) complexes interact with DNA via a non-intercalative, likely electrostatic or groove-binding mode [81].

As shown in Table 4, complex **B3** exhibits the highest *K*_b_ values among the tested complexes. This enhanced affinity may be attributed to its structural features, particularly the presence of a cycloalkane moiety in the side chain. In contrast, the other complexes lack this substituent, which suggests that the conformational rigidity and hydrophobic character of the cycloalkane ring in **B3** may facilitate more favorable interactions with the DNA helix.

#### 2.6.2. Fluorescence Displacement Studies

A significant enhancement of fluorescence is well documented for the planar and conjugated molecule EB upon its intercalation between the base pairs of double-stranded DNA [82]. In its free state, EB exhibits weak fluorescence; however, upon intercalation into DNA, its fluorescence intensity increases markedly due to the restricted environment and reduced solvent quenching.

It has been reported that the addition of a second molecule capable of binding to DNA can competitively displace EB from its intercalation site, resulting in a measurable decrease in fluorescence intensity [14,79]. This quenching effect is considered strong evidence of the second molecule’s ability to interact with DNA, particularly through intercalation. Published data support this mechanism, showing that when EB is displaced from the EB-DNA complex by another ligand, the observed reduction in fluorescence intensity suggests that intercalation is the dominant mode of DNA binding for that ligand [11,16].

**Figure 4:**
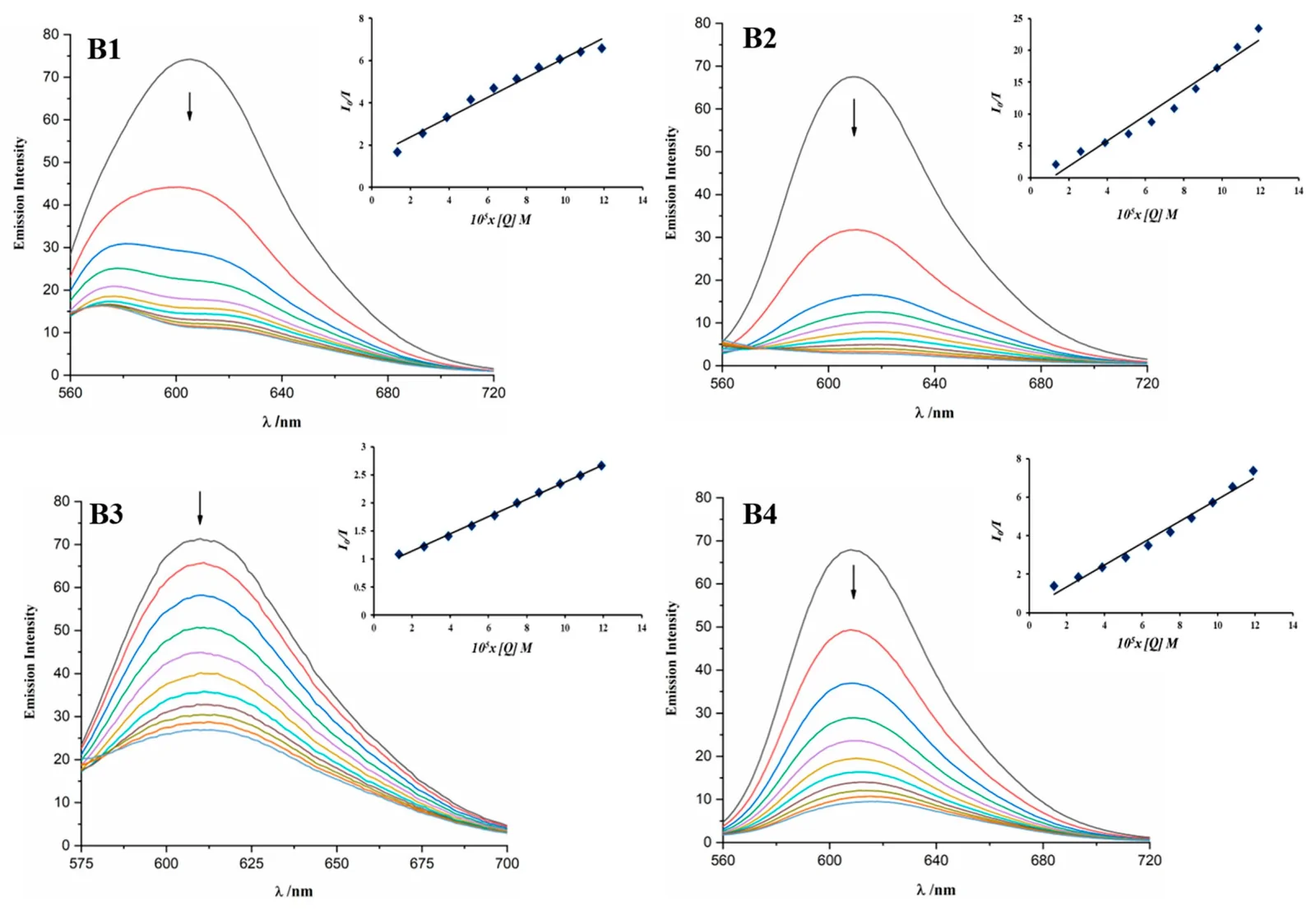
Emission spectra of EB-DNA with complexes **B1**–**4**. [**B1**–**4**] = (1.33–11.9 × 10^−5^ M); [EB] = [DNA] = 1.35 × 10^−5^ M. The arrows show the change in emission intensity after the addition of palladium(II) complexes. Insert graph: Plot of I_0_/I vs. Q.

A gradual decrease in fluorescence intensity in the range of 605–610 nm (depending on the complex) was observed upon titration of a fixed concentration of CT-DNA and EB (1.35 × 10^−5^ M each) with increasing concentrations of the palladium(II) complexes (1.33 × 10^−5^ to 1.19 × 10^−4^ M), as illustrated in Figure 4. This fluorescence quenching suggests potential intercalative interactions.

The *K*_SV_ constants, falling within the range of 10^4^ to 10^5^ M^−1^ (Table 4), strongly imply a high binding affinity toward DNA and suggest a potential intercalative mode of interaction. However, while values of this order of magnitude are frequently characteristic of intercalation, they are not definitively conclusive on their own, as high quenching constants can also arise from other strong binding modes. Consequently, to gain deeper insight into the exact binding mechanism and to resolve this ambiguity, viscosity measurements and molecular docking simulations were performed. The superior quenching efficiency of **B2** may be attributed to the presence of a methoxy substituent in its side chain, which could facilitate π–π stacking interactions with DNA base pairs.

Based on these findings, viscosity measurements were subsequently conducted for the **B2** complex to evaluate intercalation as a plausible binding mode.

#### 2.6.3. Evaluation of Viscosity Changes

To gain deeper insight into the DNA-binding modes of the investigated palladium(II) complexes (**B1**–**4**), hydrodynamic viscosity measurements were performed using complex **B2** as a representative derivative. Intercalation involves the partial insertion of aromatic heterocyclic rings between the base pairs of DNA, which typically results in an increase in the viscosity of the DNA solution [12]. However, upon addition of increasing concentrations of **B2** to the CT-DNA solution, only a negligible or very slight increase in relative viscosity was observed (Figure S11, ESI), which precludes a definitive conclusion of classical intercalation.

Given these ambiguous hydrodynamic outcomes, molecular docking simulations were subsequently conducted to comprehensively evaluate the potential binding interactions of the entire series (**B1**–**4)** with CT-DNA, providing complementary theoretical insights into their binding geometries and preferred interaction sites.

### 2.7. Protein Binding Profile of the Complexes

Constituting approximately 60% of total plasma content, HSA is the most abundant protein in vertebrates, with a normal physiological concentration of approximately 5 g/100 mL [83]. The three-dimensional structure of HSA has been extensively characterized by X-ray crystallography, revealing that it is a 66 kDa single-chain protein predominantly composed of α-helices [84].

HSA consists of three homologous domains (I, II, and III), each further divided into two subdomains (A and B), which are primarily α-helical and stabilized by numerous disulfide bridges. In total, the protein contains seventeen disulfide bonds, a single free thiol group at cysteine-34 (Cys34), and one tryptophan residue located at position 214 (Trp214) [85,86]. The overall architecture of HSA resembles a heart-shaped molecule, enabling its critical role in the transport and binding of a wide range of endogenous and exogenous ligands.

HSA serves as a key carrier protein in the bloodstream, capable of binding and transporting numerous poorly water-soluble drugs [82,87]. The binding of drugs to serum proteins significantly influences their pharmacokinetics and pharmacodynamics, affecting both activity and disposition. Moreover, the accumulation of serum proteins in tumor tissues has attracted attention for their potential use as drug delivery vehicles for anticancer agents. It has been demonstrated that macromolecules such as albumin and globulins preferentially accumulate in tumor tissues due to enhanced vascular permeability and reduced lymphatic drainage, leading to prolonged retention in the tumor interstitium [88]. The primary ligand-binding sites on albumin are located within hydrophobic cavities in subdomains IIA and IIIA, which share similar chemical properties [89].

**Figure 5:**
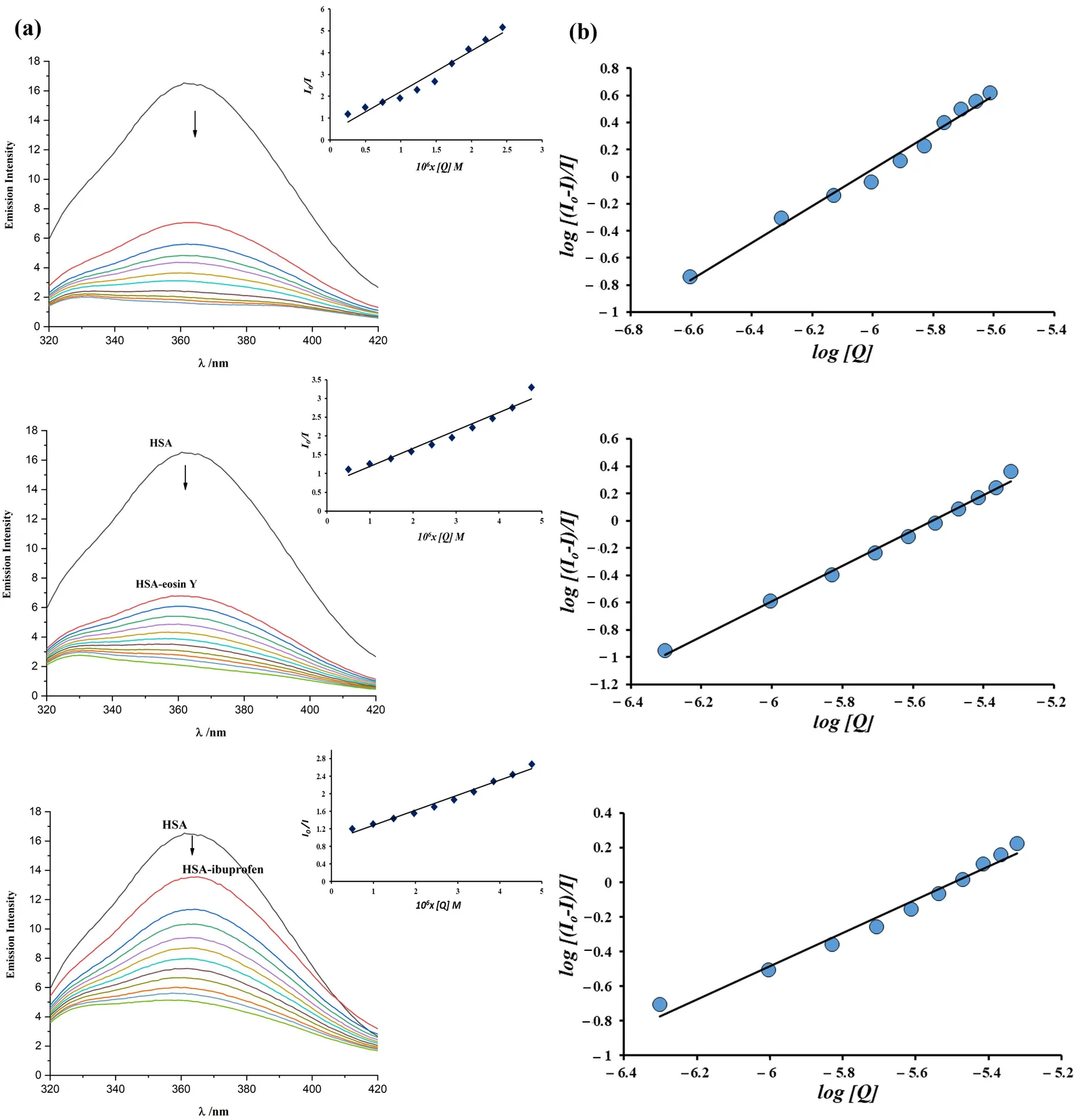
(**a**) Emission spectra of HSA/HSA—eosin Y/HSA—ibuprofen in the presence of complex **B2**. [**B2**] = 2.49–24.4 × 10^−7^ M, [HSA] = [eosin Y] = [ibuprofen] = 2.00 × 10^−6^ M. The arrows show the change in emission intensity after the addition of complex. Insert graph: plots of I_0_/I vs. [Q]. (**b**) Plot of log[(I_0_ − I)/I] vs. log[Q].

The addition of palladium(II) complexes **B1**–**4** to an HSA solution induced significant changes in the microenvironment of the tryptophan residues, as evidenced by a decrease in fluorescence intensity (Figure 5 and Figures S11–S13, ESI). This fluorescence quenching supports the hypothesis that **B1**–**4** interact with the HSA molecule. Among the tested complexes, **B2** exhibited the highest binding constant values, consistent with the observations from EB displacement experiments. Analysis of the data presented in Table 5 revealed a single binding site on human serum albumin (*n* ≈ 1) for all complexes. Furthermore, the quenching mechanism was determined to be static, as indicated by the bimolecular quenching constants (*k*_q_) ranging from 10^13^ to 10^14^ M^−1^ s^−1^, which exceed the maximum diffusion-controlled quenching rate constant for biomolecules (~2.0 × 10^10^ M^−1^ s^−1^).

The binding interactions of palladium(II) complexes **B1**–**4** with HSA were further investigated in the presence of site-specific markers, eosin Y and ibuprofen, which are known to selectively bind to subdomains IIA and IIIA, respectively [12]. Ibuprofen serves as a marker for site II located in subdomain IIIA, while eosin Y is a marker for site I within subdomain IIA.

As shown in Figure 5 and Figures S12–S14 (ESI), a significant reduction in fluorescence intensity was observed upon addition of **B1**–**4** in the presence of either eosin Y or ibuprofen, indicating that these complexes are capable of competing with the site markers and binding to HSA. The *K* constants calculated in the presence of eosin Y were lower compared to those obtained with ibuprofen, as summarized in Table 5. These findings suggest that site I (subdomain IIA) is the preferred binding locus for the palladium(II) complexes on the HSA molecule.

### 2.8. Quantum Chemical Calculations

**Figure 6:**
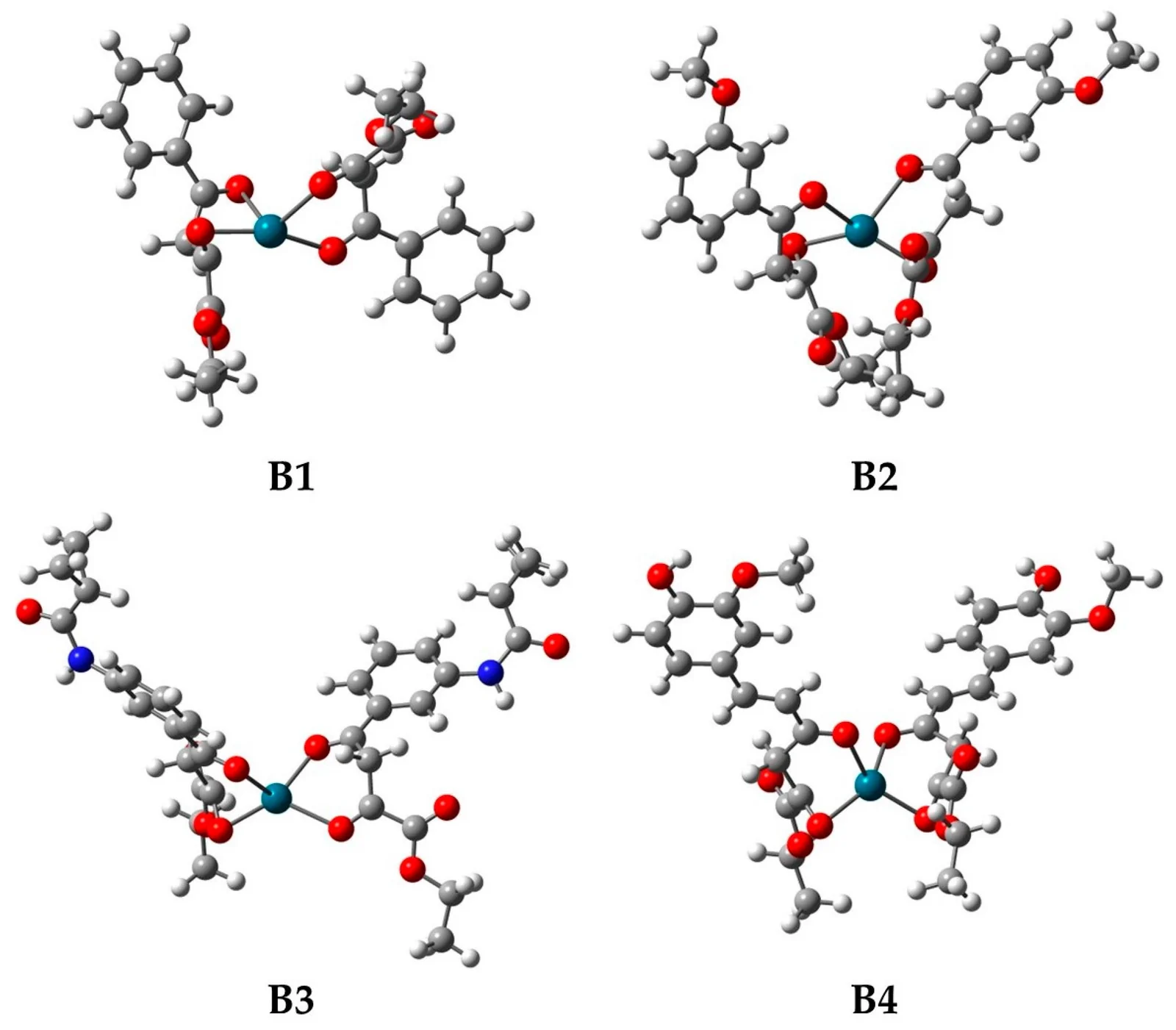
Optimized structures of studied complexes **B1**–**4** obtained at the B3LYP/def2-SVP level of theory. (Color coding for the structure: Pd (green), O (red), N (dark blue), C (gray), and H (white).)

Quantum chemical calculations were performed to gain deeper structural insights into the investigated complexes, with the resulting optimized geometries depicted in Figure 6. In all complexes, the Pd centers exhibit a distorted square-planar coordination geometry characterized by distinct Pd–O bond lengths measured by the BLA (bond length alternation) index in Table 6. To quantitatively evaluate the deviation of the square-planar geometry from ideal planarity, the molecular planarity parameter (MPP, in Å) and the span of deviation from the plane (SDP, in Å) were calculated for examined molecules (Table 6). The calculated MPP and SDP reveal varying degrees of structural distortion from the ideal square-planar geometry across the investigated palladium complexes. Among them, **B4** exhibits the highest degree of planarity, as evidenced by its remarkably low MPP and SDP values, indicating a well-accommodated and minimally strained coordination environment. Conversely, **B1** and **B3** display significant out-of-plane distortions. **B3** possesses the highest MPP and SDP value, while **B1** also exhibits a severe out-of-plane deviation with a high SDP value. These elevated values suggest substantial tetrahedral cramping or twisting of the square-planar framework, likely driven by steric hindrance imposed by the ligand scaffolds. **B2** occupies an intermediate position, reflecting a moderate structural deformation. The calculated BLA values align well with the geometric trends, reflecting the impact of spatial distortion on the electronic structure of the coordination sphere. **B4** displays the lowest BLA value, indicating highly symmetrical Pd-O bond lengths, consistent with its near-ideal square-planar geometry. In contrast, the substantially higher BLA values observed for **B1** and **B3** underscore a significant loss of local symmetry. The pronounced bond alternation in **B1** and **B3** suggests unequal perturbation of the coordination bonds, likely induced by the severe out-of-plane twisting (high MPP and SDP) and strong trans-influence within the asymmetric ligand environment.

### 2.9. Molecular Docking

**Figure 7:**
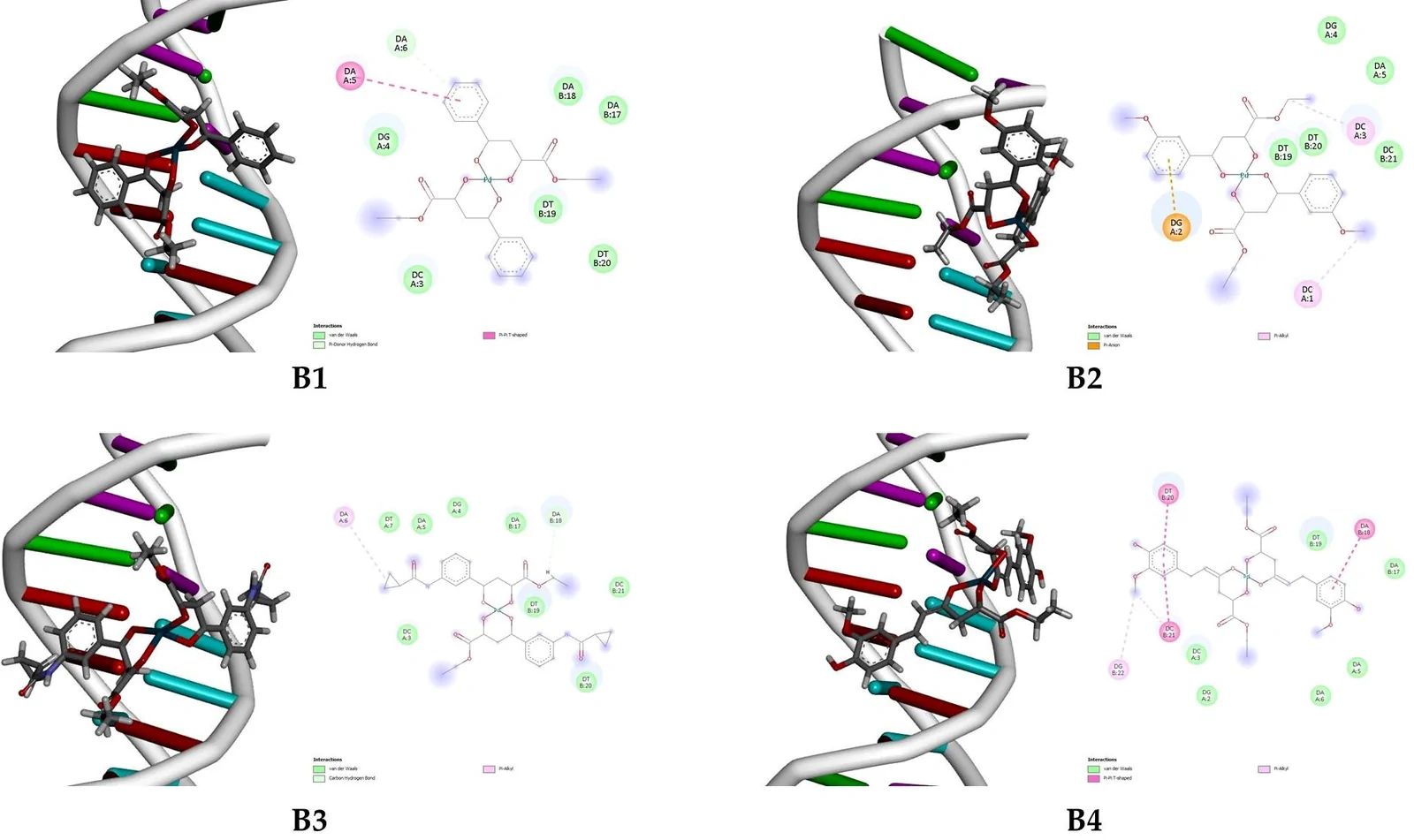
Best docking scenarios and 2D interaction diagram for complexes **B1**–**4** with canonical B-DNA (PDB ID: 1BNA).

Integrating molecular simulations with experimental data provides a powerful approach for probing drug interactions with DNA and HSA, identifying binding sites, and estimating binding affinities. Here, complexes **B1**–**4** were docked into target structures representing canonical B-DNA (PDB ID: 1BNA) and a conformation featuring an intercalation gap (PDB ID: 1Z3F). Figure 7 and Figure S15 display the lowest-energy docking poses, while Table 7 summarizes the corresponding scoring functions.

The molecular docking simulations and interaction analyses reveal a strong preference for minor groove binding over classical intercalation for the investigated palladium(II) complexes. The structural characteristics of the ligands, particularly the bulky and non-planar coordination sphere around the central Pd atom, dictate this non-intercalative mode of interaction with the DNA double helix (Figure 6 and Figure S15, ESI). The 3D conformational models demonstrate that the palladium complexes fit snugly into the narrow and shallow channel of the DNA minor groove. Instead of penetrating between the base pairs, the elongated structures of the ligands, along with their lateral ester groups and saturated ring systems, perfectly follow the helical curvature of the minor groove. The bulky geometry creates steric hindrance that physically prevents intercalation. Consequently, the native Watson–Crick base-pair stacking remains undisturbed, confirming that the complexes act as surface-seeking groove binders rather than intercalators. Crucially, the strongest binding affinity is achieved by the **B4** complex, which exhibits the least deviation from the ideal square-planar coordination geometry, thereby optimally facilitating these specific intermolecular contacts. The 2D interaction diagrams (Figure 7) highlight a diverse non-covalent interaction profile that energetically stabilizes the complexes within the groove. The primary driving force for groove binding is an extensive network of close-contact van der Waals interactions, where the ligands are deeply buried within the groove walls to maintain continuous contact with the sugar-phosphate backbone and the exposed edges of various nucleotides across all complexes (**B1**–**4**). This is further supported by specific π-interactions, as the terminal aromatic rings of the complexes establish critical contacts with the DNA bases, primarily manifesting as π-π T-shaped interactions and π-alkyl interactions, which are highly characteristic of minor groove binders interacting with the floor of the groove. Finally, electrostatic and hydrogen bonding interactions provide additional stabilization in specific conformations: **B2** is anchored via a π-anion interaction, **B1** forms a π-donor hydrogen bond, and **B3** displays a carbon hydrogen bond with the adjacent nucleotide residues.

As shown in Figure S15, there are no stable interactions between complexes **B1**–**4** and the DNA intercalation gap; consequently, no binding energy scores are reported for this region due to the absence of meaningful contact. Furthermore, because the DNA target was treated as a rigid structure, in line with established methodologies [3,11,12], the preservation of base-pair stacking represents a constraint of the rigid-receptor model rather than conclusive proof against intercalative binding. Therefore, the docking results support groove binding under the modeled conditions, without excluding other potential binding modes.

Investigation into the interactions between the synthesized palladium complexes and HSA provides critical insights into their pharmacokinetic profiles, particularly regarding systemic transport and bioavailability. To evaluate their preferential binding topography, competitive molecular docking simulations were conducted at two primary, well-characterized loci: Sudlow Site I (subdomain IIA), typically associated with eosin binding, and Sudlow Site II (subdomain IIIA), the canonical locus for ibuprofen. As summarized by the scoring functions in Table 7, all studied palladium complexes demonstrated a significantly higher binding affinity for HSA than the reference native ligands (eosin and ibuprofen). Furthermore, the complexes generally exhibited a preferential binding affinity toward Site I (subdomain IIA) over Site II (subdomain IIIA). Within Site I, complex **B3** displayed the strongest binding profile with a docking score of −15.7 kcal mol^−1^, representing highly favorable thermodynamic stabilization, followed closely by **B4** (−15.3 kcal mol^−1^) and **B1** (−14.9 kcal mol^−1^). Complex **B2** yielded a docking score of −13.2 kcal mol^−1^. Notably, all four metal complexes vastly outperformed the reference control eosin (−10.5 kcal mol^−^), confirming an exceptionally strong accommodating framework within subdomain IIA. Conversely, the binding affinities determined for Site II (subdomain IIIA) followed the descending order of **B3** (−11.3 kcal mol^−1^) > **B4** (−10.9 kcal mol^−1^) > **B1** (−10.4 kcal mol^−1^) > **B2** (−10.1 kcal mol^−1^), all demonstrating higher affinity than the reference ligand ibuprofen (−9.2 kcal mol^−^). Overall, while the examined palladium complexes demonstrate a distinct propensity to dock within the Site I cavity, **B3** stands out with the highest overall affinity across both binding domains.

**Figure 8:**
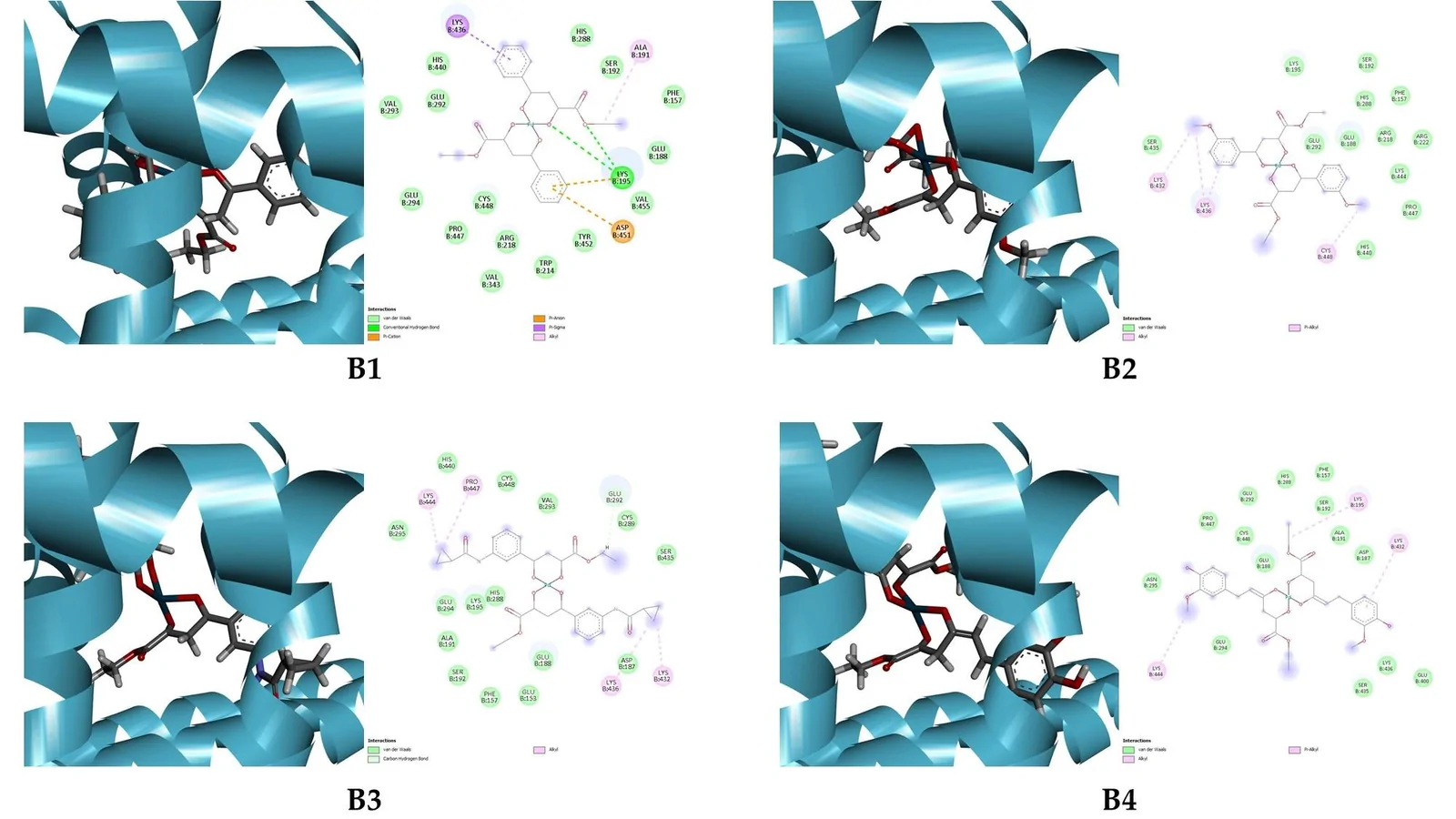
Best docking scenarios and 2D interaction diagram for complexes **B1**–**4** with Site I (subdomain IIA) in HSA (PDB ID: 2BXD).

The 2D intermolecular interaction maps (Figure 8) reveal distinct binding modes for the four palladium complexes (**B1**–**4**) within the central cavity of HSA, focusing on Sudlow’s Site I (subdomain IIA). Due to their elongated and bulky nature, these complexes adopt extended conformations, anchoring at the mouth of subdomain IIA while projecting into neighboring domains. Complexes **B2** and **B3** share an identical binding pose at the Domain I–Subdomain IIA interface. They are stabilized by Site 1 residues (Lys190, Ala194, Ser193, Arg197, and Arg145) via electrostatic and van der Waals forces, while their opposing segments extend into Domain I, interacting with a cluster spanning residues 108–115, His146, and Tyr148. This interdomain cleft mode allows the complexes to bridge the Domain I–II boundary. Complex **B4** docks specifically at the entrance of Sudlow’s Site 1, strongly anchored on its left flank by a prominent π-cation interaction with the gatekeeper residue Arg218 and a conventional hydrogen bond with Asn295 (subdomain IIB). Complex **B1** exhibits an alternative interdomain orientation, balancing between Subdomain IIA and Domain III. One terminal phenyl ring anchors at the Site I boundary via a π-cation interaction with Lys195, supported by nearby Arg218 and Arg222. Ultimately, while **B2** and **B3** lie across the Domain I/IIA interface, **B1** and **B4** more effectively exploit their elongated geometry through strong, directional interactions at the Site I entrance.

**Figure 9:**
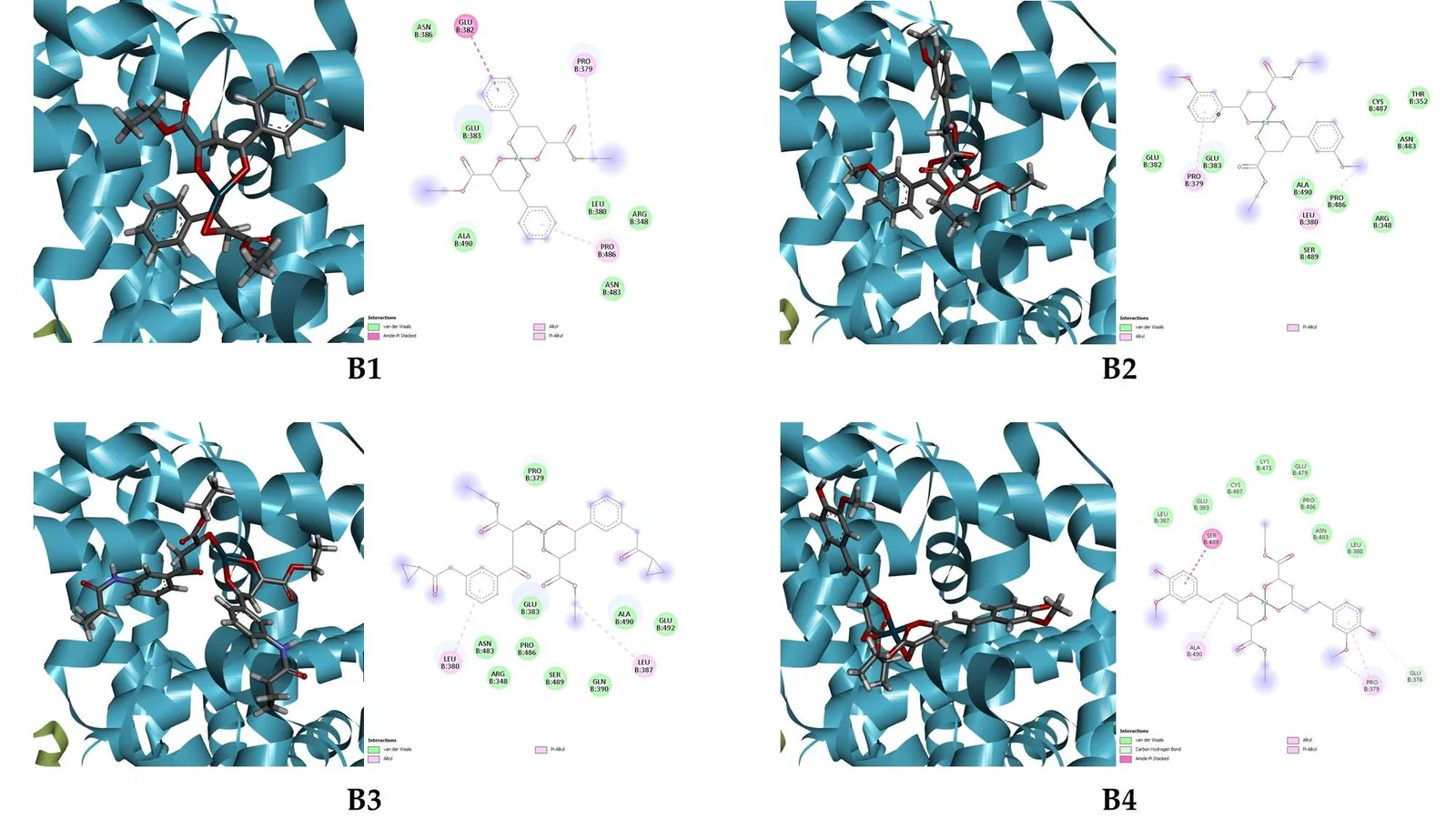
Best docking scenarios and 2D interaction diagram for complexes **B1**–**4** Site II (subdomain IIIA) in HSA (PDB ID: 2BXD).

Complexes **B1** and **B3** demonstrate a high affinity for the classic Sudlow Site II (subdomain IIIA) (Figure 9). Complex **B3** is deeply anchored within this hydrophobic pocket, driven by a dense network of alkyl and π-alkyl interactions with key residues Lys432, Lys436, and Cys448. Conversely, **B1** showcases a more directional binding profile, stabilized by a prominent conventional hydrogen bond with Cys448 and a π-cation interaction with Lys195 at the pocket entrance. A more extended, interface-spanning orientation is observed for **B4**, where the ligand spans across the subdomain IB/IIA/IIIA cleft; here, stability is enhanced by a distinct hydrogen bond with Asn295 and a strong π-cation interaction with Arg218. Finally, **B2** has an alternative binding profile at a distinct interdomain junction involving Domain I and Domain III residues (such as Arg114, Lys190, Glu425, and Lys519), characterized primarily by a π-anion interaction with Glu425 and a surrounding envelope of van der Waals contacts. Overall, these maps indicate that while the complexes are highly compatible with the Sudlow Site II region, their structural bulk allows them to participate in versatile interactions across adjacent interdomain channels.

## 3. Materials and Methods

### 3.1. General

All solvents, substrates, and other reagents were obtained from Sigma Aldrich (St. Louis, MO, USA). Phosphate-buffered saline (PBS) tablets were purchased from Fisher BioReagents (Pittsburgh, PA, USA). Hoechst 33258 (HOE), EB, CT-DNA, eosin Y, and ibuprofen were obtained from Sigma Chemicals Co. (St. Louis, MO, USA), while HSA was purchased from MP Biomedicals LLC (Santa Ana, CA, USA). All stock solutions were prepared using PBS (pH 7.4) to achieve the following final concentrations: 2.61 × 10^−3^ M for CT-DNA, 1.00 × 10^−3^ M for EB, 1.00 × 10^−4^ M for HOE, 2.00 × 10^−5^ M for HSA, and 5.00 × 10^−4^ M for eosin Y and ibuprofen. The purity of the CT-DNA with respect to protein contamination was assessed by measuring the absorbance at 260 and 280 nm. A ratio (A_260_/A_280_) between 1.8 and 1.9 was considered indicative of DNA sufficiently free from protein impurities. The concentration of CT-DNA was determined spectrophotometrically using the absorbance at 260 nm and an extinction coefficient of 6600 M^−1^ cm^−1^. Melting points (uncorrected) were measured using a Mel-Temp apparatus (Cole-Parmer, Vernon Hills, IL, USA). UV-Vis spectra were recorded on a Perkin Elmer Lambda 35 spectrophotometer. IR spectra of the ligands and complexes were obtained with a Perkin–Elmer Spectrum One FT-IR spectrometer (PerkinElmer, Shelton, CT, USA) using KBr pellets. ^1^H and ^13^C NMR spectra were recorded in DMSO-d_6_ with TMS as an internal standard on a Brucker Avance 300 MHz NMR spectrometer (Bruker BioSpin GmbH, Rheinstetten, Germany). Mass spectra were acquired using electrospray ionization (ESI) in positive ion mode on a Waters Micromass Quattro II triple quadrupole mass spectrometer. Instrument control and data processing were performed using MassLynx software (Version 4.0, Waters Corporation, Milford, MA, USA).

### 3.2. General Procedure for the Synthesis of Novel Palladium(II) Complexes ***B1***–***4***

Ligands **A1**–**4** were prepared in our earlier work [26,27,28]. The newly obtained palladium(II) complexes **B1**–**4** were synthesized following a previously reported procedure [29]. In a typical experiment, the corresponding ligand **A1**–**4** (0.2 mmol) was dissolved in 2 mL of methanol, after which an aqueous solution of K_2_[PdCl_4_] (0.1 mmol) was introduced. Within 30 s, the complexes precipitated from the reaction mixture. The reaction progress was monitored by thin-layer chromatography using CH_2_Cl_2_/CH_3_OH (9:1) as the mobile phase. The resulting precipitates were collected by filtration and washed with anhydrous diethyl ether. Recrystallization was carried out from hot methanol, and the products were subsequently isolated by filtration, washed again with diethyl ether, and dried at ambient temperature to afford the final complexes. Structural characterization was performed by NMR spectroscopy, with the ^1^H and ^13^C spectra of complexes **B1**–**4** provided in the ESI (Figures S1–S8).

**B1** [Pd(A1)_2_]. Brown powder; yield: 89%; mp = 157 °C; UV-Vis (λmax/nm, (log(ε/M^−1^ cm^−1^)): 332(4.37); IR (KBr, cm^−1^): ν 3434, 2983, 1728, 1580, 1555, 1520, 1451, 1287, 1238; ^1^H NMR (DMSO-d_6_, 300 MHz) δ 7.85 (d, J = 7.2 Hz, 2H, 2×CH_Ar_), 7.59–7.39 (m, 6H, 2×CH_Ar_), 6.58 (s, 2H, 2×CH=CO), 4.21 (q, J = 7.1 Hz, 4H, 2×OCH_2_), 1.28 (t, J = 7.1 Hz, 6H, 2×CH_3_) ppm; ^13^C NMR (DMSO-d_6_, 75 MHz) δ 184.18, 170.44, 159.85, 134.14, 133.39, 129.13, 127.86, 97.97, 62.53, 14.00 ppm; ESI-MS (*m*/*z*): [M + H]^+^ = 545.2; Anal. Calcd. for C_24_H_22_O_8_Pd (%): C 52.91 H 4.07; Found (%): C 52.98 H 4.08.

**B2** [Pd(A2)_2_]. Brown powder; yield: 82%; mp = 168 °C; UV-Vis (λmax/nm, (log(ε/M^−1^ cm^−1^)): 382(3.64); IR (KBr, cm^−1^): ν 3435, 2986, 1727, 1600, 1556, 1525, 1490, 1430, 1289, 1220; ^1^H NMR (DMSO-d_6_, 300 MHz) δ 7.46–7.28 (m, 6H, 2×CH_Ar_), 7.07 (d, J = 7.0 Hz, 2H, 2×CH_Ar_), 6.52 (s, 2H, 2×CH=CO), 4.21 (q, J = 7.1 Hz, 4H, 2×OCH_2_), 3.75 (s, 6H, 2×OCH_3_), 1.28 (t, J = 7.1 Hz, 6H, 2×CH_3_) ppm; ^13^C NMR (DMSO-d_6_, 75 MHz) δ 187.72, 173.38, 165.65, 159.49, 141.27, 129.85, 119.46, 117.44, 112.15, 93.91, 61.23, 55.29, 14.26 ppm; ESI-MS (*m*/*z*): [M + H]^+^ = 605.3; Anal. Calcd. for C_26_H_26_O_10_Pd (%): C 51.63 H 4.33; Found (%): C 51.68 H 4.35.

**B3** [Pd(A3)_2_]. Brown powder; yield: 77%; mp = 198 °C; UV-Vis (λmax/nm, (log(ε/M^−1^ cm^−1^)): 383(4.27); IR (KBr, cm^−1^): ν 3490, 3437, 2988, 1727, 1568, 1508, 1282, 1218; ^1^H NMR (DMSO-d_6_, 300 MHz) δ 10.48 (s, 2H, 2×NH), 8.29 (s, 2H, 2×CH_Ar_), 7.91 (d, J = 7.8 Hz, 2H, 2×CH_Ar_), 7.72 (d, J = 7.6 Hz, 2H, 2×CH_Ar_), 7.49 (t, J = 7.9 Hz, 2H, 2×CH_Ar_), 7.02 (s, 2H, 2×CH=CO), 4.31 (q, J = 7.0 Hz, 4H, 2×OCH_2_), 1.83–1.74 (m, 2H, 2×CH*c_Pr_*), 1.31 (t, J = 7.1 Hz, 6H, 2×CH_3_), 0.85–0.79 (m, 8H, 2×CH_2_*_cPr_*) ppm; ^13^C NMR (DMSO-d_6_, 75 MHz) δ 190.21, 172.29, 169.09, 161.76, 140.29, 135.06, 129.87, 124.45, 122.64, 117.83, 98.10, 62.47, 14.85, 14.07, 7.63 ppm; ESI-MS (*m*/*z*): [M + H]^+^ = 711.0; Anal. Calcd. for C_32_H_32_N_2_O_10_Pd (%): C 54.06 H 4.54 N 3.94; Found (%): C 54.11 H 4.55 N 3.93.

**B4** [Pd(A4)_2_]. Brown powder; yield: 77%; mp = 203 °C; UV-Vis (λmax/nm, (log(ε/M^−1^ cm^−1^)): 349(4.05);IR (KBr, cm^−1^): ν 3458, 2984, 1731, 1668, 1558, 1486, 1407, 1303, 1223; ^1^H NMR (DMSO-d_6_, 300 MHz) δ 9.86 (s, 2H, 2×OH), 7.71 (d, J = 15.8 Hz, 2H, 2×CH=), 7.39 (s, 2H, 2×CH_Ar_), 7.21 (d, J = 7.7 Hz, 2H, 2×CH_Ar_), 7.01–6.81 (m, 4H, 2×CH_Ar_ + 2×CH=), 6.58 (s, s, 2H, 2×CH=CO), 4.28 (q, J = 7.1 Hz, 4H, 2×OCH_2_), 3.83 (s, 6H, 2×OCH_3_), 1.29 (t, J = 7.1 Hz, 6H, 2×CH_3_) ppm; ^13^C NMR (DMSO-d_6_, 75 MHz) δ 186.38, 172.41, 161.93, 150.47, 148.27, 144.38, 126.19, 124.59, 120.43, 115.91, 111.64, 100.96, 62.20, 55.87, 14.12 ppm; ESI-MS (*m*/*z*): [M + H]^+^ = 689.0; Anal. Calcd. for C_30_H_30_O_12_Pd (%): C 52.30 H 4.39; Found (%): C 52.33 H 4.40.

### 3.3. Stability of Palladium(II) Complexes ***B1***–***4***

The stability of complexes **B1**–**4** in aqueous physiological conditions was assessed using UV–Vis spectrophotometric measurements. Stock solutions of each palladium(II) complex were freshly prepared in DMSO at a concentration of 1 × 10^−3^ M and subsequently diluted with phosphate-buffered saline (PBS, pH 7.4) to a final concentration of 1.35 × 10^−5^ M. The corresponding PBS/DMSO medium, without the tested complex, was employed as the reference solution. Spectra were recorded at room temperature under the experimental conditions described above.

### 3.4. Antimicrobial Activity

The antimicrobial activity of palladium(II) complexes **B1**–**4** was evaluated against nine laboratory control strains of microorganisms. Overall, the complexes exhibited limited selectivity towards malignant laboratory control strains of microorganisms. The panel comprised four Gram-positive bacteria: microaerophilic *Staphylococcus aureus* subsp. *aureus* Rosenbach ATCC 6538, *Staphylococcus epidermidis* ATCC 12228, *Enterococcus faecalis* ATCC 29212, and the anaerobe *Cutibacterium acnes* ATCC 29399; four Gram-negative bacteria: *Escherichia coli* ATCC 25922, *Klebsiella pneumoniae* subsp. *pneumoniae* NCIMB 8267, *Salmonella enterica* subsp. *enterica* serovar Abony NCTC 6017, and *Pseudomonas aeruginosa* ATCC 27853; and one yeast, *Candida albicans* ATCC 24433.

Lyophilised pellets were rehydrated prior to testing. Bacterial strains were inoculated onto Tryptic Soy agar/broth (TSA/TSB, Oxoid Ltd., Basingstoke, Hampshire, UK) and Mueller–Hinton broth (MHB, Oxoid); *C. acnes* was cultivated on blood agar (anaerobe agar supplemented with 5% defibrinated horse blood) and in thioglycolate broth (TG, Merck-Millipore, Darmstadt, Germany), while *C. albicans* was grown on Sabouraud Dextrose agar/broth (SDA/SDB, Oxoid). Staphylococci and *C. albicans* were incubated aerobically for 24–48 h at 35 ± 1 °C, whereas *C. acnes* were incubated anaerobically (GasPack^®^ systems, Becton, Dickinson and Company, Sparks, MD, USA) for 48–72 h at 35–37 °C.

MICs were determined using the broth microdilution method in flat-bottomed 96-well microtiter plates, following the European Committee for Antimicrobial Susceptibility Testing (EUCAST 2026) [90,91] guidelines. Inocula were prepared from fresh cultures suspended in saline to a turbidity equivalent to 0.5 McFarland (BioMerieux, France). Test compounds **B1**–**4** were dissolved in DMSO and diluted in fresh MHB, TG, or SDB to final concentrations ranging from 5–5000 μM. Each concentration was tested in triplicate and inoculated with 5 × 10^5^ CFU/mL of microorganisms.

To monitor microbial growth and metabolic activity, broths were supplemented with the redox indicator resazurin (7-Hydroxy-3H-phenoxazin-3-one 10-oxide) (Sigma-Aldrich, St. Louis, MO, USA). This blue, non-fluorescent dye is reduced to the pink metabolite resorufin (7-Hydroxy-3H-phenoxazin-3-one) in the presence of viable cells. MIC values were recorded after 20–48 h of incubation at 35 °C under aerobic or anaerobic conditions, depending on the microorganism, and defined as the lowest concentration of compounds preventing visible growth. Positive controls (microorganisms in medium) and negative controls (medium with test samples only) were included. All experiments were performed in triplicate.

### 3.5. Cytotoxicity Studies

The cytotoxic activity of the palladium(II) complexes was evaluated against five human cell lines: MRC-5, HaCaT, HeLa, K-562 and DU-145. The cell lines were obtained from the American Type Culture Collection (ATCC, Manassas, VA, USA): MRC-5 human fetal lung fibroblasts (ATCC^®^ CCL-171™), HeLa human cervical adenocarcinoma cells (ATCC^®^ CCL-2™), DU145 human prostate carcinoma cells (ATCC^®^ HTB-81™), and K-562 human chronic myeloid leukaemia cells (ATCC^®^ CCL-243™). The human skin keratinocyte cell line HaCaT (300493) was purchased from Cytion/CLS Cell Lines Service (Heidelberg, Germany).

All cell lines were cultured in RPMI-1640 medium supplemented with 10% foetal bovine serum (FBS), 100 U mL^−1^ penicillin, and 100 μg mL^−1^ streptomycin under standard culture conditions (37 °C, 5% CO_2_, humidified atmosphere).

Cell viability was determined using the 3-(4,5-dimethylthiazol-2-yl)-2,5-diphenyltetrazolium bromide (MTT) assay according to the method of [92], with minor modifications. Briefly, adherent cells were seeded into 96-well plates at an appropriate density (typically 5 × 10^3^–1 × 10^4^ cells per well), while K-562 suspension cells were seeded at 1 × 10^4^–2 × 10^4^ cells per well. After a 24 h incubation to allow cell attachment (for adherent cells), the cells were treated with serial concentrations of the palladium(II) complexes (12.5–200 μM) and incubated for 72 h. Untreated cells served as the negative control.

Following incubation, 20 μL of MTT solution (5 mg mL^−1^ in phosphate-buffered saline) was added to each well, and the plates were incubated for a further 4 h at 37 °C to allow the formation of formazan crystals by metabolically active cells. Sodium dodecyl sulphate (SDS) solubilisation solution was then added to dissolve the formazan crystals, and the plates were left until complete dissolution, yielding a homogeneous brown solution. Absorbance was measured at 570 nm using a microplate reader, with a reference wavelength of 630–690 nm.

Cell viability was expressed as a percentage relative to untreated control cells according to the following equation:

Cell viability (%) = (A_treated_/A_control_) × 100

where A_treated_ and A_control_ represent the absorbance of treated and untreated cells, respectively.

Cell viability data were normalized to the corresponding untreated control and expressed as percentage viability. Concentration–response relationships were analysed separately for each compound and cell line using non-linear regression in GraphPad Prism 9.0. IC_50_ values were estimated by fitting a logistic concentration–response model with variable slope to the normalized viability data. Goodness of fit was assessed by the coefficient of determination (R^2^), and 95% confidence intervals were calculated for the fitted IC_50_ estimates when the parameter was adequately defined by the experimental concentration range. When 50% inhibition was not reached at the highest tested concentration (200 μM), the IC_50_ was reported as >200 μM rather than extrapolated beyond the experimentally tested range. For statistical comparisons, cell lines were analysed separately because they represent biologically distinct cellular models. Comparisons among **B1**–**4** were therefore performed within each cell line rather than by pooling IC_50_ values across different cell lines.

The selectivity of the palladium(II) complexes towards malignant cells was assessed by calculating the SI, defined as the ratio of the IC_50_ value obtained for normal cells to that obtained for cancer cells. The SI is calculated as [93]

SI = IC_50_ (normal cells)/IC_50_ (cancer cells)

SI values were calculated separately using MRC-5 human foetal lung fibroblasts and HaCaT human keratinocytes as normal cell models. An SI value greater than 1 indicates preferential cytotoxicity towards malignant cells relative to normal cells, whereas SI values below 1 indicate little or no selectivity and suggest similar or greater toxicity towards normal cells. A selectivity index of ≥2 is often considered indicative of promising selective anticancer activity.

### 3.6. The Effect of Palladium(II) Complexes on the Cell-Cycle of HeLa Cells

Cell-cycle distribution was determined by PI staining followed by flow cytometric analysis, as previously described by Sova et al. [94]. Briefly, following 48 h treatment, 0.5 × 10^6^ cells per sample were harvested, washed with PBS, and fixed in 70% ethanol. After overnight incubation at 4 °C, the cells were washed with PBS and resuspended in 500 µL of staining buffer (100 mM Tris, pH 7.4, 150 mM NaCl, 1 mM CaCl_2_, 0.5 mM MgCl_2_, and 0.1% Nonidet P-40) containing 3 µM PI and 100 µg/mL RNase A. Samples were incubated at 37 °C for 30 min and analyzed using a FACSCalibur flow cytometer (Becton Dickinson, San Jose, CA, USA). Three independent experiments were performed, each in triplicate.

### 3.7. Statistical Analysis

Statistical analysis was performed with the statistical software GraphPad Prism version 9.0 for Windows, GraphPad Software, Boston, MA, USA, (www.graphpad.com) using the methods of descriptive statistics, one-way ANOVA with Tukey’s post hoc test, and non-linear regression, Friedman test (non-parametric repeated-measures ANOVA) with Dunn’s post hoc test. The data are presented as the mean value ± SD. The significance was accepted at a *p* value < 0.05.

### 3.8. Lipophilicity Assay

The lipophilicity parameters of ligands **A1**–**4** were evaluated employing the standard shake-flask technique [74]. Initially, stock solutions of the examined compounds prepared in methanol were introduced into an n-octanol/water biphasic system. The mixtures were thoroughly vortexed at room temperature for 1 h to ensure complete equilibration and subsequently left undisturbed for 24 h to achieve distinct phase separation. The concentrations of the target ligands in both the organic and aqueous layers were quantified spectrophotometrically by measuring their absorbance against pre-established calibration curves. Finally, the partition coefficients (*log P*) were determined using a well-established equation [76].

### 3.9. DNA–Absorption Binding Studies

UV-Vis absorption spectroscopy was employed to investigate the potential interaction between CT-DNA and four newly synthesized palladium(II) complexes. Solutions of the complexes were prepared at a fixed concentration of 1.35 × 10^−5^ M, and titrated with increasing concentrations of CT-DNA ranging from 1.35 × 10^−5^ M to 1.35 × 10^−4^ M. The experiments were conducted at room temperature following a 24 h incubation period to ensure equilibrium. Absorption spectra were recorded in the range of 200–600 nm.

The *K*_b_ constant for each complex was calculated using the Wolfe–Shimmer equation [13], based on the changes observed in the UV-Vis spectra upon incremental addition of CT-DNA.

### 3.10. Competitive Fluorescence Measurements

Fluorescence emission spectroscopy was employed to evaluate the potential of the investigated palladium(II) complexes to displace EB from the CT-DNA-EB complex, thereby indicating intercalative binding. A fixed concentration of CT-DNA and EB (1.35 × 10^−5^ M each) was titrated with increasing concentrations of the palladium(II) complexes, ranging from 1.33 × 10^−5^ M to 1.19 × 10^−4^ M. After a 5 min incubation period at room temperature, fluorescence spectra were recorded using an excitation wavelength of 527 nm and an emission range of 550–750 nm. Control measurements confirmed that the investigated complexes alone, recorded under identical experimental conditions, exhibited no intrinsic fluorescence within the monitored spectral region, thereby ruling out any potential spectral interference.

To assess the quenching efficiency and binding affinity of the complexes in the presence of EB, the *K*_SV_ constant was determined using the Stern–Volmer equation [13].

### 3.11. Viscosity Measurements

Viscosity measurements were performed using a rolling-ball viscosimeter (Lovis 2000 M/ME, Anton Paar GmbH, Graz, Austria) to investigate the mode of interaction between CT-DNA and the **B2** complex. A series of solutions were prepared by keeping the concentration of CT-DNA constant at 1.35 × 10^−5^ M, while varying the concentration of the **B2** complex in the range of 1.35 × 10^−5^ M to 8.10 × 10^−5^ M. Measurements were conducted at a constant temperature of 298 K.

The viscosity of the CT-DNA solution without the complex was denoted as η_0_, while η represents the viscosity of the DNA solution in the presence of **B2**. To analyze the binding behavior, the relative specific viscosity, expressed as (η/η_0_)^1/3^, was plotted against the molar ratio [complex]/[DNA].

### 3.12. Protein Binding Studies

The interactions of the palladium(II) complexes **B1**–**4** with HSA were investigated by fluorescence spectroscopy, both in the absence and presence of specific site markers—eosin Y and ibuprofen—used to probe binding at Sudlow’s sites I and II, respectively. For all experiments, HSA and either eosin Y or ibuprofen were maintained at a constant concentration of 2.00 × 10^−6^ M, while the concentration of the Pd(II) complexes gradually increased in the range of 2.49 × 10^−7^ M to 2.44 × 10^−6^ M.

Fluorescence emission spectra were recorded using an excitation wavelength of 295 nm, with emission monitored in the range of 300–500 nm. Quenching data were analyzed using the Stern–Volmer equation [13] to determine the quenching mechanism and binding affinity. Additionally, the Hill equation [13] was applied to estimate the binding constants and the number of binding sites involved in the complex–HSA interaction.

### 3.13. Quantum-Chemical Methods

The structures of the complexes were optimized at the B3LYP/def2-SVP [95,96,97,98] level of theory using Gaussian09 program [99]. To confirm that the optimized structures correspond to true minima on the potential energy surface, harmonic vibrational frequency calculations were performed. All molecular structures were fully optimized both in the gas phase and in the aqueous phase using the Density-Based Solvation Model, followed by vibrational frequency calculations at the same level of theory to confirm the nature of the stationary points. Restrained electrostatic potential (RESP) charges were subsequently calculated using the same software [99]. The MPP and SDP are calculated as the root-mean-square deviation and the total range (maximum minus minimum distance) of the coordinated atoms from the least-squares mean plane, respectively [100]. The BLA is determined by computing the absolute differences between alternating or adjacent coordination bond lengths within the metal’s primary coordination sphere [30].

### 3.14. Molecular Docking Simulations

The optimized structures of the investigated complexes were docked into structural fragments of DNA and HSA macromolecules. Initial coordinates for a canonical B-DNA fragment (PDB ID: 1BNA), DNA with an intercalation gap (PDB ID: 1Z3F), and human serum albumin (PDB ID: 2BXD) were retrieved from the Protein Data Bank (http://www.rcsb.org). Prior to docking, all water molecules, existing ligands, and heteroatoms were removed. Molecular docking simulations were carried out using AutoDock Vina, (version 1.1.2) [101] utilizing the standard Vina scoring function. For all docking protocols, target structures were treated as rigid receptors. As described in the Methodology section, Restrained Electrostatic Potential (RESP) charges were subsequently calculated using the same software [99] and assigned to the palladium(II) complexes. During ligand preparation, the coordination core was set as a non-rotatable/rigid framework derived directly from the DFT-optimized structure, while all rotatable single bonds in the organic ligands were kept flexible. The docking parameters were configured with an exhaustiveness of 25, an energy range of 5 kcal mol^−1^, and a maximum output of 25 binding poses. The search spaces were defined using grid boxes centered on the respective binding sites for each target structure (DNA and HSA Sudlow Sites I and II). To ensure full reproducibility, the exact Cartesian coordinates of the grid box centers and grid dimensions for all targets are provided in Table S3 in the ESI. Binding affinities were evaluated in kcal mol^−1^, and the generated poses were ranked to assess structural variations relative to the top mode using Root-Mean-Square Deviation (RMSD) values [31]. Finally, the docked conformations were visualized using BIOVIA Discovery Studio 2025 [102].

## 4. Conclusions

In this study, we synthesized and evaluated four novel palladium(II) complexes (**B1**–**4**) bearing *O*,*O*-bidentate β-diketo ester ligands, establishing a clear structure–activity relationship (SAR) that links their physicochemical properties to their biological efficacy. Spectroscopic and quantum chemical analyses confirmed that the specific substituents on the β-diketonate scaffold dictate the degree of structural distortion from the ideal square-planar geometry. Crucially, the lipophilicity and structural planarity of the complexes emerged as the primary drivers of their biological behavior. In the antimicrobial evaluation, within the present series, **B3** and **B4** generally exhibited lower MIC values against several of the tested microorganisms than **B1** and **B2**.

Regarding antiproliferative activity, the investigated complexes exhibited distinct potency and selectivity profiles. Complex **B3** showed the highest antiproliferative potency among the investigated compounds, with IC_50_ values of 2.3, 5.2, and 61.4 μM against K-562, HeLa, and DU-145 cells, respectively. However, its selectivity was dependent on the cell model. B3 showed its most favorable selectivity towards K-562 cells, with SI values of 5.17 and 2.35 relative to MRC-5 and HaCaT cells, respectively. In contrast, its selectivity towards HeLa cells was more limited, particularly when HaCaT cells were used as the reference (SI = 1.04). Complex **B2** also showed moderate selectivity towards K-562 cells, whereas the selectivity of the other complexes was generally limited. The comparatively high sensitivity of HaCaT cells to **B3** further emphasizes the importance of evaluating both antiproliferative potency and selectivity when considering the potential of these compounds for further development.

Mechanistic investigations revealed that these Pd(II) complexes exert their anticancer effects primarily through the induction of cell-cycle-independent apoptosis. This is largely attributed to the soft Lewis acid nature of the Pd(II) center, which exhibits a high affinity for sulfur-containing biomolecules, thereby disrupting intracellular redox homeostasis, interacting with thiol-containing enzymes, and ultimately triggering apoptotic pathways without causing pronounced phase-specific cell-cycle arrest.

Biophysical and computational assays further elucidated the interaction of these complexes with key biological macromolecules, highlighting how structural features dictate binding modes. The high degree of structural planarity, particularly in the **B4** complex, facilitates strong, non-intercalative minor groove binding to DNA, as confirmed by molecular docking and viscosity measurements. Furthermore, all complexes demonstrated a high affinity for human serum albumin, with molecular docking revealing a thermodynamically favorable accommodation within the Sudlow binding sites. This strong protein-binding capacity suggests that these complexes possess favorable systemic transport and bioavailability profiles, which is critical for their potential in vivo pharmacokinetics.

Overall, this work highlights the potential of Pd(II) β-diketo ester complexes as bioactive compounds and underscores the importance of ligand structure in modulating their biological properties. Among the investigated compounds, **B3** exhibited the highest antiproliferative potency and the most favorable selectivity towards K-562 cells. Future studies should focus on elucidating the molecular mechanisms underlying the biological activity of **B3**, further evaluating its selectivity across a broader range of cancer and non-malignant cell models, and assessing its behavior under biologically relevant conditions. Further structural optimization may help identify ligand modifications that improve the balance between antiproliferative potency and cellular selectivity, particularly in less sensitive cancer cell models such as DU-145.

## Figures and Tables

**Table 1:** Antimicrobial activity of complexes **B1**–**4**.

	Compound (MIC μM)			
Microorganism	B1	B2	B3	B4
*Staphylococcus aureus*	625	625	312	312
*Staphylococcus epidermidis*	312	312	312	312
*Enterococcus faecalis*	312	312	312	312
*Escherichia coli*	800	1200	400	400
*Klebsiella pneumoniae*	800	1200	400	400
*Salmonella abony*	800	1200	800	400
*Pseudomonas aeruginosa*	800	1200	1200	800
*Candida albicans*	625	312	312	312
*Cutibacterium acnes*	625	312	625	312

**Table 2:** Effect of complexes **B1**–**4** on cell viability.

Cell Lines	Palladium(II) Complexes (μM)			
	B1	B2	B3	B4
MRC-5				
IC_50_	61.6	40.8	11.9	25.6
95% CI	0–148.6	27.7–54.0	1.3–22.5	4.9–46.2
HaCaT				
IC_50_	>200 *	57.6	5.4	60.8
95% CI	n.a.	6.1–109.2	4.3–6.5	21.9–99.7
HeLa				
IC_50_	139.2	42.2	5.2	64
95% CI	85.3–193.2	14.9–69.5	0–12.1	34.7–93.3
K-562				
IC_50_	165.1	26.6	2.3	39.2
95% CI	128.8–201.5	22.2–31.1	0–6.6	23.2–55.2
DU-145				
IC_50_	>200 *	120.3	61.4	138.4
95% CI	n.a.	4.4–236.2	17.7–105.1	62.6–214.2

* For HaCaT and DU-145 cell lines, 50% inhibition was not reached within the tested concentration range of **B1** (12.5–200 µM); IC_50_ values are reported as >200 µM.

**Table 3:** The selectivity indices (SI) of **B1**–**4** complexes based on MRC-5 and HaCaT cell line.

Cancer Cell Line	Selectivity Index (SI)							
	SI Based on MRC-5				SI Based on HaCaT			
	B1	B2	B3	B4	B1	B2	B3	B4
HeLa	0.44	0.97	2.29	0.40	>1.44	1.36	1.04	0.95
K-562	0.37	1.53	5.17	0.65	>1.21	2.17	2.35	1.55
DU-145	<0.32	0.34	0.19	0.18	ND *	0.48	0.09	0.44

* Not determined, as both IC_50_ values exceed 200 μM.

**Table 4:** DNA-binding constants (*K*_b_) and Stern–Volmer constants (*K*_sv_) obtained from EB-DNA.

	*K*_b_ × 10^3^ (M^−^^1^)	*K_sv_* (*EB*) × 10^4^ (M^−1^)
**B1**	1.38 ± 0.02	5.21 ± 0.06
**B2**	2.14 ± 0.04	16.20 ± 0.01
**B3**	2.78 ± 0.02	1.34 ± 0.02
**B4**	2.16 ± 0.07	4.75 ± 0.04

**Table 5:** Binding parameters for complexes **B1**–**4** with HSA (without and with site markers).

		HSA	HSA-Eosin Y	HSA-Ibuprofen
**B1**	K_sv_ (M^−1^)	(7.27 ± 0.06) × 10^5^	(1.03 ± 0.08) × 10^6^	(5.46 ± 0.02) × 10^5^
	k_q_ (M^−1^ s^−1^)	(7.27 ± 0.03) × 10^13^	(1.03 ± 0.03) × 10^14^	(5.46 ± 0.01) × 10^13^
	K (M^−1^)	(1.43 ± 0.01) × 10^6^	(1.10 ± 0.01) × 10^4^	(1.69 ± 0.03) × 10^6^
	*n*	1.05	0.64	1.10
**B2**	K_sv_ (M^−1^)	(1.86 ± 0.01) × 10^6^	(4.79 ± 0.03) × 10^5^	(3.44 ± 0.03) × 10^5^
	k_q_ (M^−1^ s^−1^)	(1.86 ± 0.04) × 10^14^	(4.79 ± 0.01) × 10^13^	(3.44 ± 0.01) × 10^13^
	K (M^−1^)	(1.65 ± 0.03) × 10^8^	(1.63 ± 0.02) × 10^5^	(1.85 ± 0.03) × 10^5^
	*n*	1.37	1.30	0.96
**B3**	K_sv_ (M^−1^)	(2.44 ± 0.06) × 10^5^	(2.14 ± 0.07) × 10^5^	(2.12 ± 0.07) × 10^5^
	k_q_ (M^−1^ s^−1^)	(2.44 ± 0.05) × 10^13^	(2.14 ± 0.04) × 10^13^	(2.12 ± 0.07) × 10^13^
	K (M^−1^)	(1.98 ± 0.03) × 10^4^	(2.64 ± 0.02) × 10^4^	(6.24 ± 0.02) × 10^4^
	*n*	0.80	0.84	0.90
**B4**	K_sv_ (M^−1^)	(3.15 ± 0.01) × 10^5^	(7.03 ± 0.01) × 10^5^	(3.80 ± 0.02) × 10^5^
	k_q_ (M^−1^ s^−1^)	(3.15 ± 0.08) × 10^13^	(7.03 ± 0.03) × 10^13^	(3.80 ± 0.01) × 10^13^
	K (M^−1^)	(5.37 ± 0.01) × 10^4^	(3.36 ± 0.02) × 10^2^	(2.95 ± 0.04) × 10^8^
	*n*	0.86	0.38	1.52

**Table 6:** Calculated values for BLA (in Å) for Pd-O distances, MPP (in Å) and SDP (in Å) for distorted square-planar structure in studied molecules.

	BLA	MPP	SDP
**B1**	0.729	0.741	2.049
**B2**	0.151	0.749	1.767
**B3**	0.525	0.854	2.055
**B4**	0.090	0.601	1.390

**Table 7:** Docking score values (in kcal mol^−1^) for DNA and HSA protein with investigated complexes.

PDB ID of Macromolecule	Complex	Docking Score Values
1BNA	**B1**	−10.9
	**B2**	−10.7
	**B3**	−12.2
	**B4**	−12.9
2BXD (Site I, subdomain IIA)	**Eosin**	−10.5
	**B1**	−14.9
	**B2**	−13.2
	**B3**	−15.7
	**B4**	−15.3
2BXD (Site II, subdomain IIIA)	**Ibuprofen**	−9.2
	**B1**	−10.4
	**B2**	−10.1
	**B3**	−11.3
	**B4**	−10.9

## Data Availability

The original contributions presented in this study are included in the article/Supplementary Materials. Further inquiries can be directed to the corresponding authors.

## Supplementary Materials

The following supporting information can be downloaded at https://www.mdpi.com/article/10.3390/ijms27219437/s1.

## Abbreviations

The following abbreviations are used in this manuscript:

ABC	ATP-binding cassette
BLA	Bond length alternantion
CT-DNA	Calf thymus DNA
DFT	Density functional theory
DU-145	Human prostate carcinoma
EB	Ethidium bromide
ESI	Electrospray ionization
EUCAST	European Committee for Antimicrobial Susceptibility Testing
FBS	Foetal bovine serum
HaCaT	Immortalised normal human keratinocytes
HeLa	Human cervical carcinoma
HSA	Human serum albumin
IC_50_	Concentrations causing 50% inhibition of cell viability
IR	Infrared
K_b_	Intrinsic binding constant
K-562	Human chronic myelogenous leukaemia
K_SV_	Stern–Volmer quenching constant
kq	Bimolecular quenching constants
MICs	Minimum inhibitory concentrations
MPP	Molecular planarity parameter
MRC-5	Normal human foetal lung fibroblasts
MTT	3-(4,5-dimethylthiazol-2-yl)-2,5-diphenyltetrazolium bromide
PBS	Phosphate-buffered saline
PDB ID:1BNA	Canonical B-DNA fragment
PDB ID:1Z3F	DNA with an intercalation gap
PI	Propidium iodide
RMSD	Root-Mean-Square Deviation
ROS	Reactive oxygen species
SD	Standard deviation
SDP	Span of deviation from the plane
SDS	Sodium dodecyl sulfate
SI	Selectivity index
UV-Vis	Electronic absorption spectroscopy

## References

[1] Kenny R.G., Marmion C.J. (2019). Toward Multi-Targeted Platinum and Ruthenium Drugs—A New Paradigm in Cancer Drug Treatment Regimens?. Chem. Rev..

[2] Al-Rashdi K.S., Babgi B.A., Ali E.M.M., Davaasuren B., Emwas A.H.M., Jaremko M., Humphrey M.G., Hussien M.A. (2024). Dna-Binding and Antiproliferative Properties of Palladium (II) Complexes with Tridentate Ligands. Inorganica Chim. Acta.

[3] Radisavljević S., Ćoćić D., Petrović B., Kellner I., Ivanović-Burmazović I., Radenković N., Nikodijević D., Milutinović M. (2024). New Dinuclear Gold(III) Complex with 1,5-Naphthyridine as Bridging Ligand: Synthesis, Characterization, DNA/BSA Binding Studies, and Anticancer Activity. Gold Bull..

[4] Shruthi S., Bhasker Shenoy K. (2024). Cisplatin Resistance in Cancer Therapy: Causes and Overcoming Strategies. ChemistrySelect.

[5] Bogojeski J., Soldatović T., Petrović B. (2024). Mechanisms of the Reactions of Structurally Similar Platinum(II), Palladium(II) and Gold(III) Complexes: A Minireview Dedicated to the Research of Prof. Dr. Živadin D. Bugarčić. J. Coord. Chem..

[6] Carneiro T.J., Martins A.S., Marques M.P.M., Gil A.M. (2020). Metabolic Aspects of Palladium(II) Potential Anti-Cancer Drugs. Front. Oncol..

[7] Kuijpers T., Blom B. (2021). Homo and Heterobimetallic Palladium and Platinum Complexes Bearing μ-Diphosphane Bridges Involved in Biological Studies. Eur. J. Med. Chem..

[8] Joksimović N., Janković N., Davidović G., Bugarčić Z. (2020). 2,4-Diketo Esters: Crucial Intermediates for Drug Discovery. Bioorg. Chem..

[9] Janković N., Bogdanović G.A., Gligorijević N., Milović L., Međedović M., Matić J., Kosanić M., Vraneš M., Simović A.R. (2025). Novel Organoruthenium Complexes Containing β-Diketonates: Synthesis, Characterization, DNA/HSA Interactions, and the Impact of Biocompatible Ionic Liquids on Biological Activities. J. Inorg. Biochem..

[10] Saygıdeğer Demir B., İnce S., Yilmaz M.K., Sezan A., Derinöz E., Taskin-Tok T., Saygideger Y. (2022). DNA Binding and Anticancer Properties of New Pd(II)-Phosphorus Schiff Base Metal Complexes. Pharmaceutics.

[11] Radisavljević S., Kesić A., Ćoćić D., Marković V., Milovanović J., Petrović B., Simović A.R. (2023). New Gold(III) Chlorophenyl Terpyridine Complex: Biomolecular Interactions and Anticancer Activity against Human Oral Squamous Cell Carcinoma. Appl. Organomet. Chem..

[12] Radisavljević S., Scheurer A., Bockfeld D., Ćoćić D., Puchta R., Senft L., Pešić M., Damljanović I., Petrović B. (2021). New Mononuclear Gold(III) Complexes: Synthesis, Characterization, Kinetic, Mechanistic, DNA/BSA/HSA Binding, DFT and Molecular Docking Studies. Polyhedron.

[13] Piskulić K., Stojanović S., Petronijević J., Filipović I., Janković N., Muškinja J., Bogdanović G.A., Matić I.Z., Stanojković T., Branković S. (2025). Synthesis, Characterization, and Anticancer Potential of Some 2,4-Diketo Esters Containing Dehydrozingerone Fragment: DNA and Protein Binding Study and Molecular Docking. J. Mol. Struct..

[14] Bai G.Y., Wang K.Z., Duan Z.M., Gao L.H. (2004). Luminescent PH Sensing and DNA Binding Properties of a Novel Ruthenium(II) Complex. J. Inorg. Biochem..

[15] Wang Q., Yang Z.Y., Qi G.F., Qin D.D. (2009). Synthesis, Crystal Structure, Antioxidant Activities and DNA-Binding Studies of the Ln(III) Complexes with 7-Methoxychromone-3-Carbaldehyde-(4′-Hydroxy) Benzoyl Hydrazone. Eur. J. Med. Chem..

[16] Tarushi A., Lafazanis K., Kljun J., Turel I., Pantazaki A.A., Psomas G., Kessissoglou D.P. (2013). First- and Second-Generation Quinolone Antibacterial Drugs Interacting with Zinc(II): Structure and Biological Perspectives. J. Inorg. Biochem..

[17] Pugachev A.D., Kozlenko A.S., Makarova N.I., Rostovtseva I.A., Ozhogin I.V., Dmitriev V.S., Borodkin G.S., Tkachev V.V., Utenyshev A.N., Sazykina M.A. (2023). Molecular Design and Synthesis of Methoxy-Substitued Spiropyrans with Photomodulated NIR-Fluorescence. Photochem. Photobiol. Sci..

[18] Pugachev A.D., Kozlenko A.S., Sazykina M.A., Sazykin I.S., Rostovtseva I.A., Makarova N.I., Borodkin G.S., Tkachev V.V., Utenyshev A.N., Demidov O.P. (2025). Anion and Substituents Effect on Spectral-Kinetic and Biological Characteristics of Spiropyran Salts. ChemBioChem.

[19] Pronkin P.G., Tatikolov A.S. (2022). Meso-Aryl-Substituted Thiacarbocyanine Dyes as Spectral-Fluorescent Probes for DNA. Spectrochim. Acta A Mol. Biomol. Spectrosc..

[20] Pugachev A.D., Bardasov I.N., Karchava S.K., Azhogina T.N., Klimova M.V., Matukhno A.E., Dmitriev V.S., Borodkin G.S., Lanovaya O.D., Pobedinskaya D.Y. (2025). New Benzimidazole-Based PH-Sensitive Fluorescent Probes. Molecules.

[21] Perendija S., Dimić D., Eichhorn T., Rakić A., Saso L., Nakarada Đ., Đikić D., Dragojević T., Dimitrić Marković J., Kaluđerović G.N. (2025). Synthesis, Characterization, HSA/DNA Binding, and Cytotoxic Activity of [RuCl2(H6-p-Cymene)(Bph-ΚN)] Complex. Molecules.

[22] Shinziya H., Menon R.S., Das A.K. (2024). A Rapid Investigation of Near-Infrared (NIR) Fluorescent Switch-on Probes for Detection and in Cellulo Tracking of G-Quadruplex and Double-Stranded DNA. RSC Adv..

[23] Sudlow G., Birkett D.J., Wade D.N. (1975). The Characterization of Two Specific Drug Binding Sites on Human Serum Albumin. Mol. Pharmacol..

[24] Ghuman J., Zunszain P.A., Petitpas I., Bhattacharya A.A., Otagiri M., Curry S. (2005). Structural Basis of the Drug-Binding Specificity of Human Serum Albumin. J. Mol. Biol..

[25] Yuan J.L., lv Z., Liu Z.G., Hu Z., Zou G.L. (2007). Study on Interaction between Apigenin and Human Serum Albumin by Spectroscopy and Molecular Modeling. J. Photochem. Photobiol. A Chem..

[26] Joksimović N., Baskić D., Popović S., Zarić M., Kosanić M., Ranković B., Stanojković T., Novaković S.B., Davidović G., Bugarčić Z. (2016). Synthesis, Characterization, Biological Activity, DNA and BSA Binding Study: Novel Copper(II) Complexes with 2-Hydroxy-4-Aryl-4-Oxo-2-Butenoate. Dalton Trans..

[27] Joksimović N., Petronijević J., Janković N., Kosanić M., Milivojević D., Vraneš M., Tot A., Bugarčić Z. (2021). Synthesis, Characterization, Antioxidant Activity of β-Diketonates, and Effects of Coordination to Copper (II) Ion on Their Activity: DNA, BSA Interactions and Molecular Docking Study. Med. Chem..

[28] Petronijević J., Bugarčić Z., Bogdanović G.A., Stefanović S., Janković N. (2017). An Enolate Ion as a Synthon in Biocatalytic Synthesis of 3,4-Dihydro-2(1: H)-Quinoxalinones and 3,4-Dihydro-1,4-Benzoxazin-2-Ones: Lemon Juice as an Alternative to Hazardous Solvents and Catalysts. Green Chem..

[29] Joksimović N., Janković N., Petronijević J., Baskić D., Popovic S., Todorović D., Zarić M., Klisurić O., Vraneš M., Tot A. (2019). Synthesis, Anticancer Evaluation and Synergistic Effects with Cis Platin of Novel Palladium Complexes: DNA, BSA Interactions and Molecular Docking Study. Med. Chem..

[30] Nakamoto K. (2008). Infrared and Raman Spectra of Inorganic and Coordination Compounds.

[31] Zabicky J., Rappoport Z. (2009). The Chemistry of Metal Enolates, 2 Volume Set.

[32] Pellei M., Del Gobbo J., Caviglia M., Gandin V., Marzano C., Karade D.V., Noonikara Poyil A., Dias H.V.R., Santini C. (2024). Synthesis and Investigations of the Antitumor Effects of First-Row Transition Metal(II) Complexes Supported by Two Fluorinated and Non-Fluorinated β-Diketonates. Int. J. Mol. Sci..

[33] Lemire J.A., Harrison J.J., Turner R.J. (2013). Antimicrobial Activity of Metals: Mechanisms, Molecular Targets and Applications. Nat. Rev. Microbiol..

[34] Sirajuddin M., Ali S., Badshah A. (2013). Drug—DNA Interactions and Their Study by UV—Visible, Fluorescence Spectroscopies and Cyclic Voltametry. J. Photochem. Photobiol. B.

[35] Singh K., Barwa M.S., Tyagi P. (2006). Synthesis, Characterization and Biological Studies of Co(II), Ni(II), Cu(II) and Zn(II) Complexes with Bidentate Schiff Bases Derived by Heterocyclic Ketone. Eur. J. Med. Chem..

[36] Abdel Aziz A.A., Salem A.N.M., Sayed M.A., Aboaly M.M. (2012). Synthesis, Structural Characterization, Thermal Studies, Catalytic Efficiency and Antimicrobial Activity of Some M(II) Complexes with ONO Tridentate Schiff Base N-Salicylidene-o-Aminophenol (SaphH 2). J. Mol. Struct..

[37] Garoufis A., Hadjikakou S.K., Hadjiliadis N. (2009). Palladium Coordination Compounds as Anti-Viral, Anti-Fungal, Anti-Microbial and Anti-Tumor Agents. Coord. Chem. Rev..

[38] Lippert B., Sanz Miguel P.J. (2018). Comparing PtII- and PdII-Nucleobase Coordination Chemistry: Why PdII Not Always Is a Good Substitute for PtII. Inorganica Chim. Acta.

[39] Silhavy T.J., Kahne D., Walker S. (2010). The Bacterial Cell Envelope. Cold Spring Harb. Perspect. Biol..

[40] Nikaido H. (2003). Molecular Basis of Bacterial Outer Membrane Permeability Revisited. Microbiol. Mol. Biol. Rev..

[41] Li X.Z., Plésiat P., Nikaido H. (2015). The Challenge of Efflux-Mediated Antibiotic Resistance in Gram-Negative Bacteria. Clin. Microbiol. Rev..

[42] Gow N.A.R., Latge J.-P., Munro C.A. (2017). The Fungal Cell Wall: Structure, Biosynthesis, and Function. Microbiol. Spectr..

[43] Ćwiklińska-Jurkowska M., Wiese-Szadkowska M., Janciauskiene S., Paprocka R. (2023). Disparities in Cisplatin-Induced Cytotoxicity—A Meta-Analysis of Selected Cancer Cell Lines. Molecules.

[44] Benyettou F., Fahs H., Elkharrag R., Bilbeisi R.A., Asma B., Rezgui R., Motte L., Magzoub M., Brandel J., Olsen J.-C. (2017). Selective Growth Inhibition of Cancer Cells with Doxorubicin-Loaded CB[7]-Modified Iron-Oxide Nanoparticles. RSC Adv..

[45] Janković N., Popović S., Baskić D., Matić S., Markovic T., Petković M., Milenković D., Vraneš M. (2026). Salicylate-Based Biocompatible Ionic Liquids as Selective Modulators of Antineoplastic Agents Cisplatin and Doxorubicin. Sci. Rep..

[46] Arokia Femina T., Barghavi V., Archana K., Swethaa N.G., Maddaly R. (2023). Non-Uniformity in in Vitro Drug-Induced Cytotoxicity as Evidenced by Differences in IC50 Values—Implications and Way Forward. J. Pharmacol. Toxicol. Methods.

[47] González-Larraza P.G., López-Goerne T.M., Padilla-Godínez F.J., González-López M.A., Hamdan-Partida A., Gómez E. (2020). IC50 Evaluation of Platinum Nanocatalysts for Cancer Treatment in Fibroblast, HeLa, and DU-145 Cell Lines. ACS Omega.

[48] Bartmańska A., Tronina T., Popłoński J., Milczarek M., Filip-Psurska B., Wietrzyk J. (2018). Highly Cancer Selective Antiproliferative Activity of Natural Prenylated Flavonoids. Molecules.

[49] Tronina T., Bartmańska A., Popłoński J., Rychlicka M., Sordon S., Filip-Psurska B., Milczarek M., Wietrzyk J., Huszcza E. (2023). Prenylated Flavonoids with Selective Toxicity against Human Cancers. Int. J. Mol. Sci..

[50] Kostova I. (2006). Platinum Complexes as Anticancer Agents. Recent Pat. Anti-Cancer Drug Discov..

[51] Marques M.P.M. (2013). Platinum and Palladium Polyamine Complexes as Anticancer Agents: The Structural Factor. ISRN Spectrosc..

[52] Li Y., Gu Z., Zhang C., Li S., Zhang L., Zhou G., Wang S., Zhang J. (2018). Synthesis, Characterization and ROS-Mediated Antitumor Effects of Palladium(II) Complexes of Curcuminoids. Eur. J. Med. Chem..

[53] Haribabu J., Srividya S., Mahendiran D., Gayathri D., Venkatramu V., Bhuvanesh N., Karvembu R. (2020). Synthesis of Palladium(II) Complexes via Michael Addition: Antiproliferative Effects through ROS-Mediated Mitochondrial Apoptosis and Docking with SARS-CoV-2. Inorg. Chem..

[54] Franich A.A., Živković M.D., Milovanović J., Arsenijević D., Arsenijević A., Milovanović M., Djuran M.I., Rajković S. (2020). In Vitro Cytotoxic Activities, DNA- and BSA-Binding Studies of Dinuclear Palladium(II) Complexes with Different Pyridine-Based Bridging Ligands. J. Inorg. Biochem..

[55] Galluzzi L., Senovilla L., Vitale I., Michels J., Martins I., Kepp O., Castedo M., Kroemer G. (2012). Molecular Mechanisms of Cisplatin Resistance. Oncogene.

[56] Hanahan D., Weinberg R.A. (2011). Hallmarks of Cancer: The next Generation. Cell.

[57] Feldman B.J., Feldman D. (2001). The development of androgen-independent prostate cancer. Nat. Rev. Cancer.

[58] Kelland L. (2007). The Resurgence of Platinum-Based Cancer Chemotherapy. Nat. Rev. Cancer.

[59] Gottesman M.M. (2002). Mechanisms of Cancer Drug Resistance. Annu. Rev. Med..

[60] Gligorijević N., Todorović T., Radulović S., Sladić D., Filipović N., Godevac D., Jeremić D., Andelković K. (2009). Synthesis and Characterization of New Pt(II) and Pd(II) Complexes with 2-Quinolinecarboxaldehyde Selenosemicarbazone: Cytotoxic Activity Evaluation of Cd(II), Zn(II), Ni(II), Pt(II) and Pd(II) Complexes with Heteroaromatic Selenosemicarbazones. Eur. J. Med. Chem..

[61] Hassona S.M., Saad E.A., Kiwan H.A., Hassanien M.M. (2022). Palladium(II) Schiff Base Complex Arrests Cell Cycle at Early Stages, Induces Apoptosis, and Reduces Ehrlich Solid Tumor Burden: A New Candidate for Tumor Therapy. Investig. New Drugs.

[62] Darzynkiewicz Z., Halicka H.D., Zhao H. (2010). Analysis of cellular DNA content by flow and laser scanning cytometry. Adv. Exp. Med. Biol..

[63] Kacar O., Cevatemre B., Hatipoglu I., Arda N., Ulukaya E., Yilmaz V.T., Acilan C. (2017). The Role of Cell Cycle Progression for the Apoptosis of Cancer Cells Induced by Palladium(II)-Saccharinate Complexes of Terpyridine. Bioorg. Med. Chem..

[64] Szewczyk O.K., Roszczenko P., Gulia N., Fornalski J., Gumienna A., Bielawski K., Szafert S., Czarnomysy R. (2025). Apoptosis, Oxidative Stress, and Cell Cycle Disruption: Antitumor Effects of a Novel Palladium(II) Complexes. J. Med. Chem..

[65] Gao E., Lin L., Liu L., Zhu M., Wang B., Gao X. (2012). Two New Palladium(Ii) Complexes: Synthesis, Characterization and Their Interaction with HeLa Cells. Dalton Trans..

[66] Mansour A.M., Radacki K., Shehab O.R., Mostafa G.A.E., Ali E.A., Abo-Elfadl M.T. (2025). Cytotoxicity of Pd(Ii) and Pt(Ii) Complexes of 2′,6′-Di(Thiazol-2-Yl)-2,4′-Bipyridine: Insights into the Mode of Cell Death and Cell Cycle Arrest. RSC Adv..

[67] Kacar O., Adiguzel Z., Yilmaz V.T., Cetin Y., Cevatemre B., Arda N., Baykal A.T., Ulukaya E., Acilan C. (2014). Evaluation of the Molecular Mechanisms of a Palladium(II) Saccharinate Complex with Terpyridine as an Anticancer Agent. Anticancer Drugs.

[68] Čanović P., Bogojeski J., Košarić J.V., Marković S.D., Živanović M.N. (2017). Pt(IV), Pd(II), and Rh(III) Complexes Induced Oxidative Stress and Cytotoxicity in the HCT-116 Colon Cancer Cell Line. Turk. J. Biol..

[69] Valentini A., Conforti F., Crispini A., De Martino A., Condello R., Stellitano C., Rotilio G., Ghedini M., Federici G., Bernardini S. (2009). Synthesis, Oxidant Properties, and Antitumoral Effects of a Heteroleptic Palladium(II) Complex of Curcumin on Human Prostate Cancer Cells. J. Med. Chem..

[70] Wei X., Yang Y., Ge J., Lin X., Liu D., Wang S., Zhang J., Zhou G., Li S. (2020). Synthesis, Characterization, DNA/BSA Interactions and in Vitro Cytotoxicity Study of Palladium(II) Complexes of Hispolon Derivatives. J. Inorg. Biochem..

[71] Loftus L.V., Amend S.R., Pienta K.J. (2022). Interplay between Cell Death and Cell Proliferation Reveals New Strategies for Cancer Therapy. Int. J. Mol. Sci..

[72] Fetzer L., Boff B., Ali M., Xiangjun M., Collin J.P., Sirlin C., Gaiddon C., Pfeffer M. (2011). Library of Second-Generation Cycloruthenated Compounds and Evaluation of Their Biological Properties as Potential Anticancer Drugs: Passing the Nanomolar Barrier. Dalton Trans..

[73] Li J., Tian M., Tian Z., Zhang S., Yan C., Shao C., Liu Z. (2018). Half-Sandwich Iridium(III) and Ruthenium(II) Complexes Containing P^P-Chelating Ligands: A New Class of Potent Anticancer Agents with Unusual Redox Features. Inorg. Chem..

[74] Puckett C.A., Barton J.K. (2007). Methods to Explore Cellular Uptake of Ruthenium Complexes. J. Am. Chem. Soc..

[75] Ghose A.K., Viswanadhan V.N., Wendoloski J.J. (1999). A Knowledge-Based Approach in Designing Combinatorial or Medicinal Chemistry Libraries for Drug Discovery. 1. A Qualitative and Quantitative Characterization of Known Drug Databases. J. Comb. Chem..

[76] Ašanin D.P., Bogojevic S.S., Perdih F., Andrejević T.P., Milivojevic D., Aleksic I., Nikodinovic-Runic J., Glišić B., Turel I., Djuran M.I. (2021). Structural Characterization, Antimicrobial Activity and BSA/DNA Binding Affinity of New Silver(I) Complexes with Thianthrene and 1,8-Naphthyridine. Molecules.

[77] Kumar N., Kaushal R., Awasthi P. (2023). Non-Covalent Binding Studies of Transition Metal Complexes with DNA: A Review. J. Mol. Struct..

[78] Drew H.R., Wing R.M., Takano T., Broka C., Tanaka S., Itakura K., Dickerson R.E. (1981). Structure of a B-DNA Dodecamer: Conformation and Dynamics. Proc. Natl. Acad. Sci. USA.

[79] Gao E.J., Wang L., Zhu M.C., Liu L., Zhang W.Z. (2010). Synthesis, Characterization, Interaction with DNA and Cytotoxicity in Vitro of the Complexes [M(Dmphen)(CO3)]·H2O [M = Pt(II), Pd(II)]. Eur. J. Med. Chem..

[80] Chen L.M., Liu J., Chen J.C., Tan C.P., Shi S., Zheng K.C., Ji L.N. (2008). Synthesis, Characterization, DNA-Binding and Spectral Properties of Complexes [Ru(L)4(Dppz)]2+ (L = Im and MeIm). J. Inorg. Biochem..

[81] Kemp S., Wheate N.J., Buck D.P., Nikac M., Collins J.G., Aldrich-Wright J.R. (2007). The Effect of Ancillary Ligand Chirality and Phenanthroline Functional Group Substitution on the Cytotoxicity of Platinum(II)-Based Metallointercalators. J. Inorg. Biochem..

[82] Zhou C.Y., Zhao J., Wu Y.B., Yin C.X., Pin Y. (2007). Synthesis, Characterization and Studies on DNA-Binding of a New Cu(II) Complex with N1,N8-Bis(l-Methyl-4-Nitropyrrole-2-Carbonyl)Triethylenetetramine. J. Inorg. Biochem..

[83] Wang Y.Q., Zhang H.M., Zhang G.C., Tao W.H., Tang S.H. (2007). Binding of Brucine to Human Serum Albumin. J. Mol. Struct..

[84] Meloun B., Moravek L., Kostka V. (1975). Complete amino acid sequence of human serum albumin. FEBS Lett..

[85] Zunszain P.A., Ghuman J., Komatsu T., Tsuchida E., Curry S. (2003). Crystal Structural Analysis of Human Serum Albumin Complexed with Hemin and Fatty Acid. BMC Struct. Biol..

[86] Yamasaki K., Maruyama T., Kragh-Hansen U., Otagiri M. (1996). Characterization of Site I on Human Serum Albumin: Concept about the Structure of a Drug Binding Site. Biochim. Biophys. Acta..

[87] Sułkowska A. (2002). Interaction of Drugs with Bovine and Human Serum Albumin. J. Mol. Struct..

[88] Trynda-Lemiesz L. (2004). Paclitaxel-HSA Interaction. Binding Sites on HSA Molecule. Bioorg. Med. Chem..

[89] Divsalar A., Saboury A.A., Ahadi L., Zemanatiyar E., Mansouri-Torshizi H., Ajloo D., Sarma R.H. (2011). Biological Evaluation and Interaction of a Newly Designed Anti-Cancer Pd(II) Complex and Human Serum Albumin. J. Biomol. Struct. Dyn..

[90] The European Committee on Antimicrobial Susceptibility Testing. Breakpoint Tables for Interpretation of MICs and Zone Diameters. Version V.16.1 2026. http://www.eucast.org.

[91] The European Committee on Antimicrobial Susceptibility Testing. Media Preparation for EUCAST Disk Diffusion Testing and for Determination of MIC Values by the Broth Microdilution Method. V.6.0; January 2020. http://www.eucast.org.

[92] Mosmann T. (1983). Rapid Colorimetric Assay for Cellular Growth and Survival: Application to Proliferation and Cytotoxicity Assays. J. Immunol. Methods.

[93] Badisa R., Darling-Reed S., Joseph P., Cooperwood J., Latinwo L., Goodman C. (2009). Selective Sytotoxic Activities of Two Novel Synthetic Drugs on Human Breast Carcinoma MCF-7 Cells. Anticancer Res..

[94] Sova M., Žižak Ž., Stanković J.A., Prijatelj M., Turk S., Juranić Z.D., Mlinarič-Raščan I., Gobec S. (2013). Cinnamic acid derivatives induce cell cycle arrest in carcinoma cell lines. Med. Chem..

[95] Weigend F., Ahlrichs R. (2005). Balanced Basis Sets of Split Valence, Triple Zeta Valence and Quadruple Zeta Valence Quality for H to Rn: Design and Assessment of Accuracy. Phys. Chem. Chem. Phys..

[96] Becke A.D. (1988). Density-Fnnctional Exchange-Energy Approximation with Correct Asymptotic Behavior. Phys. Rev. A.

[97] Becke A.D. (1993). Density-Functional Thermochemistry. III. The Role of Exact Exchange. J. Chem. Phys..

[98] Lee C., Yang W., Parr R.G. (1988). Development of the Colle–Salvetti correlation-energy formula into a functional of the electron density. Phys. Rev. B.

[99] Frisch M.J., Trucks G.W., Schlegel H.B., Scuseria G.E., Robb M.A., Cheeseman J.R., Scalmani G., Barone V., Mennucci B., Petersson G.A. (2009). Gaussian 09, Revision B.01.

[100] Lu T. (2021). Simple, Reliable, and Universal Metrics of Molecular Planarity. J. Mol. Model..

[101] Trott O., Olson A.J. (2010). AutoDock Vina: Improving the Speed and Accuracy of Docking with a New Scoring Function, Efficient Optimization, and Multithreading. J. Comput. Chem..

[102] BIOVIA (2025). Discovery Studio 2025.

