# Supplementary material for: Novel Palladium(II) β-Diketonate Complexes: Synthesis, Dual Antimicrobial/Anticancer Activity, and Molecular Insights into DNA/Protein Interactions
Source: Int J Mol Sci. 2026 Oct 23;27(21):9437. doi: 10.3390/ijms27219437 (PMC13763340; doi:10.3390/ijms27219437)
Supplement: Supplementary file 1 [file ijms-27-09437-s001.zip › ijms-4571021-supplementary.pdf]

International Journal of
Molecular Sciences

MDPI

Supplementary Material associated with the paper

Novel Palladium(II) β-Diketonate Complexes: Synthesis, Dual Antimicrobial/Anticancer Activity, and Molecular Insights into DNA/Protein Interactions

Marija Trifunović Stevanović [1], Nenad Joksimović [1,*], Jelena Petronijević [1], Snežana Stojanović [1,*], Slađana Đorđević [1], Jelena Antić Stanković [2], Biljana Bufan [2,¶] and Dragana D. Božić [2,¶]

[1] University of Kragujevac, Faculty of Science, Department of Chemistry, Radoja Domanovića 12, 34000 Kragujevac, Serbia
[2] University of Belgrade, Faculty of Pharmacy, Department of Microbiology and Immunology, Vojvode Stepe 450, 11221 Belgrade, Serbia
¶ both authors contributed equally
* Correspondence: nenad.joksimovic@pmf.kg.ac.rs; snezana.stojanovic@pmf.kg.ac.rs

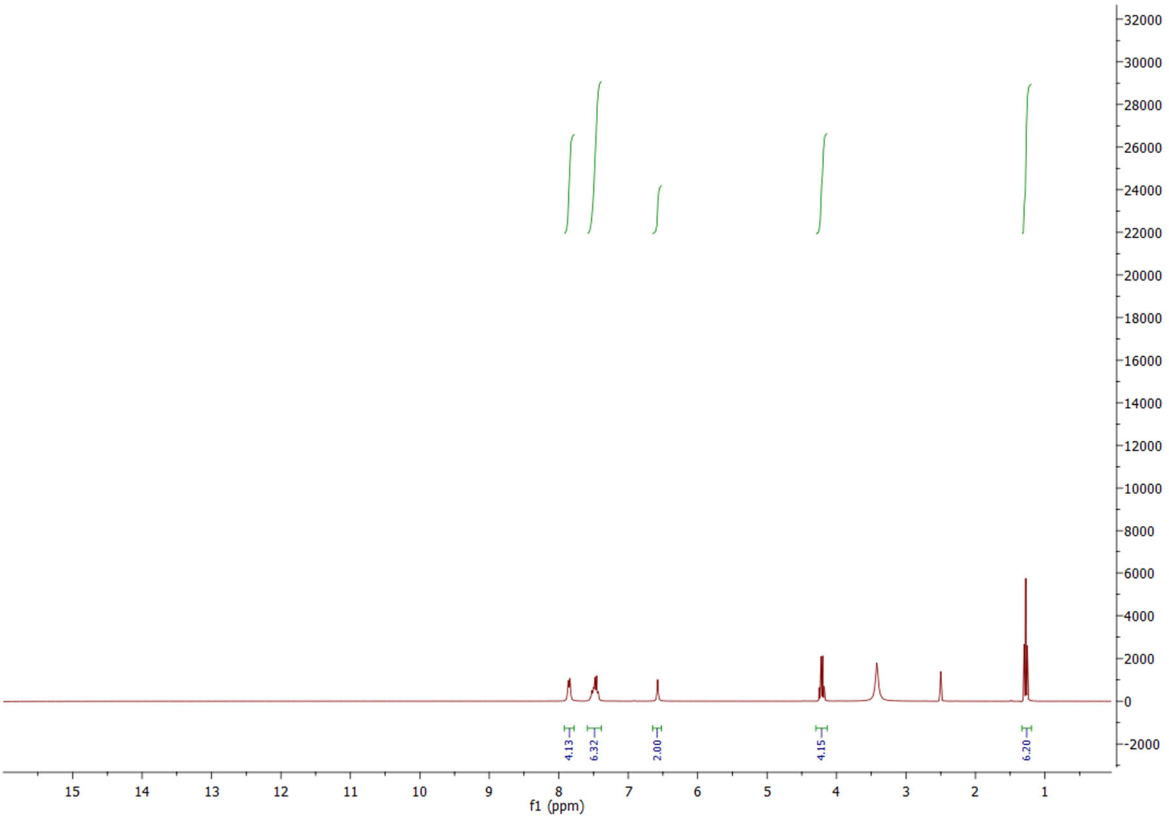

Fig S1.^{1}H NMR spectrum of **B1**

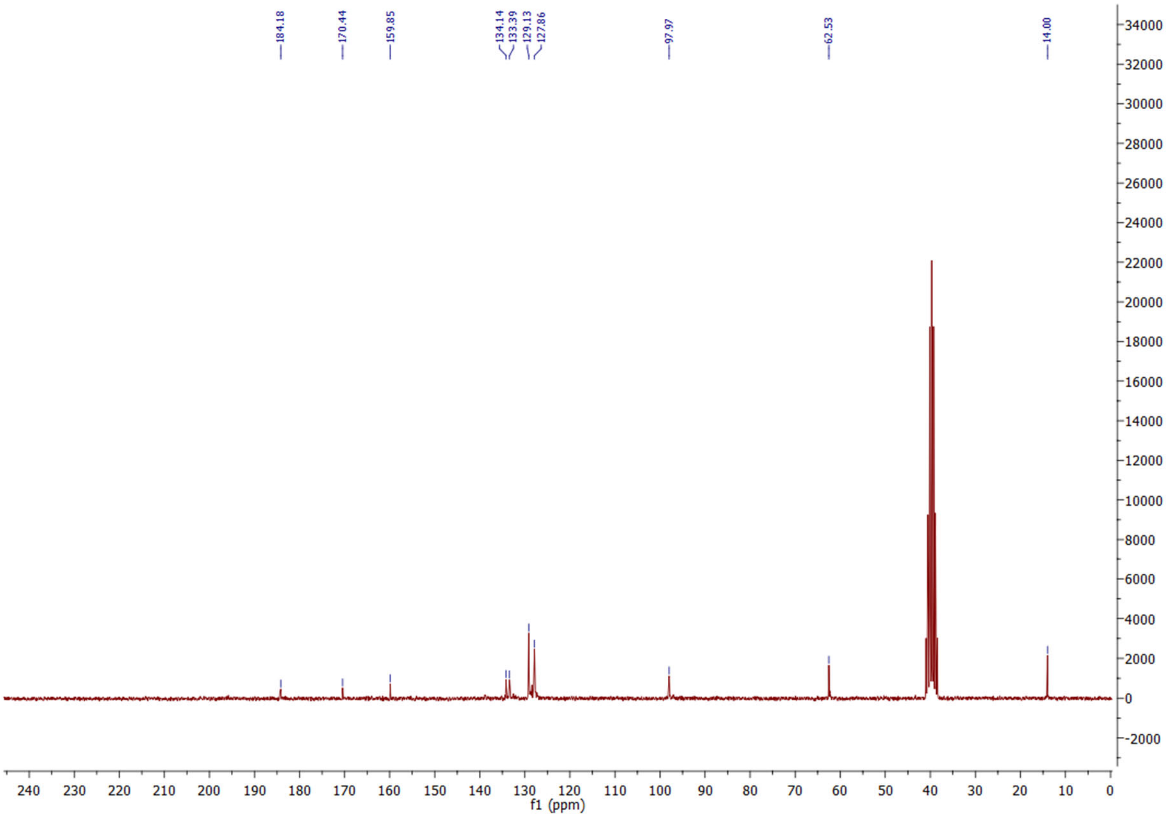

Fig S2.^{13}C NMR spectrum of **B1**

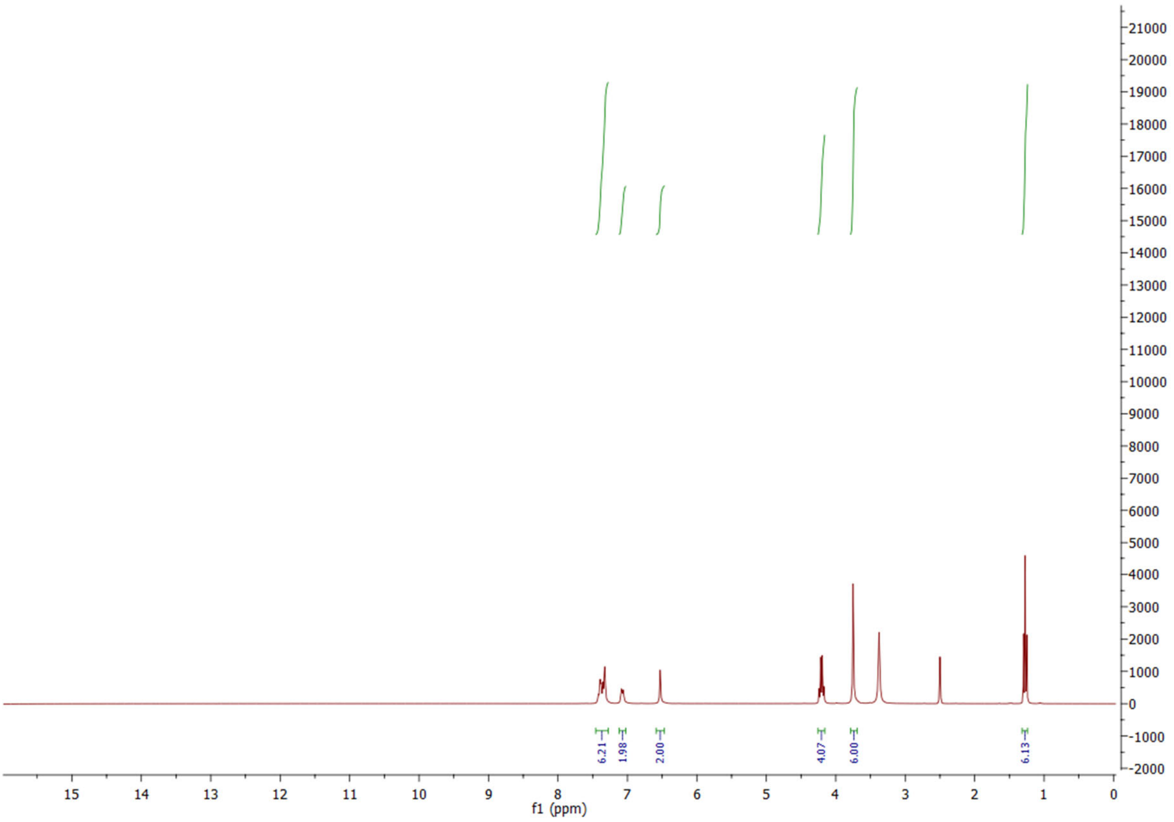

Fig S3.^{1}H NMR spectrum of **B2**

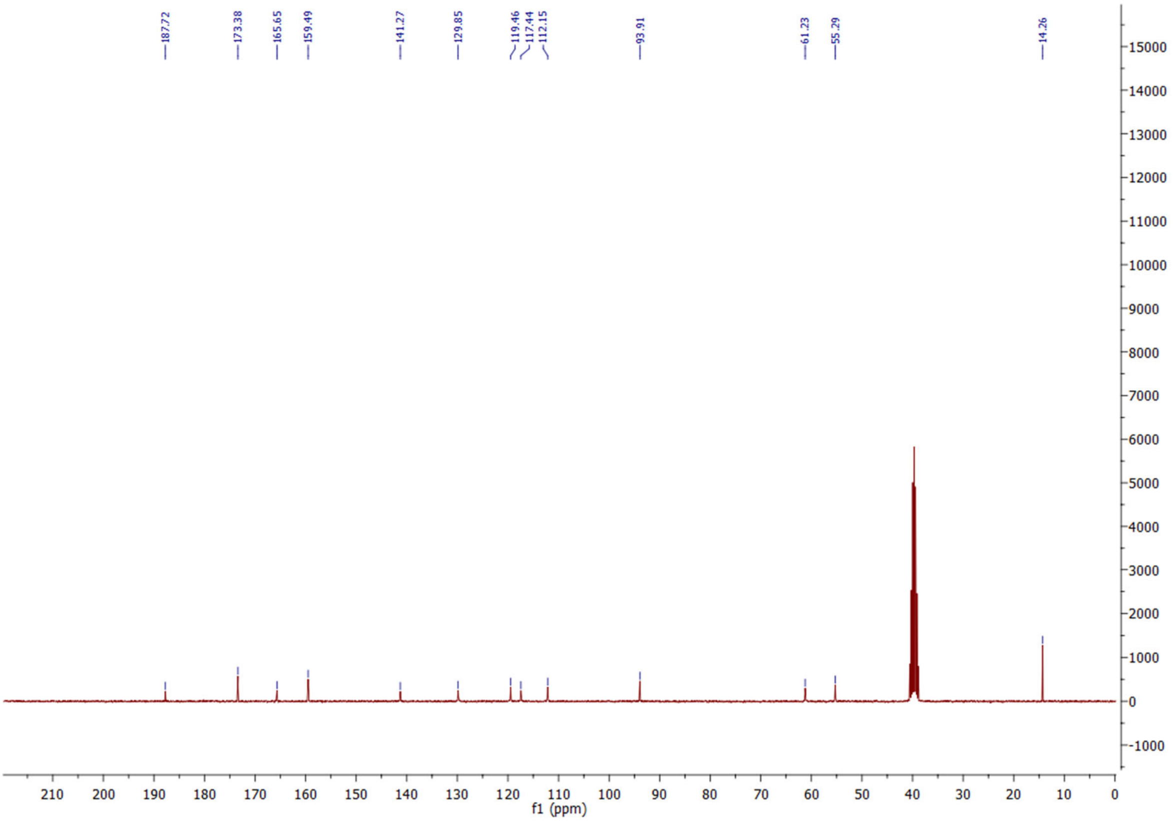

Fig S4.^{13}C NMR spectrum of **B2**

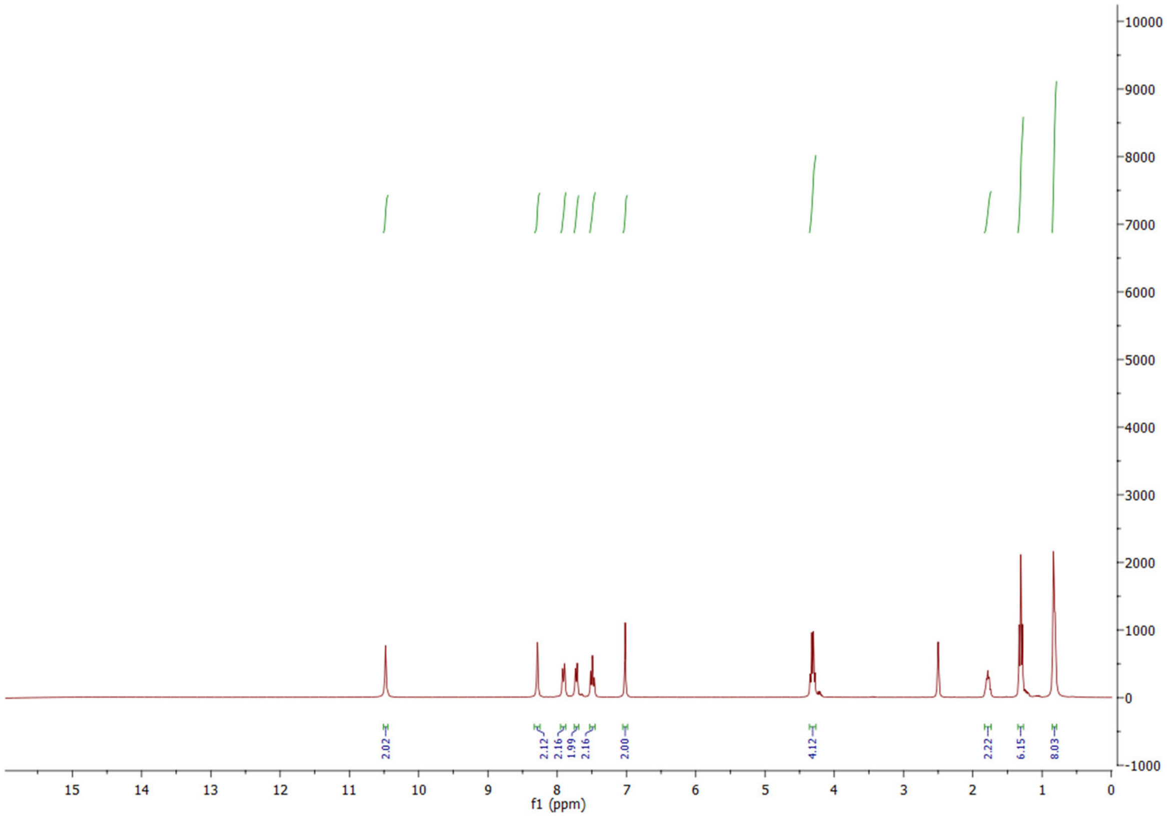

Fig S5.^{1}H NMR spectrum of **B3**

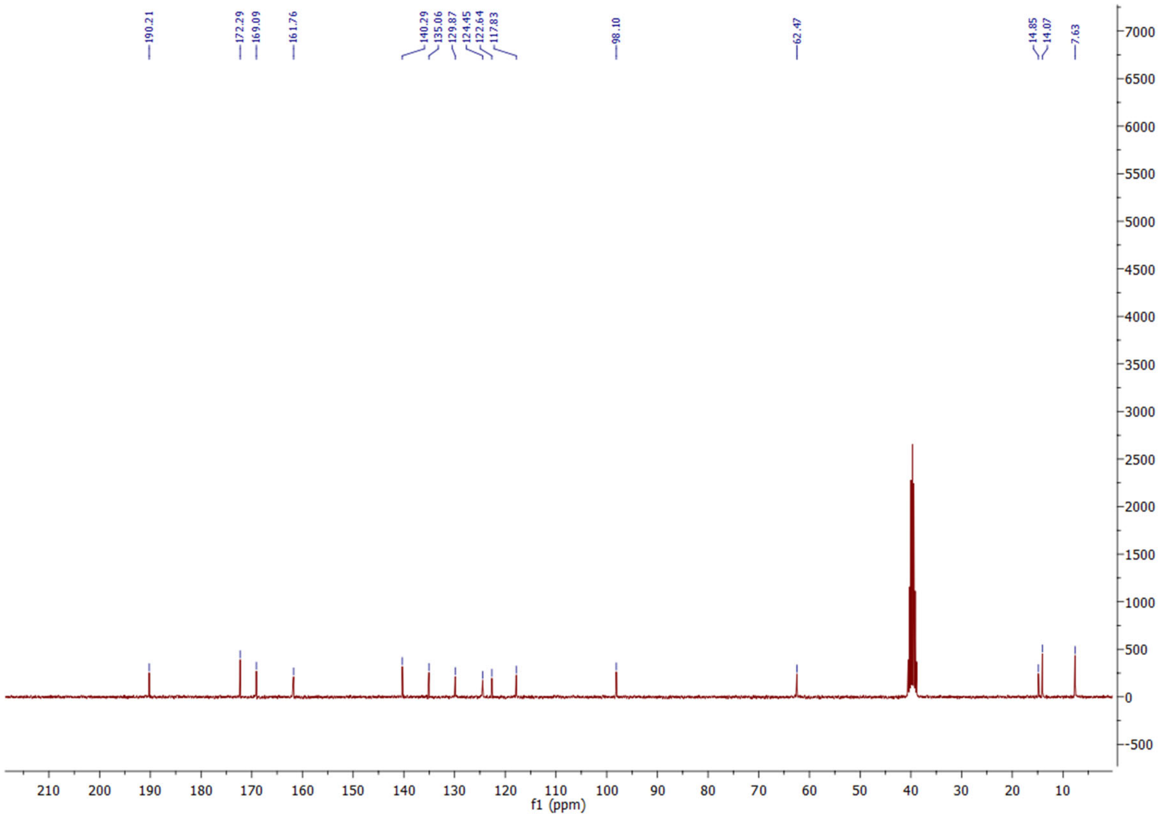

Fig S6.^{13}C NMR spectrum of **B3**

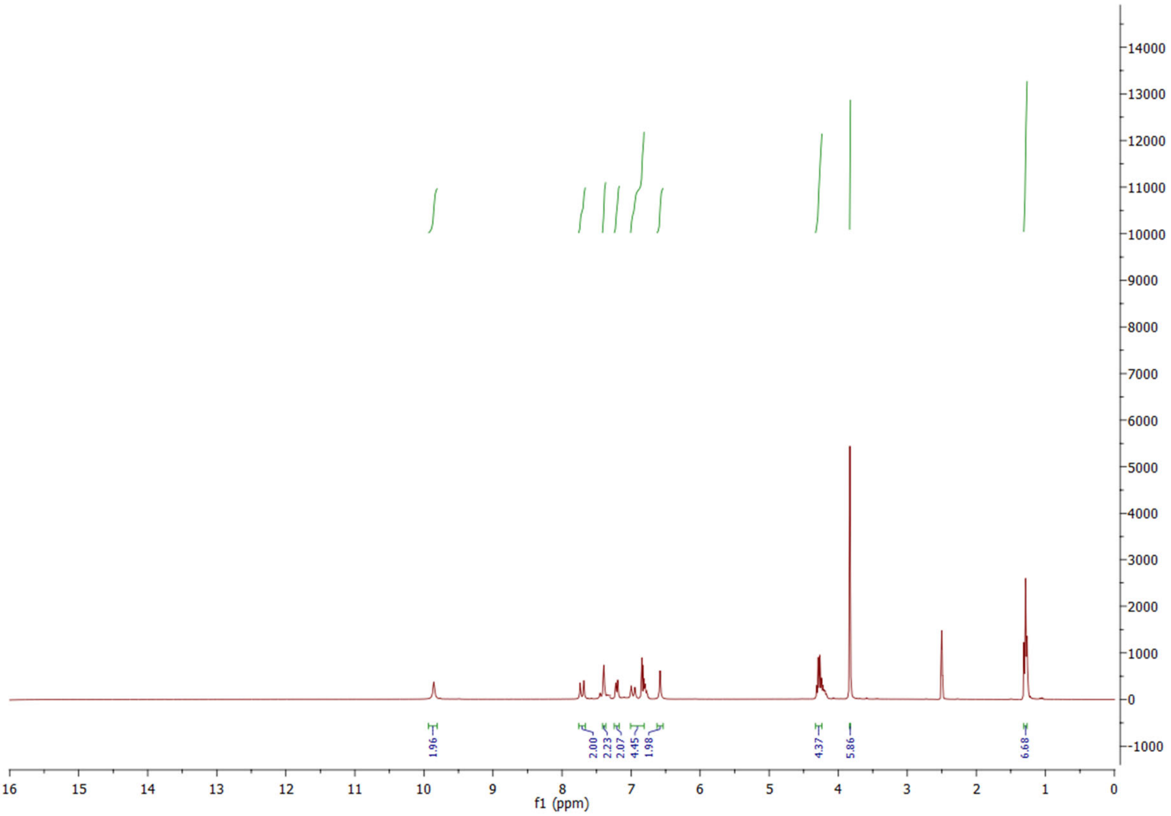

Fig S7.¹H NMR spectrum of **B4**

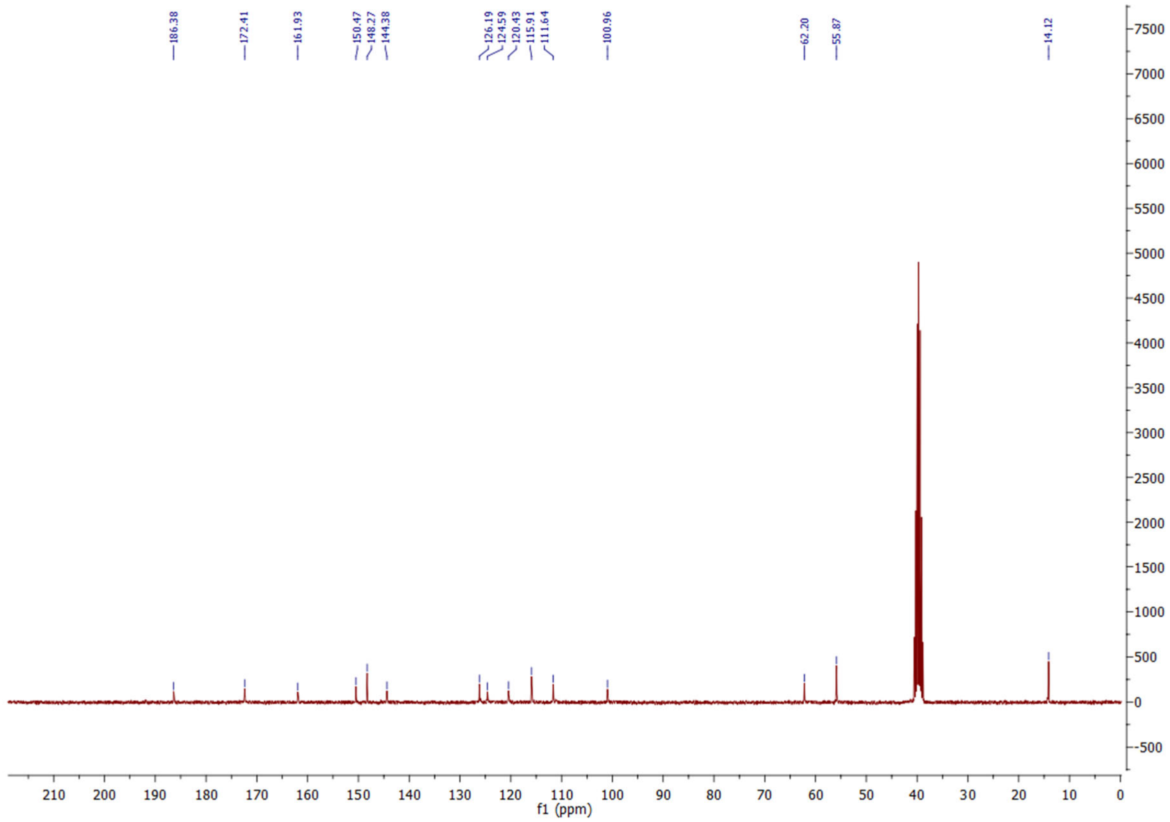

Fig S8.¹³C NMR spectrum of **B4**

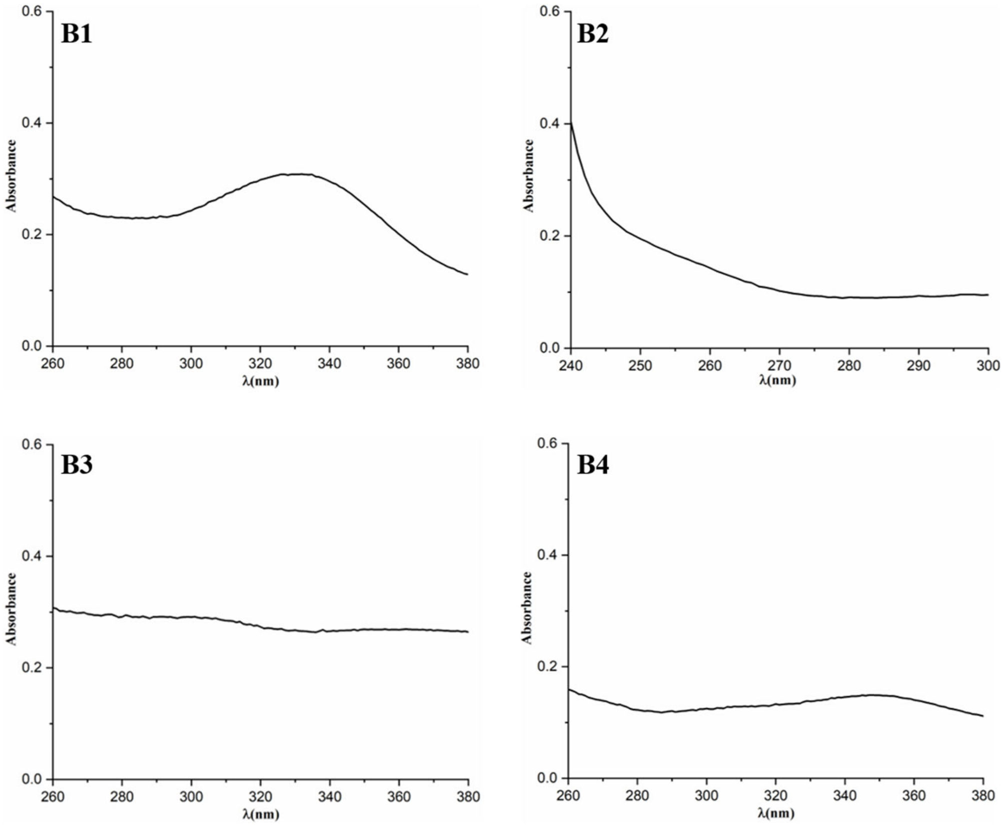

Fig S9. Time-dependent UV-Vis stability study of complexes **B1–4** in PBS buffer at room temperature (the complexes were initially dissolved in DMSO at a concentration of 1×10^{-3} M and subsequently diluted with PBS buffer to a final concentration of 1.35×10^{-5} M).

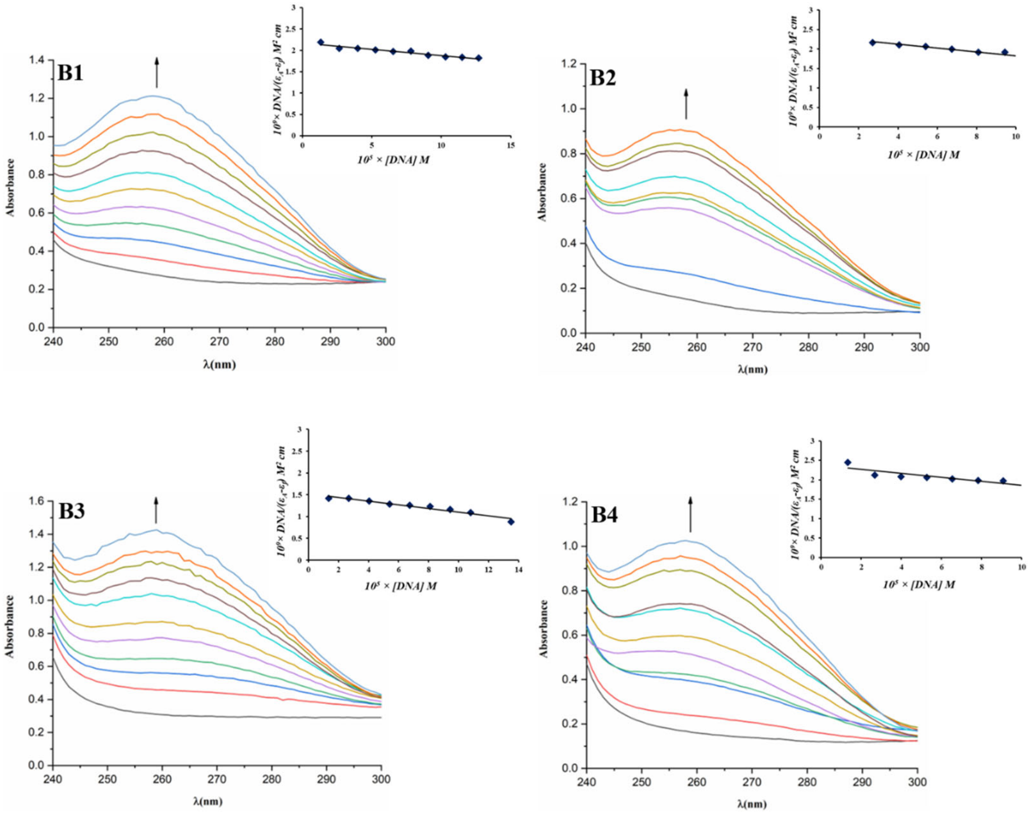

Fig S10. Absorption spectra of the complexes **B1–4** after adding DNA, measured in PBS buffer. [**B1–4**] = 1.35 × 10^{-5} M [DNA] = 1.35 – 13.5 × 10^{-5} M;. The arrows show the change in absorbance after addition of DNA. Insert graph: plot [DNA]/(εa-εf) *vs.* [DNA].

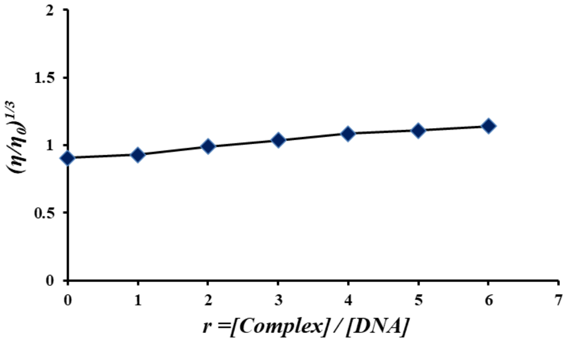

Fig S11. Relative viscosity $(\eta/\eta_0)^{1/3}$ of CT-DNA (1.35 × 10^{-5} M) in PBS solution in the presence of increasing amounts of **B2** (1.35 – 8.10 × 10^{-5} M).

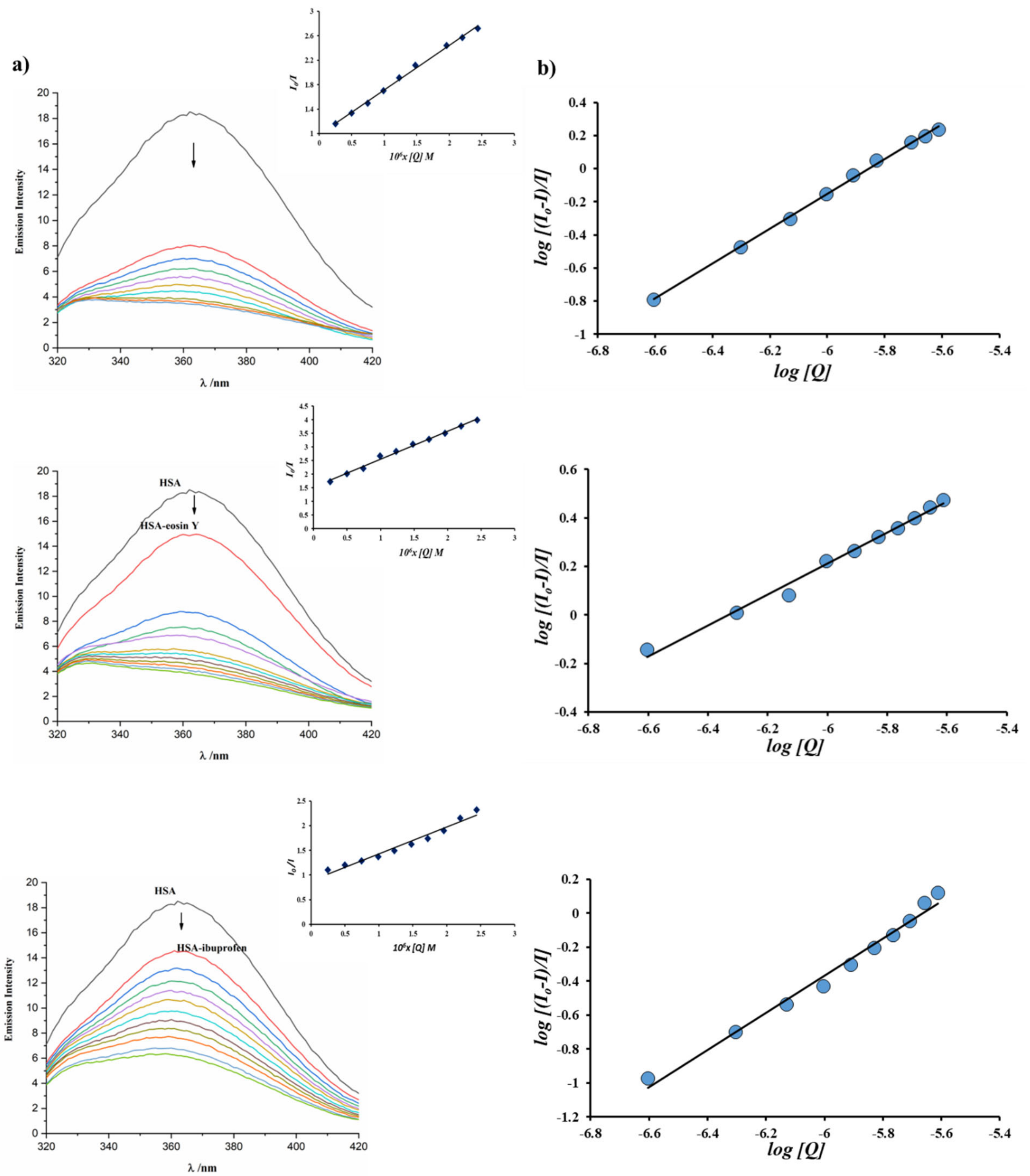

Fig S12. **a)** Emission spectra of HSA/HSA – eosin Y/HSA - ibuprofen in the presence of complex **B1**. [**B1**] = 2.49 – 24.4 × 10^{-7} M, [HSA] = [eosin Y] = [ibuprofen] = 2.00 × 10^{-6} M. The arrows show the change in emission intensity after the addition of complex. Insert graph: plots of I_0/I *vs.* [Q] **b)** Plot of log[(I_0-I)/I] *vs.* log[Q]

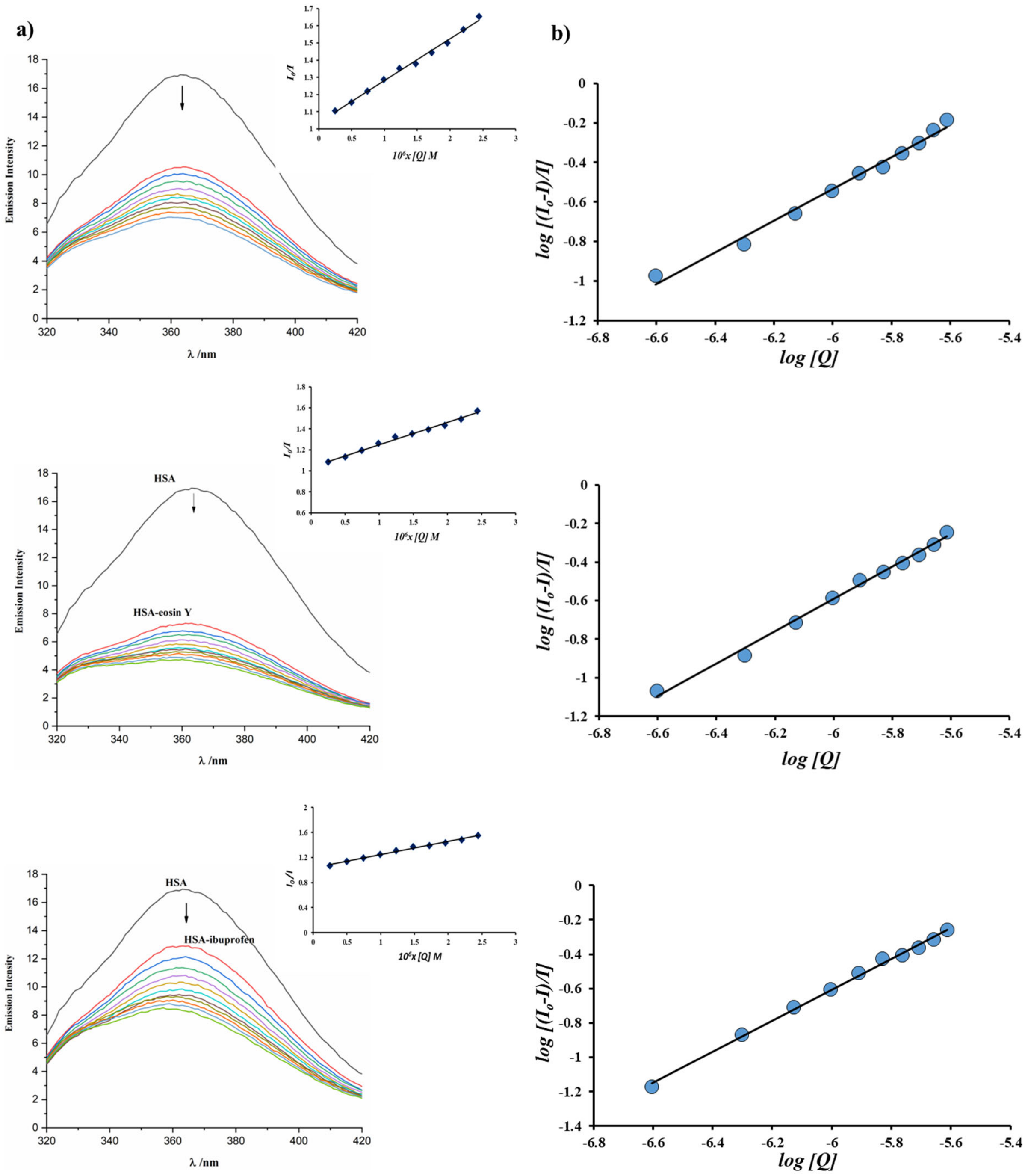

Fig S13. a) Emission spectra of HSA/HSA – eosin Y/HSA - ibuprofen in the presence of complex **B3**. [**B3**] = 2.49 – 24.4 × 10^{-7} M, [HSA] = [eosin Y] = [ibuprofen] = 2.00 × 10^{-6} M. The arrows show the change in emission intensity after the addition of complex. Insert graph: plots of I_0/I *vs.* [Q] **b)** Plot of log[(I_0-I)/I] *vs.* log[Q]

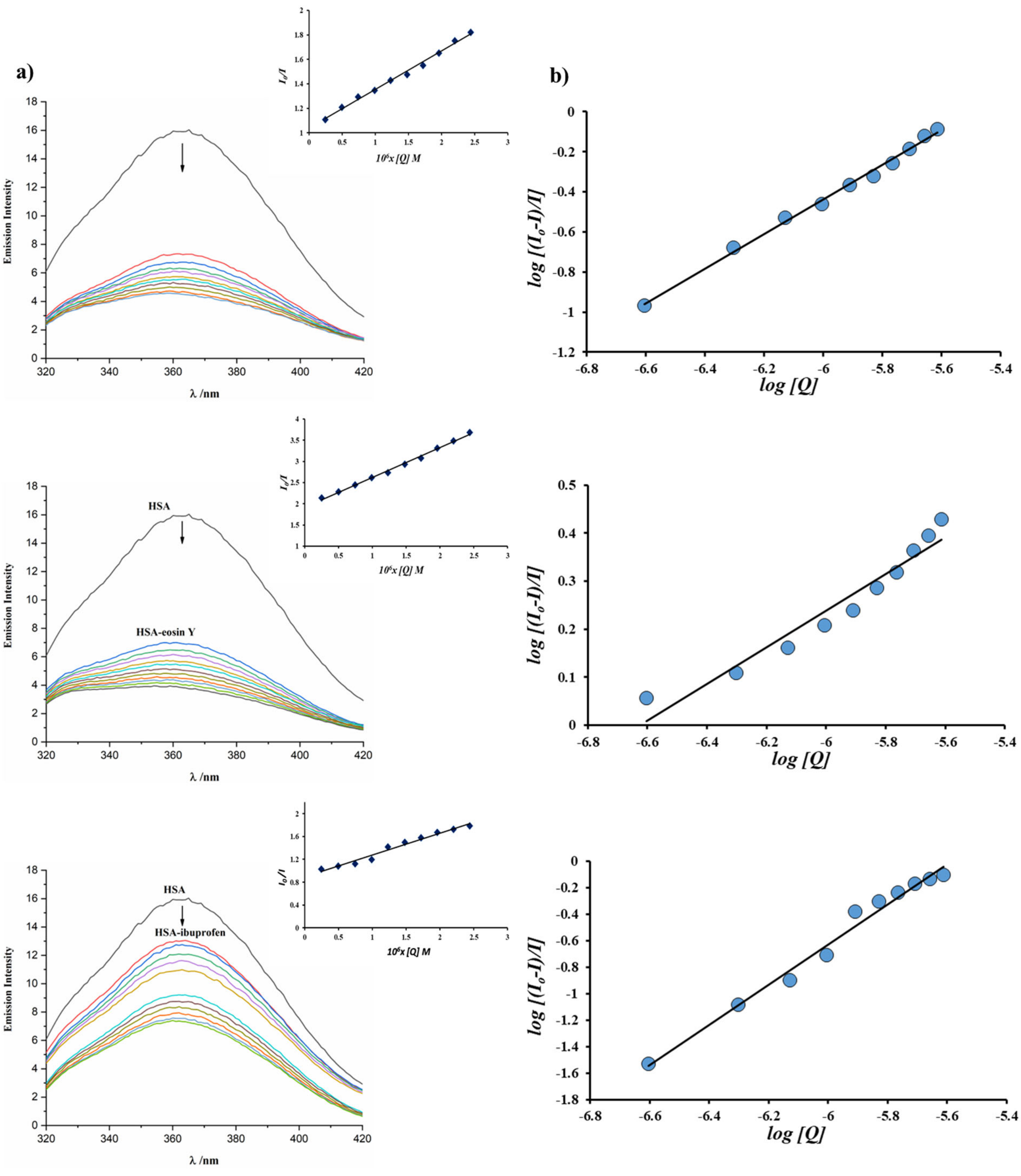

Fig S14. a) Emission spectra of HSA/HSA – eosin Y/HSA - ibuprofen in the presence of complex **B4**. [**B4**] = 2.49 – 24.4 × 10^{-7} M, [HSA] = [eosin Y] = [ibuprofen] = 2.00 × 10^{-6} M. The arrows show the change in emission intensity after the addition of complex. Insert graph: plots of I_0/I *vs.* [Q] **b)** Plot of log[(I_0-I)/I] *vs.* log[Q]

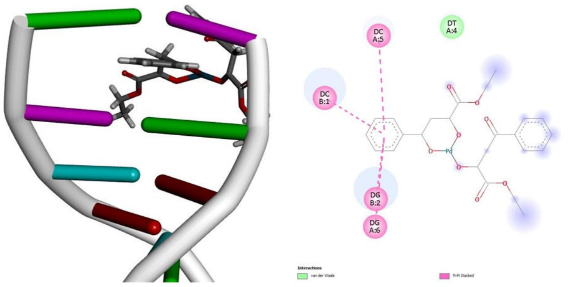

B1

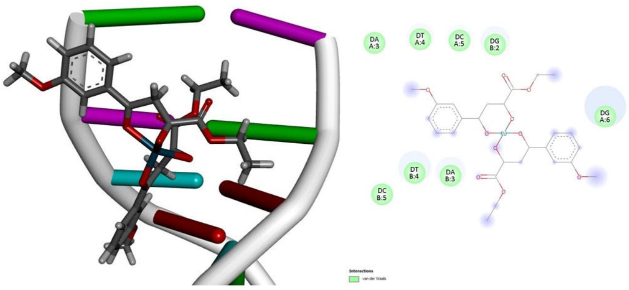

B2

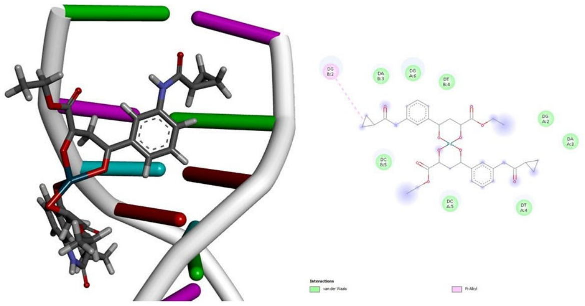

B3

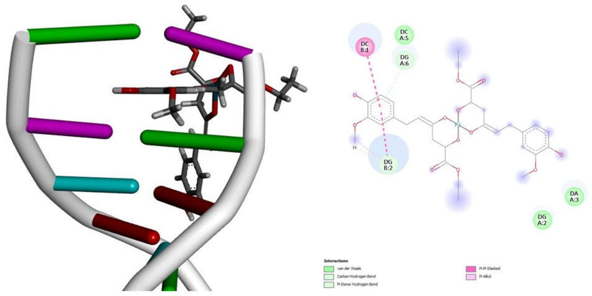

B4

Fig S15. Best docking scenarios and 2D interaction diagram for complexes **B1–4** with DNA with an intercalation gap (PDB ID: 1Z3F).

Table S1. Comparison of minimum inhibitory concentrations (MICs) of complexes **B1–4** expressed in µM and µg/mL.

	**MIC µM (µg/mL)**			
**Microorganism**	**B1**	**B2**	**B3**	**B4**
Staphylococcus aureus	625 (340)	625 (380)	312 (220)	312 (210)
Staphylococcus epidermidis	312 (170)	312 (190)	312 (220)	312 (210)
Enterococcus faecalis	312 (170)	312 (190)	312 (220)	312 (210)
Escherichia coli	800 (435)	1200 (730)	400 (280)	400 (270)
Klebsiella pneumoniae	800 (435)	1200 (730)	400 (280)	400 (270)
Salmonella abony	800 (435)	1200 (730)	800 (560)	400 (270)
Pseudomonas aeruginosa	800 (435)	1200 (730)	1200 (840)	800 (540)
Candida albicans	625 (340)	312 (190)	312 (220)	312 (210)
Cutibacterium acnes	625 (340)	312 (190)	625 (440)	312 (210)

Table S2. Cartesian coordinates (in Å) of the optimized geometries obtained in implicit solution calculations.

**B1**			
C	2.11637300	-0.60279800	-0.94004600
C	2.04137900	0.91655700	-0.97268100
C	1.90273000	1.48345400	0.41763700
C	-2.60474900	0.08041400	-1.94607700

C	-3.02327200	-1.14939700	-1.15822500
C	-2.43058400	-1.22580900	0.25549900
H	2.87078300	1.38167600	-1.50753200
H	-4.12080500	-1.21444000	-1.07319600
Pd	-0.57963100	-0.37464900	0.51262100
O	-2.57275500	-0.23383500	1.09657000
O	-1.64125400	0.00801500	-2.70529100
O	1.15435400	-1.23753400	-0.48888100
O	0.99734500	1.17947700	1.17371200
C	-2.32519500	-2.63379800	0.79665300
O	-2.22915600	-3.62824000	0.09451100
O	-2.33742200	-2.68039700	2.13457400
C	-2.16922300	-3.97772000	2.75227700
C	-2.21312500	-3.79214400	4.25129000
H	-1.20878800	-4.40198700	2.41953000
H	-2.97243500	-4.64231500	2.39781600
H	-2.08990700	-4.76859500	4.74480200
H	-1.40315200	-3.12917900	4.59371600
H	-3.17661100	-3.36463500	4.57022700
C	-3.36185200	1.36021300	-1.80259600
C	-4.45207200	1.50189000	-0.92413400
C	-2.96141100	2.46684400	-2.57765600
C	-5.12158200	2.72393000	-0.82209100
H	-4.77859000	0.66437100	-0.30763300
C	-3.63260000	3.68336300	-2.47758300
H	-2.11669300	2.35350600	-3.25941400
C	-4.71546000	3.81421600	-1.59772300
H	-5.96493700	2.82396100	-0.13475300
H	-3.31440700	4.53441300	-3.08464500
C	2.98439600	2.47731200	0.88156300
O	3.96752900	2.72378800	0.21607500
O	2.69479500	2.99088900	2.05960500
C	3.63861400	3.94369700	2.62683700
C	3.09413100	4.40333900	3.95690900
H	3.75225500	4.77296300	1.91221400
H	4.61100000	3.43768500	2.72492800
H	3.79472100	5.12602600	4.40263800
H	2.11810800	4.89825400	3.83672600
H	2.98065600	3.55834900	4.65339400
C	3.31003400	-1.31189800	-1.44221700
C	4.53165300	-0.64916500	-1.67777800
C	3.22407900	-2.70272800	-1.66718400
C	5.64141100	-1.36624000	-2.12653600
H	4.63085600	0.41960800	-1.48460100

C	4.33091500	-3.40911600	-2.12855500
H	2.27803900	-3.21507600	-1.48528100
C	5.54194400	-2.74196000	-2.35834000
H	6.58771600	-0.84797100	-2.29654800
H	4.25411000	-4.48336900	-2.31128600
H	6.41092600	-3.29825400	-2.71848500
H	-5.24280400	4.76822000	-1.51785500
H	1.09486300	1.15173600	-1.49365800
H	-2.67857000	-2.02315600	-1.72412100
**B2**			
C	2.79588100	0.22060100	-0.41615900
C	2.38886000	-0.32529900	0.93618500
C	1.16302300	-1.22081700	0.90583000
C	-2.59659900	0.80705500	-0.35382400
C	-3.04192700	-0.63220400	-0.30385700
C	-2.09612300	-1.53459700	-1.09334200
H	3.22098800	-0.90130500	1.37433500
H	-3.06807100	-0.92938800	0.75800300
Pd	-0.11532300	-0.81261100	-0.75545200
O	-1.76315000	-1.22776200	-2.28343600
O	-1.38990300	1.04767200	-0.51191500
O	2.01375000	0.16157700	-1.36659700
O	1.13682900	-2.27404400	0.16942600
C	-2.07407300	-2.99388800	-0.64878300
O	-2.61235700	-3.38549100	0.36985500
O	-1.42028200	-3.77482400	-1.50227700
C	-1.28903500	-5.17703700	-1.15459000
C	-0.54465200	-5.86864300	-2.27176600
H	-2.29715700	-5.59418100	-1.00953900
H	-0.74980000	-5.24193700	-0.19687400
H	-0.43433500	-6.93668800	-2.02859200
H	-1.09210400	-5.78689500	-3.22365500
H	0.46104300	-5.44036700	-2.40446800
C	-3.56005200	1.91680700	-0.16839100
C	-4.93043300	1.68359600	0.06932900
C	-3.07696000	3.23408200	-0.21893400
C	-5.78573600	2.76828300	0.25172500
H	-5.32837600	0.67020300	0.11692500
C	-3.94363800	4.31920300	-0.03877700
H	-2.01906100	3.42670800	-0.40054500
C	-5.30943900	4.08281000	0.19834300
C	0.24805300	-1.11527700	2.11321200
O	0.13252300	-0.09665500	2.77335200
O	-0.38830800	-2.25373000	2.37665000

C	-1.31472800	-2.25907600	3.49253900
C	-1.71951000	-3.68901300	3.76432600
H	-2.17724600	-1.62886000	3.22273500
H	-0.81758300	-1.80130600	4.36050000
H	-2.42729200	-3.71150600	4.60741600
H	-2.21026200	-4.13573600	2.88720300
H	-0.84520900	-4.30238900	4.03290500
C	4.12735000	0.86402600	-0.57995600
C	5.01445800	1.00216000	0.49674500
C	4.48905700	1.35758100	-1.85073500
C	6.25763900	1.62969900	0.31802300
H	4.77119400	0.63663900	1.49478300
C	5.72438200	1.97309100	-2.02150200
H	3.79688400	1.25024400	-2.68630000
C	6.61530400	2.11581000	-0.94905500
H	2.22145700	0.53128200	1.60812200
H	-4.06289700	-0.76879100	-0.69300000
H	7.57675100	2.60342800	-1.10992600
H	-6.00387600	4.91085500	0.34055400
H	-6.84813700	2.59527100	0.43810600
O	-3.38200100	5.55511300	-0.10777100
C	-4.20420200	6.70078500	0.07659500
H	-3.54277100	7.57190500	-0.01771800
H	-4.99254300	6.76078800	-0.69244200
H	-4.66985400	6.70716700	1.07632500
H	6.01204700	2.35447600	-3.00419500
O	7.04383700	1.71935400	1.42409600
C	8.31487300	2.34773800	1.31883100
H	8.22005800	3.40147100	1.00709400
H	8.76171300	2.30782700	2.32083500
H	8.97116400	1.81663200	0.60909600
**B3**			
C	2.11375400	0.07780000	-0.09426700
C	2.27512500	-0.30047700	1.37048400
C	2.60269000	-1.76441500	1.51979400
C	-3.03527600	-0.91616200	0.88942500
C	-2.97673300	-1.39540500	-0.54634400
C	-1.89085200	-2.39602100	-0.92161400
H	2.99388000	0.32109500	1.90654600
H	-3.95386900	-1.84707600	-0.79694500
Pd	0.00153100	-2.16938000	-0.14998900
O	-1.65182700	-3.44295100	-0.17333400
O	-2.14275000	-1.14274400	1.69705900
O	1.22212300	-0.46502400	-0.75677300

O	1.88988100	-2.65286500	1.08930700
C	-1.67131200	-2.50393600	-2.41331700
O	-1.87692300	-1.58924600	-3.19790500
O	-1.22435900	-3.70787500	-2.79521600
C	-0.91480800	-3.88534000	-4.19656000
C	-0.45440300	-5.31059500	-4.39777800
H	-0.13541200	-3.15942000	-4.47779800
H	-1.81553500	-3.65871400	-4.78797200
H	-0.21501000	-5.47195200	-5.46024500
H	0.44891600	-5.52429700	-3.80519000
H	-1.24112500	-6.02548300	-4.10993500
C	-4.23658000	-0.12092100	1.31595300
C	-5.23417100	0.28014200	0.41415000
C	-4.34538400	0.24470700	2.67009600
C	-6.33959900	1.02617300	0.85147100
H	-5.17093700	0.02697200	-0.64474800
C	-5.44929600	0.97253200	3.10814200
H	-3.56486200	-0.06128500	3.36779900
C	-6.45428100	1.35117800	2.21308300
H	-5.54701300	1.23510400	4.16411800
C	3.91890300	-2.11964600	2.23592200
O	4.71127300	-1.27429700	2.59366700
O	4.05015300	-3.42154500	2.38844200
C	5.25739500	-3.90152200	3.04615500
C	5.18769300	-5.40765200	3.10622500
H	5.30137500	-3.44438100	4.04633800
H	6.12228800	-3.54532700	2.46626400
H	6.09308700	-5.79097100	3.60132000
H	4.31140500	-5.74190900	3.68275400
H	5.13579600	-5.84339400	2.09655400
C	2.99943300	1.08083100	-0.72593300
C	4.19043700	1.50908500	-0.11483900
C	2.63745300	1.60115600	-1.98545100
C	5.02776800	2.43519500	-0.75349000
H	4.50336700	1.11040800	0.85051600
C	3.46793600	2.52334000	-2.61441300
H	1.71029800	1.27453000	-2.45745700
C	4.66625800	2.92923800	-2.01790500
H	3.19697900	2.92163300	-3.59464800
H	5.33050900	3.61191600	-2.54882500
H	-7.33608000	1.87845800	2.57852600
H	1.27463800	-0.16626400	1.82037000
H	-2.90118700	-0.50822200	-1.19537000
N	-7.33242500	1.36250700	-0.09581000

H	-7.49621000	0.67146300	-0.82688600
N	6.23990200	2.78439400	-0.12017500
H	6.69599500	2.03876100	0.40416100
C	6.90527600	3.98139200	-0.03697500
O	8.02641900	4.00947200	0.48024100
C	-8.10042100	2.48800000	-0.24615400
O	-8.99728200	2.49382100	-1.09628500
C	6.23891100	5.21664600	-0.53835900
C	6.71493500	6.54271800	0.02297300
C	7.07694700	6.17310600	-1.37534700
H	5.18113500	5.14168300	-0.78964300
H	7.49934100	6.49960000	0.78238700
H	5.94696400	7.30427700	0.18117400
H	6.57165900	6.67294000	-2.20596200
H	8.10913500	5.86898800	-1.57062200
C	-7.80801500	3.68456100	0.59335600
C	-8.28057100	5.02967200	0.07700500
C	-8.99260100	4.40106700	1.22656700
H	-6.87864500	3.67550400	1.16238400
H	-8.78143900	5.03169600	-0.89406100
H	-7.62282000	5.88094700	0.27059000
H	-8.84401400	4.80636100	2.23095500
H	-9.97869100	3.96687000	1.03897300
**B4**			
C	-2.71491600	-0.28867800	-0.20216100
C	-2.77520900	-1.26601100	-1.36414000
C	-1.81150200	-2.42816500	-1.24416900
C	2.40910800	-0.27539700	-0.09516500
C	2.43945500	-0.76574100	1.34153800
C	1.49247500	-1.91717600	1.61275500
H	-3.79431400	-1.67692000	-1.45522400
H	3.45685700	-1.09026100	1.61394700
Pd	-0.15093700	-1.99009700	0.14624000
O	1.58139500	-3.01424500	0.96974600
O	1.43838200	-0.55943100	-0.81899800
O	-1.75454400	-0.32872200	0.58741900
O	-1.87275400	-3.24599100	-0.26760400
C	0.73561900	-1.87349800	2.92733200
O	0.59745600	-0.85847400	3.58835400
O	0.25260900	-3.06531200	3.27952200
C	-0.52582800	-3.13092700	4.49968200
C	-0.96208600	-4.56308600	4.70026600
H	-1.38445100	-2.44866500	4.39943500
H	0.09909600	-2.77027800	5.33126200

H	-1.55520400	-4.63725700	5.62478400
H	-1.58649400	-4.90926800	3.86189600
H	-0.09342700	-5.23338800	4.79348000
C	-1.06687400	-2.81881100	-2.50665800
O	-0.95840800	-2.08673400	-3.47558200
O	-0.55757500	-4.04962000	-2.44119500
C	0.21519500	-4.50864000	-3.57738700
C	0.68908900	-5.91269000	-3.28435600
H	1.05557900	-3.81310400	-3.72801400
H	-0.42411000	-4.46800900	-4.47282400
H	1.27961500	-6.28354000	-4.13628700
H	1.32656000	-5.93732400	-2.38677400
H	-0.16151000	-6.59519000	-3.13180200
H	-2.57848300	-0.70603600	-2.29073600
H	2.19568100	0.08692900	1.99378900
C	3.47392300	0.55751300	-0.62701800
C	4.57985100	0.94070400	0.06536200
H	3.31530800	0.87343700	-1.66162300
H	4.69404100	0.60117800	1.09950600
C	-3.74244900	0.72243000	-0.02814200
C	-4.82990100	0.86537100	-0.83367900
H	-3.56790100	1.39439600	0.81659800
H	-4.95992400	0.16422400	-1.66354600
C	5.66866300	1.77422300	-0.41872800
C	5.71826200	2.30526700	-1.72687400
C	6.73792800	2.06972100	0.45693100
C	6.79268100	3.08339300	-2.13828700
H	4.91154500	2.10395400	-2.43392400
C	7.81799100	2.84676900	0.05479000
H	6.73725000	1.67800500	1.47680400
C	7.85602600	3.36111900	-1.26230100
H	6.83276200	3.48537900	-3.15448800
C	-5.87570000	1.86747100	-0.73210000
C	-5.87759100	2.87628500	0.26666000
C	-6.92826000	1.83780100	-1.66816700
C	-6.89964700	3.81267400	0.31351300
H	-5.07210900	2.91817300	0.99897200
C	-7.95728200	2.77836900	-1.62422000
H	-6.93531700	1.06499000	-2.44032600
C	-7.95320600	3.76904500	-0.64205500
O	8.86743500	3.06588400	0.91765700
O	8.93438700	4.09130000	-1.62442500
H	8.84598000	4.37978200	-2.54790800
O	-8.94333300	4.68861800	-0.59383000

H	-8.76192600	5.29553900	0.14640700
C	8.93592600	4.37403600	1.49113600
H	9.07273500	5.15005500	0.72096100
H	8.02512600	4.59730600	2.07280800
H	9.80399500	4.38034200	2.16487700
O	-7.01667800	4.82580000	1.21497100
C	-6.02250700	4.97276100	2.22122000
H	-5.03008200	5.15582500	1.77694700
H	-6.31709700	5.84183600	2.82318300
H	-5.97445800	4.08039800	2.86715400
H	-8.77460100	2.76055900	-2.34810500

Table S3. Docking parameters.

Target	Binding site	Grid center (x, y, z)	Grid size (dx, dy, dz)
DNA	Minor groove	(15.32, 21.26, 17.82)	(25.37, 29.46, 29.53)
	Intercallation gap	(0.29, 16.47, 37.65)	(24.34, 26.22, 28.72)
HSA	Sudlow Site I (Subdomain IIA)	(64.44, 29.21, 67.72)	(55.02, 64.54, 43.28)
	Sudlow Site II (Subdomain IIIA)	(47.57, 36.38, 71.19)	(34.18, 41.02, 46.02)
